# AMPK-activated BAP1 regulates pVHL stability and tumor-suppressive functions

**DOI:** 10.1038/s41418-025-01590-9

**Published:** 2025-09-27

**Authors:** Mei Li, Lei Huang, Jiayi Chen, Tangming Guan, Yalei Wen, Yingjie Zhu, Xiao Yang, Caishi Zhang, Xiuqing Ma, Rui Wan, Yuanqiao He, Yang Zhou, Yan Song, Haoxing Zhang, Tongzheng Liu

**Affiliations:** 1 Department of Clinical Pharmacy, Central People’s Hospital of Zhanjiang, Zhanjiang, China; 2 State Key Laboratory of Bioactive Molecules and Druggability Assessment/International Cooperative Laboratory of Traditional Chinese Medicine Modernization and Innovative Drug Development of Ministry of Education (MOE) of China/College of Pharmacy, Jinan University, Guangzhou, China; 3 Research Institute for Maternal and Child Health, The Affiliated Guangdong Second Provincial General Hospital, Jinan University, Guangzhou, China; 4 Center of Laboratory Animal Science, Nanchang University, Jiangxi Province Key Laboratory of Laboratory Animal and Nanchang Royo Biotech Co. Ltd, Nanchang, China; 5 Department of Pathology, National Cancer Center/National Clinical Research Center for Cancer/Cancer Hospital, Chinese Academy of Medical Sciences and Peking Union Medical College, Beijing, China; 6 Guangdong Provincial Key Laboratory of Genome Stability and Disease Prevention, College of Life Sciences and Oceanography, Shenzhen University, Shenzhen, China

**Keywords:** Tumour-suppressor proteins, Ubiquitylation

## Abstract

The von Hippel-Lindau (VHL) protein (pVHL) functions as a potent tumor suppressor by mediating the degradation or inactivation of various substrates, including HIFα and Akt. However, pVHL is frequently downregulated in numerous cancers harboring wild-type *VHL*, and underlying mechanisms remains elusive. Aberrant glucose metabolism is a hallmark of cancer, driving tumor progression and therapeutic resistance. Despite this, the connection between glucose homoeostasis and pVHL turnover and functions has yet to be defined. In this study, we demonstrate that dysregulated glucose metabolism destabilizes pVHL in pancreatic ductal adenocarcinoma (PDAC), colorectal, and ovarian cancer cells. Mechanistically, energy stress induced by glucose starvation, 2-deoxyglucose (2-DG), or metformin activates AMP-activated protein kinase (AMPK), which subsequently phosphorylates and activates BAP1, a deubiquitinase whose specific function in targeting pVHL for deubiquitination and stabilization had not been previously characterized. Specifically, AMPKα phosphorylates BAP1 at residues S123, S469, and S583, enhancing the interaction between BAP1 and pVHL and promoting pVHL stabilization and tumor-suppressive function both in vitro and in vivo. Conversely, disrupting BAP1 phosphorylation through AMPKα depletion or reconstitution with a phosphorylation-defective BAP1 mutant (S123A/S469A/S583A) abolishes the BAP1-pVHL interaction, leading to impaired pVHL stabilization and accelerated tumor progression in cancer cell lines and patient-derived xenograft models. Clinically, our analysis reveals a positive correlation between levels of phosphorylated AMPKα (p-AMPKα), phosphorylated Ser123-BAP1 (pSer123-BAP1), and pVHL levels in PDAC, colorectal cancer, and ovarian cancer specimens. Collectively, these findings elucidate a novel mechanism linking dysregulated glucose metabolism to compromised function of the BAP1-pVHL tumor-suppressive axis. Our results suggest that therapeutic strategies designed to activate this pathway may represent a promising approach for treating cancers characterized by downregulated wild-type *VHL* and aberrant glucose metabolism.

## Introduction

The von Hippel-Lindau (VHL) tumor suppressor protein (pVHL) exerts its anti-tumorigenic functions through two distinct mechanisms. First, it acts as a core component of an E3 ubiquitin ligase complex, targeting hydroxylated substrates including HIFα [1], ZHX2 [2], and SFMBT1 [3] for proteasomal degradation. Second, pVHL serves as a scaffolding protein that negatively regulates oncogenic signaling pathways, including Akt and NF-κB pathways [4, 5]. Despite these critical roles, pVHL expression is frequently downregulated in cancers harboring wild-type *VHL*. However, the underlying mechanism responsible for this downregulation remains poorly understood.

Aberrant glucose metabolism is a hallmark of human cancers, characterized by the upregulations of key glycolytic enzymes [6] and glucose transporters such as GLUT1 and GLUT3 [7, 8]. These metabolic dysregulations result in increased intracellular glucose levels and enhanced glucose utilization in solid tumors, which supports the energetic and biosynthetic demand of solid tumors, driving tumor progression and contributing to therapeutic resistance [9, 10]. Emerging evidence suggests that hypoxia-inducible factor alpha (HIFα) plays a vital role in modulating metabolic reprogramming, activating oncogenic signaling pathways, and promoting cancer stem cell (CSC) phenotypes as well as therapeutic resistance [11]. Furthermore, hyperactivation of the Akt pathway, which is commonly observed in human cancers, regulates metabolism by inducing or activating glucose transporters and key metabolic enzymes, thereby facilitating tumor progression [12, 13]. Given its regulatory effects on substrates such as HIFα and Akt, pVHL has been implicated in the modulation of cancer metabolism. However, it remains unclear whether and how aberrant metabolism affects pVHL turnover and its suppressive activity in human cancers.

In this study, we uncover a novel mechanism of metabolic regulation affecting pVHL turnover in pancreatic ductal adenocarcinoma (PDAC), colorectal cancer, and ovarian cancer cells. We demonstrate that the activation of AMP-activated protein kinase (AMPK) is crucial for stabilizing pVHL under energy stresses conditions induced by glucose starvation, 2-deoxy-D-glucose (2-DG), and metformin. Mechanistically, AMPK directly phosphorylates BAP1, enhancing its deubiquitinase activity towards pVHL, which in turn suppresses tumor progression both in vitro and in vivo. Importantly, a strong positive correlation between p-AMPK, pSer123-BAP1, and pVHL levels was observed in PDAC, colorectal cancer, and ovarian cancer specimens. Collectively, our findings suggest that glucose-mediated energy homeostasis is an upstream regulator of the tumor-suppressive activity of the BAP1-pVHL axis. This highlights the therapeutic potential of targeting this axis for the management of cancers characterized by downregulated wild-type *VHL* and aberrant glucose metabolism.

## Materials and methods

### Cell culture, plasmids, and antibodies

HEK293T, PANC-1, BxPC3, AsPC1, MIAPaCa-2 cells and other lines were obtained from ATCC (American Type Culture Collection). All cell lines were mycoplasma-free and authenticated by short tandem repeat DNA profiling analysis. Pan02 cells were kindly shared by Dr Jihui Hao (Tianjin Medical University Cancer Institute and Hospital). The AMPKα wild-type and AMPKα DKO MEFs cells were kindly shared by Dr Wei Liu (Zhejiang University). HEK293T, PANC-1, Pan02, MIAPaCa-2 cells were cultured in DMEM (Gibco) medium supplemented with 10% FBS (TransGen Biotech). AsPC1 and BxPC3 cells were cultured in RPMI-1640 (Gibco) medium supplemented with 10% FBS (TransGen Biotech). All cells were maintained in a humidified cell incubator with 5% CO_2_ at 37 °C.

*BAP1*, *VHL*, *AMPKα1,* and *AMPKα2* were cloned into pIRES-EGFP, pLV.3-FLAG, pCMV-HA, pET28a and pGEX4T-1 vectors, respectively. All site mutants were generated by site-directed mutagenesis and identified by sequencing. *BAP1, VHL, and AMPKα* short hairpin RNAs (shRNAs) were cloned following the protocol-pLKO.1 -TRC cloning vector from Addgene. Sequences for *VHL* shRNA are 5′-CCCTATTAGATACACTTCTTA-3′. The sequences for AMPKα shRNA are 5′- ATGATGTCAGATGGTGAATTT-3′, which could specifically deplete both homo sapiens and Mus musculus AMPKα1/α2. The sequences for homo sapiens BAP1 shRNA#1 and #2 are 5′-CGTCCGTGATTGATGATGATA-3′ and 5′-CCACAACTACGATGAGTTCAT-3′. The sequences for Mus musculus BAP1 shRNA#3 and #4 are 5′-CCACAACTATGACGAGTTTAT-3′ and 5′-CGTCTGTGATTGATGATGATA-3′.

Antibodies anti-pVHL (68547, dilution: 1:1000), anti-AMPKα (D5A2) (5831, dilution: 1:1000), anti-phospho-AMPK substrate (5759, dilution: 1:1000), anti-phospho-AMPKα (Thr172) (2535, dilution: 1:1000), anti-phospho-ACC1 (Ser79) (11818, dilution: 1:1000), anti-ACC1 (4190, dilution: 1:1000), anti-Akt (pan) (2920, dilution: 1:1000) and anti-phospho-Akt (Thr308) (13038, dilution: 1:1000) were purchased from Cell Signaling Technology. Antibodies anti-BAP1 (C-4) (sc-28383, dilution: 1:500) and anti-ubiquitin (sc-8017, dilution: 1:500) were purchased from Santa Cruz Biotechnology. Antibodies anti-FLAG (F3165, dilution: 1:1000), anti-HA (H3663, dilution: 1:1000) and anti-β-actin (A1978, dilution: 1:5000) were purchased from Sigma-Aldrich. Antibodies anti-HIF1α (A300-286A, dilution: 1:500) and anti-HIF2α (BL-95-1A2, dilution: 1:500) were purchased from Bethyl Laboratories. Antibody anti-pSer123-BAP1 (4443-M, custom antibody, dilution: 1:1000) was generated from Shanghai YouKe Biotechnology Co., Ltd.

### Denaturating Ni-NTA pulldown

Cells were transfected with indicated constructs and collected cell pellets were lysed in 8 M urea, 0.1 M NaH2PO4, 300 mM NaCl, and 0.01 M Tris (pH 8.0). Lysates were briefly sonicated to shear DNA and incubated with Ni-NTA agarose beads (Invitrogen, CA, USA) for 2 h at 4 °C. Beads were washed for 4 times with 8 M urea, 0.1 M NaH2PO4, 300 mM NaCl, and 0.01 M Tris (pH 8.0). Input and beads were boiled in loading buffer and subjected to SDS–polyacrylamide gel electrophoresis and immunoblotting.

### Denaturing immunoprecipitation for ubiquitination

Cells were lysed in 100 μl 62.5 mM Tris-HCl (pH 6.8), 10% glycerol, 2% SDS, 1 mM iodoacetamide and 20 mM NEM, boiled for 15 min, diluted 10 times with NETN buffer containing protease inhibitors, 20 mM NEM and 1 mM iodoacetamide, then centrifuged to remove cell debris. Cell extracts were subjected to immunoprecipitation with the indicated antibodies and blotted.

### Animal studies

Female BALB/c nude mice (5–7-week-old) were obtained from Jicui Yaokang Biotechnology Co., Ltd., Jiangsu, China and were housed under specific-pathogen-free condition in the Animal Center of Jinan University. For subcutaneous xenografting, PANC-1 cells (1 × 10^6^) were injected subcutaneously in mouse flanks (*n* = 6). Tumor volumes were measured three times weekly by using a vernier caliper to measure the short diameter and long diameter of the tumor. Tumor volumes were calculated using the following formula: width^2^ × length × 0.4 (mm^3^). When tumor volumes reached about 100 mm^3^, mice were administered saline or metformin (100 mg/kg) every two days. Gemcitabine (50 mg/kg) was administered for three times weekly. After tumors had grown for designated time, all mice were euthanized and tumors were harvested. For the liver metastasis study, PANC-1 cells (5 × 10^6^) were transfected as indicated and injected into the pancreatic tail of female nude mice orthotopically (*n* = 6). Mice were sacrificed after 6 weeks, and the number of metastatic liver nodules was counted and quantified. For patient-derived tumor xenografts (PDXs), pancreatic tumors for the fifth generation of mice (P5) were purchased from Nanchang Royo Biotech Co., Ltd. PDAC tumors were cut into pieces of 3 × 3 × 3 mm^3^, and tumor tissues were pushed under skin of mice by trochar. Tumor volumes were measured three times weekly by using a vernier caliper. When tumor volumes reached 30 mm^3^, mice were randomly divided into different groups. Lentiviruses were produced in HEK293T cells, filtered through a 0.45 µm filter, and concentrated using a PEG-8000 (DH230-1). The xenograft tumors were intratumorally injected with control, shBAP1 #1, shBAP1 #2, shBAP1 #2 + FLAG-VHL or shBAP1#2 + FLAG-BAP1 WT, shBAP1#2 + FLAG-BAP1 3A lentivirus at a dose of 1 × 10^8^ pfu/100 μL per mouse every three days for three times to knockdown or overexpress BAP1 expression in the tumor [14–16]. Mice were administered saline or metformin (100 mg/kg) every two days. Gemcitabine (50 mg/kg) was administered for three times weekly. After tumors had grown for the designated time, all mice were euthanized and tumors were harvested. All animal experiments were performed in accordance with a protocol approved by the Institutional Animal Care and Use Committee of the Jinan University (20210330-04, 20220308-06, and 20230205-08).

### Quantification and statistical analysis

For cell proliferation and cell viability assays, all data are analyzed by GraphPad Prism 9.3. Each experiment was independently repeated for three times, following the principle of repeatability. In the animal study, data represent as the mean ± s.d. of six mice. Statistical differences between two groups were assessed using a two-tailed Student’s *t*-test, while comparisons among multiple groups were performed using one-way ANOVA followed by Tukey’s post hoc test. All figures report *p*-values, effect sizes, and 95% confidence intervals. *p < 0.05, **p < 0.01, ***p < 0.001. *p < 0.05 is considered statistically significant.

## Results

### Glucose homeostasis regulates pVHL in an AMPK-dependent manner

**Fig. 1:**
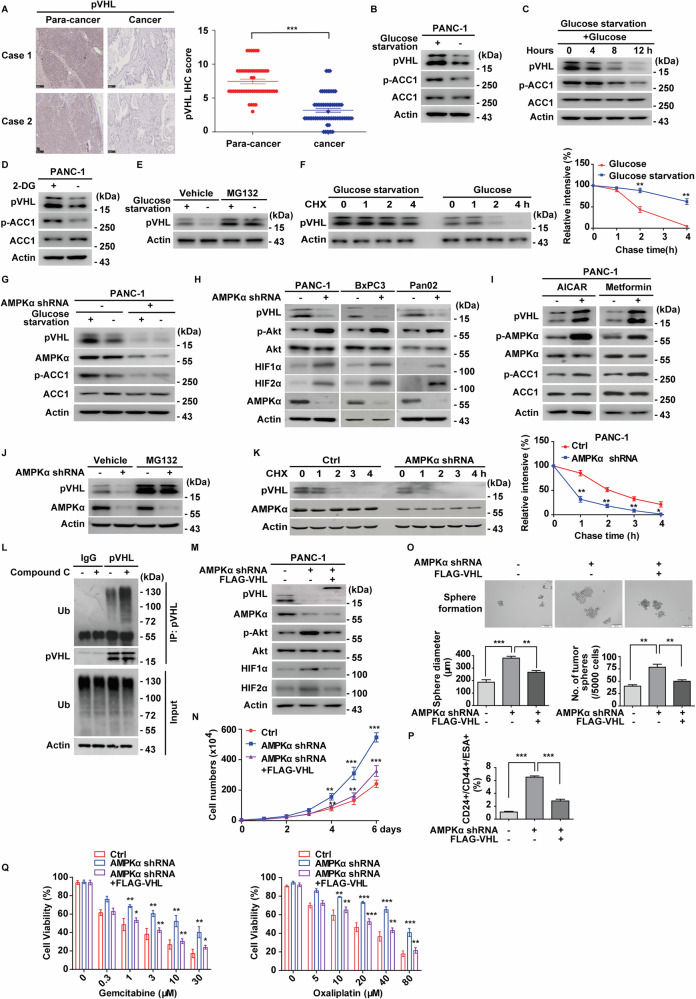
Glucose homeostasis regulates pVHL in an AMPK-dependent manner. **A** Representative immunohistochemical staining of pVHL in para-cancer or cancer samples of PDAC (*n* = 64). Scale bar, 250 μm. The IHC score was calculated by combining the quantity score (percentage of positive stained tissues) with the staining intensity score and quantified in right panel. Statistical significance was assessed by two-tailed Student’s *t*-test. **B** PANC-1 cells were glucose starved (1 mM glucose) for 12 h, and western blot was performed with indicated antibodies. Data are representative of three independent experiments. **C** PANC-1 cells were glucose starved (1 mM glucose) for 12 h and then stimulated with glucose (25 mM) for indicated periods of time. Western blot was performed with indicated antibodies. Data are representative of three independent experiments. **D** PANC-1 cells were treated with 5 mM 2-DG for 24 h, and western blot was performed with indicated antibodies. Data are representative of three independent experiments. **E** PANC-1 cells were glucose starved (1 mM glucose) for 12 h and then stimulated with glucose (25 mM) for 12 h. Cells were treated with vehicle or MG132 (10 μM) for 10 h before harvest, and western blot was performed with indicated antibodies. Data are representative of three independent experiments. **F** Cycloheximide pulse-chase assay was performed in PANC-1 cells cultured in the absence or presence of glucose starvation. The relative level of pVHL to actin was measured by ImageJ. Results represent the mean ± s.d. of three independent experiments (biological replicates). Statistical significance was determined by a two-tailed Student’s *t*-test. **G** PANC-1 cells stably expressing control or AMPKα shRNA were cultured with glucose starvation (1 mM glucose) or normal DMEM for 12 h, and western blot was performed with indicated antibodies. Data are representative of three independent experiments. **H** PANC-1, BxPC3, and Pan02 murine pancreatic adenocarcinoma cells stably expressing control or AMPKα shRNA were generated, and western blot was performed with indicated antibodies. Data are representative of three independent experiments. **I** PANC-1 cells were treated with AMPK activator AICAR (1 mM) for 4 h, or metformin (1 mM) for 24 h, respectively. Western blot was then performed with indicated antibodies. Data are representative of three independent experiments. **J** PANC-1 cells stably expressing control or AMPKα shRNA were treated with vehicle or MG132 (10 μM) for 10 h before harvest, and western blot was performed with indicated antibodies. Data are representative of three independent experiments. **K** Cycloheximide pulse-chase assay was performed in PANC-1 cells as in (**H**). The relative level of pVHL to actin was measured by ImageJ. Results represent the mean ± s.d. of three independent experiments (biological replicates). Statistical significance was determined by a two-tailed Student’s *t*-test. **L** PANC-1 cells were pretreated with vehicle or Compound C (5 μM) for 4 h, then pretreated with vehicle or MG132 (10 μM) for 10 h before harvest, and cell lysates were subjected to immunoprecipitation with IgG or anti-pVHL antibodies. The ubiquitination of pVHL was measured by western blot with anti-ubiquitin antibody. Data are representative of three independent experiments. **M** PANC-1 cells stably expressing AMPKα shRNA were transfected with vector or FLAG-VHL. Western blot was performed with indicated antibodies. Data are representative of three independent experiments. **N** Cell proliferation assay was performed in PANC-1 cells as in (**M**). Results represent the mean ± s.d. of three independent experiments (biological replicates). Statistical significance was determined by one-way ANOVA followed by Tukey’s multiple comparisons test. **O** Tumor sphere formation abilities of PANC-1 cells as in (**M**), were measured by tumor sphere formation assays and results were quantified. Scale bars, 200 μm. Results represent the mean ± s.d. of three independent experiments (biological replicates). Statistical significance was determined by one-way ANOVA followed by Tukey’s multiple comparisons test. **P** Graphic representation of the CD24/^+^CD44^+^/ESA^+^ population from cells described in (**M**) was examined by FACS analysis. Results represent the mean ± s.d. of three independent experiments (biological replicates). Statistical significance was determined by one-way ANOVA followed by Tukey’s multiple comparisons test. **Q** Cells as in (**M**), were treated with indicated concentrations of gemcitabine or oxaliplatin, and cell viability was determined. Results represent the mean ± s.d. of three independent experiments (biological replicates). Statistical significance was determined by one-way ANOVA followed by Tukey’s multiple comparisons test.

Despite its well-established role as a tumor suppressor in hereditary VHL disease and clear-cell renal carcinomas (ccRCCs) [17], the status and function of pVHL remain poorly understood in PDAC, one of the most lethal cancers. Our analysis of public data from cBioPortal database revealed that the *VHL* gene is predominantly wild-type in PDAC (Fig. S1A). Additionally, we demonstrated that stable overexpression of *VHL* in BxPC3 and PANC-1 cells significantly reduced cell proliferation and sensitized these cells to chemotherapeutic agents, including gemcitabine or oxaliplatin (Fig. S1B–D). Conversely, knockdown of *VHL* produced opposite effects (Fig. S1E–G). We also observed that *VHL* overexpression in PDAC cells resulted in a marked reduction of HIF1α and HIF2α protein levels, as well as decreased phosphorylation of Akt, while depletion of pVHL had the opposite effects (Fig. S1B and E). Importantly, we found that pVHL was significantly downregulated in PDAC specimens compared to adjacent non-cancerous tissues (Fig. 1A). Moreover, high *VHL* expression was positively correlated with better overall survival (OS) based on Kaplan–Meier Plotter databases analysis (Fig. S1H). These results strongly support the tumor-suppressive role for pVHL in PDAC and stabilizing pVHL could be a promising therapeutic strategy in PDAC.

While pVHL downregulation is frequently observed in wild-type *VHL* cancers, including PDAC [18, 19], the underlying mechanisms remain incompletely understood. Given pVHL’s established role in controlling key metabolic regulators such as HIFα and Akt [20], we investigated whether deregulated metabolism could, in turn, influence pVHL expression in PDAC. To explore this, we examined the effects of various energy stress conditions on pVHL levels. We found that glucose starvation, but not amino acid deficiency, significantly increased pVHL protein levels in PDAC cells, coinciding with elevated phosphorylation of ACC1 (Figs. 1B and S1I, J). Reintroducing glucose to glucose-deprived cells led to a marked decrease of pVHL protein levels (Fig. 1C). Additionally, treatment with 2-deoxyglucose (2-DG), a non-metabolizable glucose analog that induces cellular energy stress by inhibiting glycolysis, resulted in a significant increase in pVHL protein levels (Figs. 1D and S1K). The metabolic control of pVHL by glucose starvation appears to be a common mechanism, as similar results were observed in other cancer cells harboring wild-type *VHL*, including colorectal cancer cells (LoVo) and ovarian cancer cells (SKOV3) (Fig. S1L). Glucose-mediated regulation of pVHL occurs post-transcriptionally, as *VHL* mRNA levels remained unchanged during glucose starvation or 2-DG treatment (Fig. S1M). Proteasome inhibition with MG132 prevented glucose-induced pVHL degradation (Fig. 1E), while cycloheximide chase assays confirmed enhanced pVHL stability with glucose deprivation or 2-DG treatment (Figs. 1F and S1N). These findings indicate that glucose availability regulates pVHL turnover through a proteasome-dependent mechanism.

AMPK, a central cellular energy sensor, regulates cell metabolism and growth in response to changes in cellular energy status [21]. To delineate the regulatory mechanisms governing pVHL turnover under glucose deprivation-induced energy stress, we first examined the involvement of AMPKα. As showed in Figs. 1G and S1O, glucose starvation increased pVHL levels in wild-type but not AMPKα-deficient MEFs or PANC-1 cells. In contrast, SIRT1, a known metabolic regulator [22] and reciprocal modulator of AMPK signaling in liver and muscle [23, 24], did not impact pVHL stability. Genetic ablation of SIRT1 in MEFs and knockdown in PANC-1 had no effect on pVHL protein levels, AMPK phosphorylation (Fig. S2A, B), or pVHL ubiquitination (Fig. S2C). Together, these results identify AMPKα, rather than SIRT1, as the primary regulator of pVHL stability under glucose deprivation in PDAC.

We further explored the influence of AMPKα on pVHL stability and downstream signaling pathways. In AMPKα-depleted PDAC cells, we observed a significant decrease of pVHL protein levels, accompanied by increased levels of HIF1α, HIF2α, and Akt phosphorylation (Fig. 1H). Conversely, AMPK activation via AICAR, metformin [25], or expression of a catalytical-active mutant AMPKα2 CA (1–312, T172D) [26] increased pVHL levels and suppressed HIF1α, HIF2α and Akt phosphorylation. In contrast, the kinase-dead mutant AMPKα2 K45R [27] had no effect (Figs. 1I and S2D, E). These effects occurred at the post-transcriptional level, as AMPK activity did not alter *VHL* mRNA levels (Fig. S2F–H). Notably, proteasome inhibition restored pVHL levels in AMPKα-deficient cells (Fig. 1J). Additionally, AMPKα depletion shortened the half-life of pVHL, whereas metformin treatment prolonged it (Figs. 1K and S2I). We next assessed whether AMPKα affected pVHL ubiquitination. As shown in the Fig. 1L, pharmacological inhibition of AMPKα by Compound C significantly increased pVHL ubiquitination levels. We further confirmed that AMPKα depletion elevated pVHL ubiquitination, consistent with the effect observed with Compound C (Fig. S2J), suggesting that the observed results of Compound C are primarily due to AMPK inhibition. Additionally, overexpression of the catalytically active AMPKα2 CA mutant or activation of AMPK by metformin dramatically decreased pVHL ubiquitination (Fig. S2K, L). Collectively, these results demonstrate that AMPKα stabilizes pVHL by affecting its ubiquitination level.

Next, we explored the functional consequence of AMPKα-mediated pVHL stabilization. In both PANC-1 and BxPC3 cells, AMPKα depletion significantly increased cellular proliferation, an effect that was largely mitigated by reconstituting pVHL in AMPKα-deficient cells (Figs. 1M, N and S2M, N). Given that the accumulation or activation of HIFα, Akt, and other pVHL substrates have been linked to enhanced cancer-like stem cells (CSC) characteristics [28], we investigated the role of AMPKα in regulating the stemness of pancreatic cancer cells. As shown in Fig. 1O, P, AMPKα depletion in PANC-1 cells markedly increased sphere formation efficiency and the proportion of CD24^+^/CD44^+^/ESA^+^ cells, recognized markers of pancreatic CSCs [29]. Notably, reconstitution of pVHL in AMPKα-deficient cells largely reversed this effect. Considering that CSCs contribute to chemo-resistance, we further evaluated the effect of AMPKα on PDAC cell sensitivity to chemotherapeutic drugs. As shown in Figs. 1Q and S2O, AMPKα depletion in PANC-1 and BxPC3 cells significantly reduced cellular sensitivity to gemcitabine and oxaliplatin. However, reconstitution of pVHL in AMPKα-deficient cells largely restored sensitivity. In summary, our results demonstrate that AMPKα plays a critical role in stabilizing pVHL, thereby suppressing cell proliferation and enhancing sensitivity to chemotherapy in PDAC.

### Identification of BAP1 as the bona fide deubiquitinase of pVHL

AMPK is known to phosphorylate and regulate the degradation of various substrates, including PD-L1, CRY1, PLIN2, and TXNIP [30–33]. However, we did not detect any interaction between AMPK and pVHL, nor evidence of pVHL phosphorylation by AMPK (Fig. S3A, B), suggesting that pVHL is unlikely to be a direct substrate of AMPK. Since WSB1 is a known E3 ligase that targets pVHL for ubiquitination and degradation, we next investigated whether AMPKα regulates pVHL by modulating its interaction with WSB1. However, as shown in Fig. S3C, D, AMPKα depletion or activation via metformin or glucose starvation did not affect the interaction between pVHL and WSB1.

**Fig. 2:**
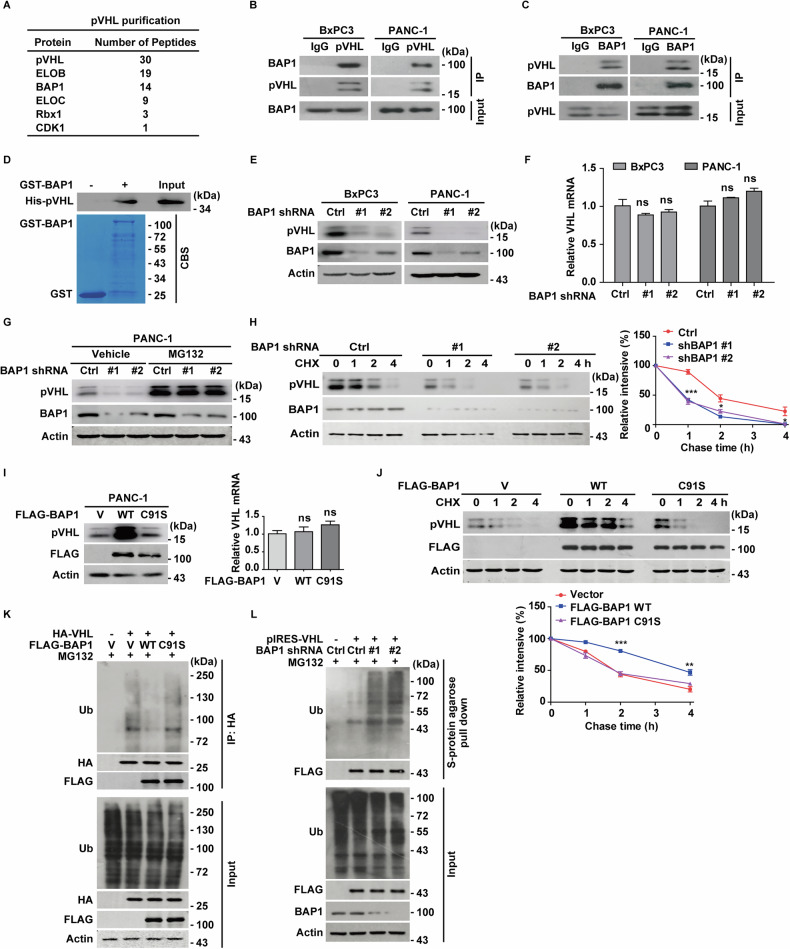
Identification of BAP1 as the bona fide deubiquitinase of pVHL. **A** List of pVHL-associated proteins identified by mass spectrometry analysis. BxPC3 cells stably expressing FLAG-VHL were generated and anti-FLAG-immunoprecipates were subjected to mass spectrometric analysis. **B** BxPC3 and PANC-1 cell lysates were subjected to immunoprecipitation with IgG or anti-pVHL antibodies. The immunoprecipitates were blotted with indicated antibodies. Data are representative of three independent experiments. **C** BxPC3 and PANC-1 cell lysates were subjected to immunoprecipitation with IgG or anti-BAP1 antibodies. The immunoprecipitates were blotted with indicated antibodies. Data are representative of three independent experiments. **D** Purified recombinant GST and GST-BAP1 were incubated with purified His-pVHL in vitro and the direct interaction between BAP1 and pVHL was examined. CBS, Coomassie blue staining. Data are representative of three independent experiments. **E** BxPC3 and PANC-1 cells stably expressing control or BAP1 shRNAs (#1 and #2) were generated and western blot was performed with indicated antibodies. Data are representative of three independent experiments. **F** Total RNA was isolated, and reverse transcribed into cDNA from BxPC3 and PANC-1 cells in (**E**). The mRNA level of *VHL* was determined by quantitative PCR. The relative levels of mRNA were normalized to GAPDH transcript levels. Results represent the mean ± s.d. of three independent experiments (biological replicates). Statistical significance was determined by one-way ANOVA followed by Tukey’s multiple comparisons test. **G** PANC-1 cells stably expressing control or BAP1 shRNAs were treated with vehicle or MG132 (10 μM) for 10 h before harvest, and western blot was performed with indicated antibodies. Data are representative of three independent experiments. **H** Cycloheximide pulse-chase assay was performed in PANC-1 cells; The relative level of pVHL to actin was measured by ImageJ. Results represent the mean ± s.d. of three independent experiments (biological replicates). Statistical significance was determined by one-way ANOVA followed by Tukey’s multiple comparisons test. **I** PANC-1 cells stably expressing vector, FLAG-BAP1 WT or the C91S mutant, were generated, and western blot was performed with indicated antibodies. The mRNA level of *VHL* was determined by quantitative PCR. The relative levels of mRNA were normalized to GAPDH transcript levels. Results represent the mean ± s.d. of three independent experiments (biological replicates). **J** Cycloheximide pulse-chase assay was performed in cells as in (**I**). The relative level of pVHL to actin was measured by ImageJ. Results represent the mean ± s.d. of three independent experiments (biological replicates). Statistical significance was determined by one-way ANOVA followed by Tukey’s multiple comparisons test. **K** HEK293T cells stably expressing vector, FLAG-BAP1 WT or FLAG-BAP1 C91S were transfected with vector or pCMV-HA-VHL, then treated with MG132 (10 μM) for 10 h before harvest. Cell lysates were subjected to immunoprecipitation with anti-HA magnetic beads and the ubiquitination of pVHL was measured by western blot with anti-ubiquitin antibody. Data are representative of three independent experiments. **L** HEK293T cells stably expressing control or BAP1 shRNAs were transfected with vector or pIRES-VHL, then were treated with MG132 (10 μM) for 10 h before harvest. Cell lysates were pull-downed by S-protein agaroses and the ubiquitination of pVHL was measured by western blot with anti-ubiquitin antibody.

To identify the potential linker between AMPKα and pVHL, we performed tandem affinity purification and mass spectrometry analysis in BxPC3 cells stably expressing FLAG-VHL. In addition to components of the ubiquitin E3 ligase complex, including Elongin B (ELOB), Elongin C (ELOC), and Rbx1, as well as the previously identified pVHL-interacting protein CDK1 [18], we identified the deubiquitinase BAP1 as a potential pVHL-interacting protein (Fig. 2A). This interaction was further validated endogenously in BxPC3 and PANC-1 cells (Fig. 2B, C). Additionally, purified GST-BAP1, but not GST alone, pulled down His-pVHL in vitro, confirming a direct interaction between BAP1 and pVHL (Fig. 2D). Notably, cellular fractionation analysis revealed that BAP1 is distributed in both nuclear and cytoplasmic compartments, with its interaction with pVHL predominantly occurring in the cytoplasm (Fig. S3E), consistent with the established subcellular localization of pVHL [34].

While BAP1 has been reported to regulate expressions of PTEN, LKB1, and SLC7A11 in prostate cancer, lung cancer, and clear cell renal carcinoma [35–37], respectively, none of these substrates were affected in BAP1-depleted PDAC cells (Fig. S4A). Similarly, AMPKα depletion in MEFs or knockdown/inhibition in PDAC cells did not affect LATS2 or YAP1 protein levels (Fig. S4B–D), contrasting with previous findings where BAP1 loss reduced LATS2 and increased YAP1 expression in Bap1^fl/fl^-Kras^lsl/+^-Pdx1.cre pancreatic tissues [38]. Furthermore, modulation of AMPKα activity by inhibition (Compound C), activation (metformin), or glucose starvation did not significantly impair the BAP1-LATS2 interaction (Fig. S4E–G). These findings underscore the context- and substrate-specific functions of BAP1 [8, 35, 39].

Given that the bona fide deubiquitinase of pVHL has yet to be identified, we next investigated the potential role of BAP1 in regulating pVHL ubiquitination and stability. As shown in Fig. 2E, BAP1 depletion in BxPC3 and PANC-1 cells significantly reduced pVHL protein levels. Similar results were observed in murine pancreatic adenocarcinoma cell line Pan02, as well as in other PDAC cell lines, including AsPC1 and MIAPaCa-2 (Fig. S4H, I). Notably, reconstitution with BAP1 WT restored pVHL abundance in BAP1-deficient or mutant H226 and H2452 cells (Fig. S4J). Consistently, BAP1 knockdown in PANC-1 cells substantially decreased pVHL protein levels, and this effect was rescued by ectopic expression of BAP1 WT but not the catalytically inactive C91S (CS) mutant (Fig. S4K). Although OTUD6B has been shown to protect pVHL from ubiquitination and degradation by promoting the formation of more CBC^VHL^ ligase complex in an enzyme-independent mechanism [19], BAP1 knockdown had no effect on OTUD6B or WSB1 protein levels, nor did it affect their interaction with pVHL (Fig. S4L, M). Collectively, these results identify BAP1 as a critical deubiquitinase that maintain pVHL stability in a manner dependent on its catalytic activity.

Further investigation revealed BAP1 did not affect *VHL* transcription, but rather inhibited the proteasome-dependent degradation of pVHL (Figs. 2F, G and S4N). Cycloheximide pulse-chase assay demonstrated reduced stability of pVHL protein in BAP1-depleted cells (Figs. 2H and S4O). Conversely, overexpression of BAP1 WT, but not C91S, significantly extended the half-life of pVHL without affecting its mRNA levels (Figs. 2I, J and S4P). These results prompted us to investigate whether BAP1 acts directly as the deubiquitinase of pVHL. Ubiquitination assay conducted under different conditions demonstrated that BAP1 WT, but not the C91S, significantly decreased pVHL ubiquitination, underscoring the importance of BAP1’s enzymatic activity in regulating pVHL stability (Fig. 2K). Additionally, BAP1 depletion markedly increased pVHL ubiquitination (Figs. 2L and S4Q). Collectively, these data reveal BAP1-mediated deubiquitination as a critical mechanism controlling pVHL stability in PDAC cells. We next examined the specific ubiquitin linkages cleaved by BAP1 on pVHL. As shown in Fig. S4R, pVHL was ubiquitinated via both K48-specific and K63-specific chains, but BAP1 selectively catalyzed the cleavage of K48-linked polyubiquitin chains and not K63-linked chains (Fig. S4S, T).

The ubiquitination of pVHL was mainly located in the M3 fragment (156-C terminus) (Fig. S5A). Systematic lysine mutation and ubiquitination assay identified K159 and K171 as primary ubiquitination sites, as the K159R/K171R double mutant (2KR) almost completely abrogated ubiquitination (Fig. S5B). Additionally, we generated VHL mutants containing only one intact lysine residue, with all other lysines replaced by arginine. As shown in Fig. S5C, BAP1 WT significantly reduced the ubiquitination level of the VHL K159 and K171 mutants but had no effect on the K196 mutant. These results suggested that K159 and K171 are the primary ubiquitination sites on pVHL targeted by BAP1for deubiquitination. Furthermore, the double mutant K159R/K171R was substantially more stable than *VHL* WT (Fig. S5D). Overexpression of BAP1 significantly stabilized *VHL* WT but had no effect on the half-life of the K159R/K171R double mutant (Fig. S5E, F). These results suggest that BAP1 is the bona fide deubiquitinase responsible for deubiquitinating and stabilizing pVHL protein.

### BAP1 regulates PDAC progression through stabilizing pVHL

**Fig. 3:**
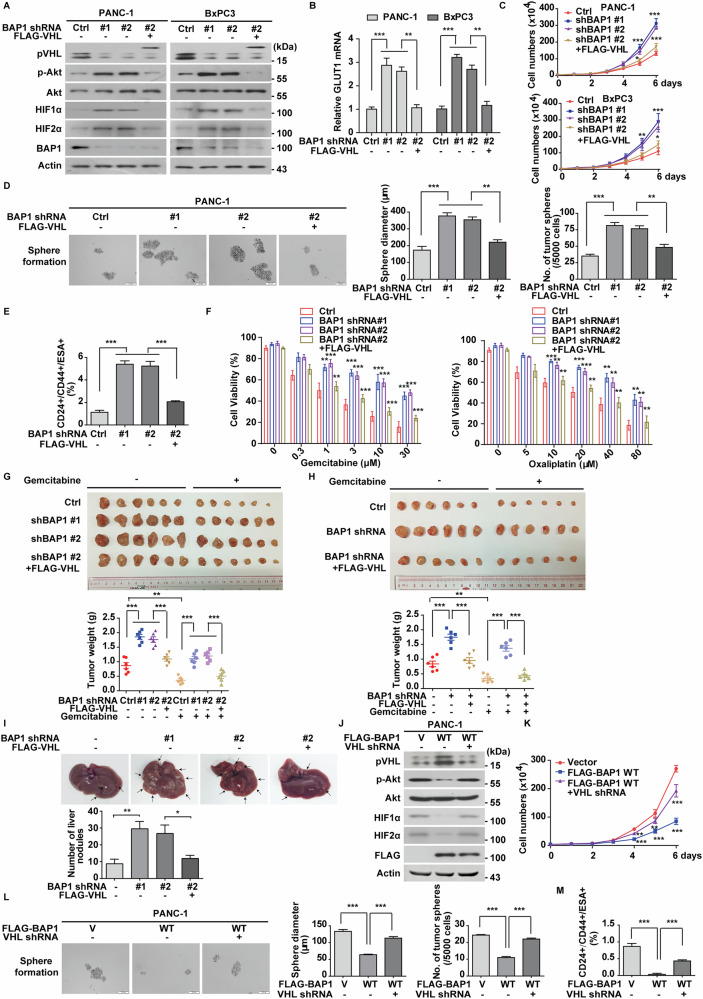
BAP1 regulates PDAC progression through stabilizing pVHL. **A** PANC-1 and BxPC3 cells stably expressing BAP1 shRNAs were transfected with vector or FLAG-VHL. Western blot was performed with indicated antibodies. Data are representative of three independent experiments. **B** Relative expression of *GLUT1* of cells as in (**A**), were determined by quantitative PCR. Results represent the mean ± s.d. of three independent experiments (biological replicates). Statistical significance was determined by one-way ANOVA followed by Tukey’s multiple comparisons test. **C** Cell proliferation assay of cells as in (**A**), was performed. Results represent the mean ± s.d. of three independent experiments (biological replicates). Statistical significance was determined by one-way ANOVA followed by Tukey’s multiple comparisons test. **D** Tumor sphere formation abilities in PANC-1 cells as in (**A**), were measured by tumor sphere formation assays. Scale bars, 200 μm. Results were quantified. Results represent the mean ± s.d. of three independent experiments (biological replicates). Statistical significance was determined by one-way ANOVA followed by Tukey’s multiple comparisons test. **E** Graphic representation of the CD24^+^/CD44^+^/ESA^+^ population of PANC-1 cells as in (**A**), was examined by FACS analysis. Results represent the mean ± s.d. of three independent experiments (biological replicates). Statistical significance was determined by one-way ANOVA followed by Tukey’s multiple comparisons test. **F** PANC-1 cells as in (**A**), were treated with indicated concentrations of gemcitabine or oxaliplatin, and cell viability was determined. Results represent the mean ± s.d. of three independent experiments (biological replicates). Statistical significance was determined by one-way ANOVA followed by Tukey’s multiple comparisons test. **G** PANC-1 cells (1 × 10^6^) as in (**A**), were subcutaneously implanted into nude mice. When tumor volume reached 100 mm^3^, mice were treated with saline or gemcitabine (50 mg/kg three times a week), respectively (*n* = 6 per group). Xenograft tumors were dissected, and tumor weights were measured in lower panel. Results represent the mean ± s.d. from six mice. Statistical significance was determined by one-way ANOVA followed by Tukey’s multiple comparisons test. **H** PDAC patient-derived tumor xenografts (PDXs) were subcutaneously implanted into nude mice and xenograft were injected with lentivirus expressing indicated constructs when tumor volume reached 30 mm^3^. Mice were then treated with saline or gemcitabine (50 mg/kg three times a week), respectively (*n* = 6 per group). Xenograft tumors were dissected, and tumor weights were measured in lower panel. Results represent the mean ± s.d. from six mice. Statistical significance was determined by one-way ANOVA followed by Tukey’s multiple comparisons test. **I** PANC-1 cells expressing control or BAP1 shRNAs were stably reconstituted with vector or FLAG-VHL and injected into the pancreatic tail of female nude mice orthotopically (*n* = 6 per group). Mice were sacrificed and metastatic nodules on liver were counted. Representative images and quantitative analysis of liver metastatic nodules were showed in lower panel. Results represent the mean ± SD from six mice. Statistical significance was determined by one-way ANOVA followed by Tukey’s multiple comparisons test. **J** PANC-1 cells stably expressing FLAG-BAP1 were infected with lentivirus expressing control or VHL shRNA, and western blot was performed with indicated antibodies. Data are representative of three independent experiments. **K** Cell proliferation assay was performed in PANC-1 cells as in (**J**). Results represent the mean ± s.d. of three independent experiments (biological replicates). Statistical significance was determined by one-way ANOVA followed by Tukey’s multiple comparisons test. **L** Tumor sphere formation abilities of PANC-1 cells as in (**J**), were measured by tumor sphere formation assays and results were quantified. Scale bars, 200 μm. Results represent the mean ± s.d. of three independent experiments (biological replicates). Statistical significance was determined by one-way ANOVA followed by Tukey’s multiple comparisons test. **M** Graphic representation of the CD24^+/^CD44^+^/ESA^+^ population of PANC-1 cells as in (**J**), was examined by FACS analysis. Results represent the mean ± s.d. of three independent experiments (biological replicates). Statistical significance was determined by one-way ANOVA followed by Tukey’s multiple comparisons test.

Considering the well-established tumor-suppressive role of pVHL in human cancers and its deubiquitination by BAP1, we next examined whether BAP1 could inhibit PDAC progression by stabilizing pVHL. As shown in Figs. 3A, B and S6A, depletion of BAP1 in BxPC3 and PANC-1 cells significantly reduced pVHL protein levels, which was accompanied by increased phosphorylation of Akt, elevated levels of HIFα protein, and upregulation of its target genes, such as *GLUT1* and *MMP2*. Notably, these effects were largely reversed by pVHL reconstitution. Furthermore, BAP1 depletion in PDAC cells significantly increased cell proliferation (Fig. 3C), sphere formation efficiency (Fig. 3D), and the proportion of CD24^+^/CD44^+^/ESA^+^ stem cell (Fig. 3E), while decreasing cellular sensitivity to gemcitabine or oxaliplatin (Figs. 3F and S6B). Reconstitution of *VHL* effectively rescued these phenotypes.

In support of these findings, BAP1 depletion in cancer cell line-derived xenograft (CDXs) or two PDAC patient-derived xenografts (PDXs) treated with BAP1 shRNA lentivirus resulted in reduced sensitivity to gemcitabine in a pVHL-dependent manner (Figs. 3G, H and S6C). Additionally, we explored the impact of BAP1 on cancer metastasis. As shown in Fig. 3I, BAP1 depletion significantly promoted liver metastasis of PANC-1 cells, which was markedly inhibited by *VHL* reconstitution. Consistently, the tumor-suppressive activity of overexpressed BAP1 in PDAC cells was largely abrogated when pVHL was absent (Figs. 3J–M and S6D, E). These results reveal a previously uncharacterized tumor-suppressive role of BAP1 in PDAC progression through stabilizing pVHL.

### The N-Terminal catalytic domain of BAP1 is essential pVHL stabilization and tumor-suppressive function in PDAC

**Fig. 4:**
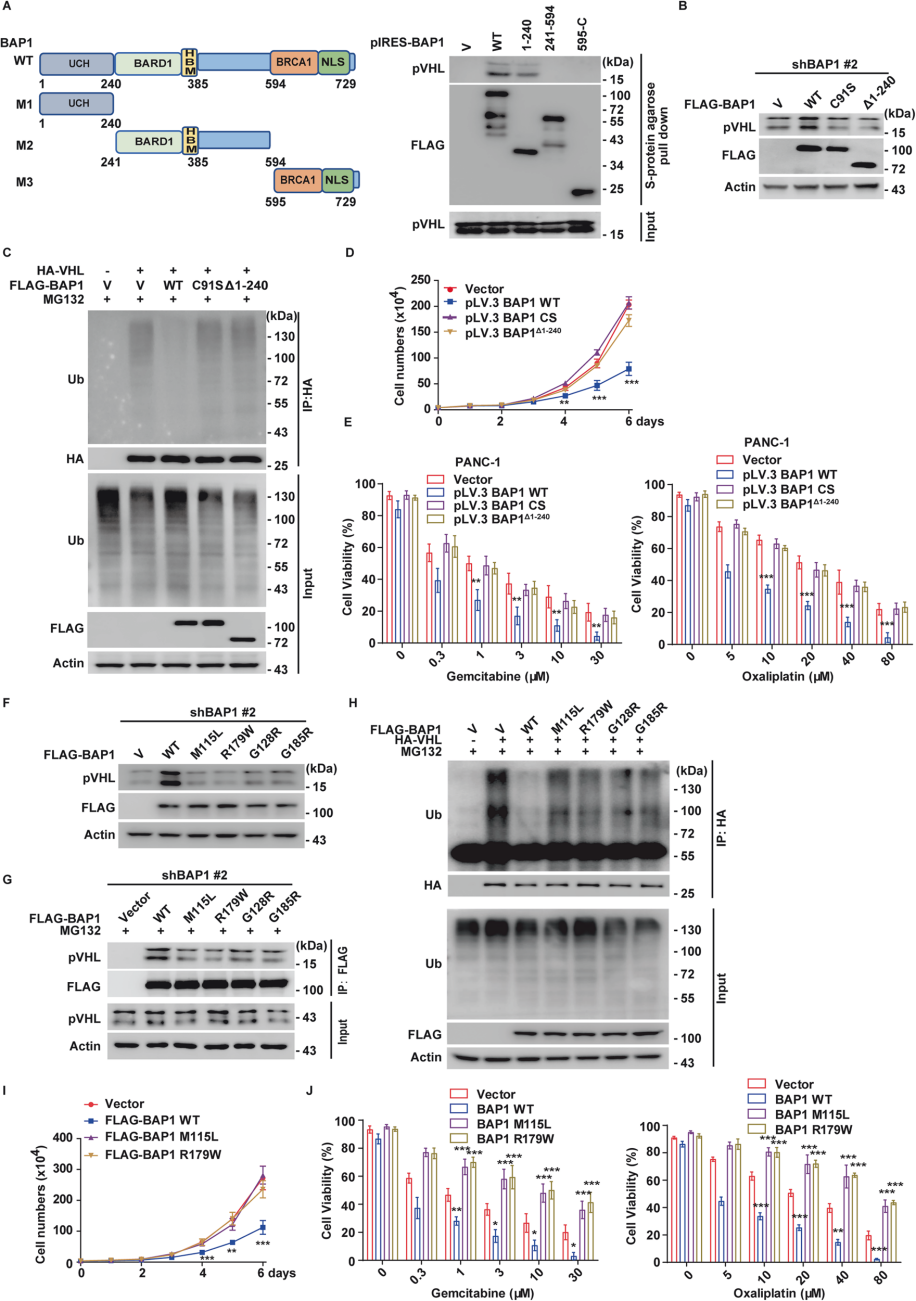
BAP1 N-Terminal domain and catalytic activity are critical for pVHL stabilization and tumor suppression in PDAC. **A** Schematic of BAP1 constructs: WT (wild-type), M1 (1–240 amino acids), M2 (241–594 amino acids), and M3 (595-C terminal) constructs. pIRES vector, pIRES-BAP1 WT or truncation mutants were transfected in HEK293T cells. Cell lysates were subjected to S-protein agarose pulldown, and the interactions with pVHL were detected by western blot. Data are representative of three independent experiments. **B** PANC-1 cells stably expressing BAP1 shRNA were transfected with vector, BAP1 WT, the catalytic-inactive mutant C91S, or the Δ1–240 truncation mutant (lacking the pVHL interaction region). Western blotting was performed with the indicated antibodies. Data are representative of three independent experiments. **C** Cells stably expressing BAP1 shRNA were transfected as indicated. Cell lysates were subjected to immunoprecipitation with anti-HA magnetic beads, and pVHL ubiquitination was assessed by western blotting with anti-ubiquitin antibody. Data are representative of three independent experiments. **D** Cell proliferation assay in PANC-1 cells as in (**B**). Results represent the mean ± s.d. of three independent experiments (biological replicates). Statistical significance was determined by one-way ANOVA followed by Tukey’s multiple comparisons test. **E** PANC-1 cells as in (**B**) were treated with indicated concentrations of gemcitabine or oxaliplatin, and cell viability was measured. Results represent the mean ± s.d. of three independent experiments (biological replicates). Statistical significance was determined by one-way ANOVA followed by Tukey’s multiple comparisons test. **F** Cells stably expressing BAP1 shRNA were transfected with vector, BAP1 WT, or cancer-related mutants. Western blotting was performed with the indicated antibodies. Data are representative of three independent experiments. **G** Cells stably expressing BAP1 shRNA were transfected with vector, BAP1 WT or cancer-related mutants. Cell lysates were subjected to immunoprecipitation with anti-FLAG antibody, and western blotting was performed with the indicated antibodies. Data are representative of three independent experiments. **H** Cells stably expressing BAP1 shRNA were transfected with indicated plasmids. Cell lysates were subjected to immunoprecipitation with anti-HA magnetic beads, and pVHL ubiquitination was measured by western blotting. Data are representative of three independent experiments. **I** Cell proliferation assay was performed in PANC-1 cells as in (**F**). Results represent the mean ± s.d. of three independent experiments (biological replicates). Statistical significance was determined by one-way ANOVA followed by Tukey’s multiple comparisons test. **J** PANC-1 cells as in (**F**) were treated with indicated concentrations of gemcitabine or oxaliplatin, and cell viability was measured. Results represent the mean ± s.d. of three independent experiments (biological replicates). Statistical significance was determined by one-way ANOVA followed by Tukey’s multiple comparisons test.

We identified the N-terminal region of BAP1 (residue 1–240) as critical for its interaction with pVHL (Fig. 4A). Overexpression of BAP1 WT reduced pVHL ubiquitination and increased its protein levels, whereas BAP1^Δ1–240^ or the catalytic-inactive mutant C91S failed to produce these effects (Fig. 4B, C). Notably, these mutants also did not suppress pancreatic cancer cell proliferation or enhance sensitivity to gemcitabine and oxaliplatin (Fig. 4D, E).

To assess the clinical relevance of BAP1’s functional domain interacting with pVHL, we analyzed mutation hotspots within the 1–240 region using cBioPortal database and found that *BAP1* is predominately wild-type in PDAC, with only a few low-frequency mutations, such as M115L and R179W. Functional assay revealed that these mutations, along with other cancer-associated mutants (G128R, G185R [35, 40]), impaired pVHL binding, failed to reduce pVHL ubiquitination, and were unable to restore pVHL protein levels compared to BAP1 WT (Fig. 4F–H). Furthermore, reconstitution of BAP1-depleted cells with M115L or R179W mutants did not replicate the tumor-suppressive effects of BAP1 WT, including inhibition of cell proliferation and restoration of chemosensitivity (Fig. 4I, J). These findings highlight the essential role of the BAP1 N-terminal domain in stabilizing pVHL and mediating its tumor-suppressive effects in PDAC.

### AMPK binds and phosphorylates BAP1

**Fig. 5:**
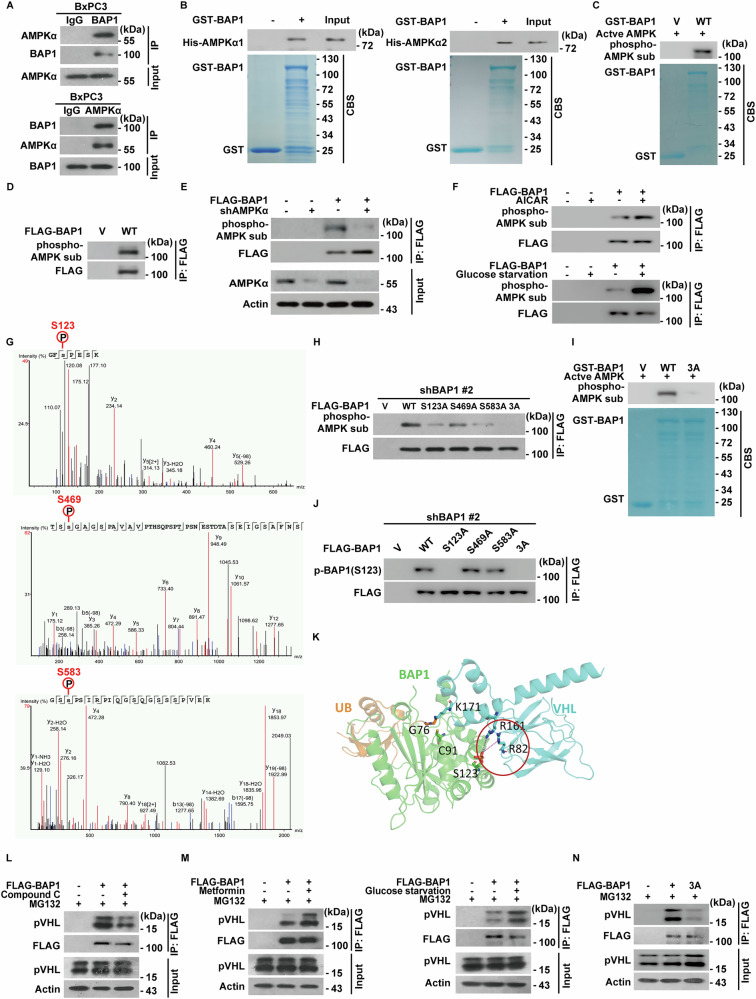
AMPK binds and phosphorylates BAP1. **A** BxPC3 cell lysates were subjected to immunoprecipitation with IgG, anti-BAP1, or anti-AMPKα antibody. The immunoprecipitates were blotted with indicated antibodies. Data are representative of three independent experiments. **B** Purified recombinant GST and GST-BAP1 were incubated with purified His-AMPKα1 or His-AMPKα2 in vitro. The direct interaction between BAP1 and AMPKα was examined. CBS, Coomassie blue staining. Data are representative of three independent experiments. **C** AMPKα phosphorylates BAP1 in vitro. Bacterial purified GST or GST-BAP1 WT was incubated with active AMPK in the kinase buffer at 30 °C for 30 min. The reaction was stopped by the addition of SDS sample buffer containing 5% mercaptoethanol and boiled at 95 °C for 10 min. The phosphorylation of BAP1 were examined using phospho-AMPK substrate antibody. Data are representative of three independent experiments. **D** Vector or FLAG-BAP1 were transfected in PANC-1 cells. Cell lysates were subjected to immunoprecipitation with anti-FLAG antibody and the phosphorylation of BAP1 were examined by using phospho-AMPK substrate antibody. Data are representative of three independent experiments. **E** Vector or FLAG-BAP1 were transfected in control or cells stably expressing AMPKα shRNA. Cell lysates were subjected to immunoprecipitation with anti-FLAG antibody, and the phosphorylation of BAP1 were examined by using phospho-AMPK substrate antibody. Data are representative of three independent experiments. **F** Cells were transfected with indicated plasmids and treated with vehicle, AMPK activator AICAR (1 mM) for 4 h or glucose starvation (1 mM glucose) for 12 h. Cell lysates were subjected to immunoprecipitation with anti-FLAG antibody, and the phosphorylation of BAP1 were examined by using phospho-AMPK substrate antibody. Data are representative of three independent experiments. **G** Identification of AMPK phosphorylation sites on BAP1 by mass spectrometry. Phosphorylation analysis of BAP1 was performed by the Mass Spectrometry Facility. Information on the identified phospho-peptides is shown. **H** Vector, FLAG-BAP1 WT, Ser123A, Ser469A, Ser583A, and the 3A were transfected in cells stably expressing BAP1 shRNA. Cell lysates were subjected to immunoprecipitation with anti-FLAG antibody and the phosphorylation of BAP1 were examined by using phospho-AMPK substrate antibody. Data are representative of three independent experiments. **I** AMPK phosphorylates BAP1 in vitro. Bacterial purified GST, GST-BAP1 WT, or the 3A mutant incubated with active AMPK in the kinase buffer at 30 °C for 30 min. Western blot was performed and the phosphorylation of BAP1 were examined using phospho-AMPK substrate antibody. Data are representative of three independent experiments. **J** Vector, FLAG-BAP1 WT, Ser123A, Ser469A, Ser583A or 3A were transfected in cells stably expressing BAP1 shRNA. Cell lysates were subjected to immunoprecipitation with anti-FLAG antibody and the phosphorylation of BAP1 were examined by using phospho-Ser123-specific antibody. Data are representative of three independent experiments. **K** Predicted structure of ubiquitinated VHL in complexed with BAP1. **L** PANC-1 cells stably expressing vector or FLAG-BAP1 was treated with vehicle or Compound C (5 μM) for 4 h, and treated with MG132 (10 μM) for 10 h before harvest. Cell lysates were subjected to immunoprecipitation with anti-FLAG antibody and western blot was performed. Data are representative of three independent experiments. **M** PANC-1 cells stably expressing vector or FLAG-BAP1 was treated with vehicle, metformin (2 mM) for 24 h or glucose starved (2 mM glucose) for 12 h, and treated with MG132 (10 μM) for 10 h before harvest. Cell lysates were subjected to immunoprecipitation with anti-FLAG antibody and western blot was performed. Data are representative of three independent experiments. **N** PANC-1 cells stably expressing vector, BAP1 WT or its phosphorylation mutant 3A were treated with MG132 (10 μM) for 10 h before harvest. Cell lysates were subjected to immunoprecipitation with anti-FLAG antibody and western blot was performed. Data are representative of three independent experiments.

Given that both AMPKα and BAP1 stabilize pVHL by influencing its ubiquitination, we hypothesized that BAP1 could be the missing link between AMPKα and pVHL. As shown in Fig. 5A, B, we observed both an endogenous interaction between AMPKα and BAP1 in cells, as well as a direct interaction between purified GST-BAP1 and His-AMPKα1/AMPKα2 in a cell-free system. We demonstrated that AMPKα directly phosphorylates BAP1, as active AMPK induced GST-BAP1 phosphorylation in vitro (Fig. 5C). In PANC-1 cells, phospho-AMPK substrate antibody detected BAP1 phosphorylation, which decreased following AMPKα depletion (Fig. 5D, E) and increased upon AMPK activation by AICAR or glucose starvation (Fig. 5F).

To identify potential phosphorylation sites, we co-transfected the catalytically active AMPKα2 CA mutant with HA-BAP1 and performed mass spectrometry analysis. As shown in Fig. 5G, Ser123, Ser469, and Ser583 in BAP1 were identified as phosphorylated sites, aligning with the AMPK substrate consensus motif (L/I/M X K/R XX S/T XXX L/I/M). Notably, mutations of these sites (S123A, S469A, or S583A) led to a partial reduction of BAP1 phosphorylation, as assessed by a phospho-AMPK substrate antibody, while the triple mutation (S123A/S469A/S583A, referred to as 3A) nearly completely abolished phosphorylation (Fig. 5H). Additionally, in vitro kinase assay demonstrated that active AMPK could phosphorylate GST-BAP1 WT, but not the 3A mutant (Fig. 5I).

To further validate BAP1 phosphorylation, we generated a phospho-Ser123-specific antibody. As shown in Fig. 5J, this phospho-specific antibody detected phosphorylation of BAP1 WT but not the S123A or 3A mutants in PANC-1 cells. Antibody specificity was confirmed by immunofluorescence staining (Fig. S7A) and epitope-blocking assays, wherein pre-incubation with the phospho-Ser123 peptide, but not the non-phosphorylated peptide or BSA control, abolished the antibody signal (Fig. S7B). Intriguingly, AMPK activation markedly enhanced both BAP1 phosphorylation and its cytoplasmic accumulation, whereas AMPK inhibition reduced these effects (Fig. S7C, D). These results suggest that AMPKα serves as a key upstream regulator of BAP1 in our experimental model.

Phosphorylation is known to modulate protein-protein interactions or the enzymatic activity of deubiquitinases [41–43]. To explore this further, we modeled the structure of ubiquitinated pVHL in complexed with phosphorylated BAP1 using Maestro (Schrödinger, LLC, New York, NY, 2023) and ROSETTA. Following a 500 ns equilibration, the protein RMSD equilibrated (Fig. S7E). The predicted structure suggests that AMPKα-mediated phosphorylation of BAP1 at Ser123 enhances its interaction with pVHL through electrostatic contacts with Arg82/Arg161 (Fig. 5K). Consistent with this model, the pVHL 2RA mutant (R82A/R161A) exhibited markedly reduced binding to BAP1 (Fig. S7F), leading to a shortened half-life of pVHL and increased its ubiquitination level (Fig. S7G, H). Based on these results, we examined the effects of BAP1 phosphorylation on pVHL recruitment in PDAC cells. As shown in Fig. 5L, inhibition of AMPKα using Compound C significantly reduced the interaction between BAP1 and pVHL, while AMPKα activation by either metformin or glucose starvation markedly increased the BAP1-pVHL interaction in PANC-1 cells (Fig. 5M). Moreover, overexpression of the 3A mutant substantially decreased the BAP1-pVHL interaction compared to BAP1 WT (Fig. 5N), highlighting the critical role of AMPKα-mediated BAP1 phosphorylation in facilitating the BAP1-pVHL interaction.

Previous studies have demonstrated that the ASXM domain of ASXL1/2 interacts with the C-terminal region of BAP1 (residues 599–729), facilitating BAP1-mediated deubiquitination of H2Aub at lysine 119 and regulating chromatin remodeling and transcription in U2OS cells [44]. However, ASXL1/2 knockdown in PDAC cells did not impair BAP1’s ability to deubiquitinate and stabilize pVHL (Fig. S8A, B). Notably, we identified that AMPKα also interacts with the BAP1 C-terminal region (residues 595–729), overlapping with ASXL1/2 binding site (Fig. S8C). Subcellular fractionation and co-immunoprecipitation assay revealed that this AMPKα-BAP1 interaction predominantly occurs in the cytoplasm (Fig. S8D), aligning with previous reports of AMPKα cytoplasmic localization [45]. Importantly, AMPK deficiency in MEFs or metformin treatment in PDAC did not affect nuclear H2AubK119 levels (Fig. S8E, F), indicating that AMPK does not alter BAP1’s nuclear activity.

### AMPKα-mediated phosphorylation of BAP1 regulates the stability and tumor-suppressive function of pVHL

**Fig. 6:**
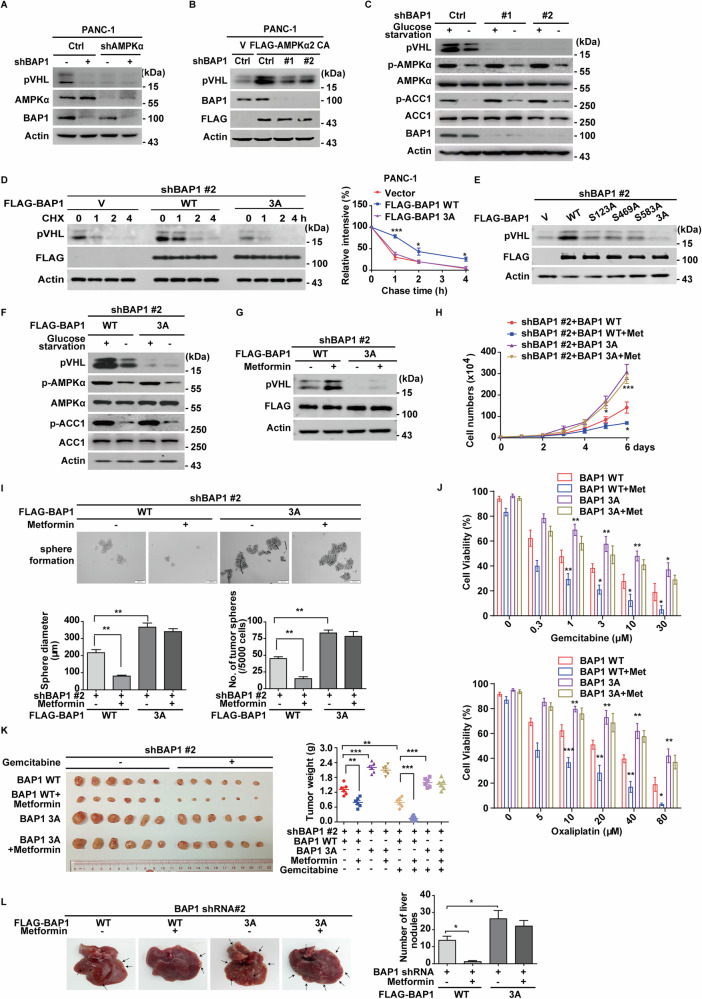
AMPKα-mediated phosphorylation of BAP1 regulates the stability and tumor-suppressive function of pVHL. **A** PANC-1 cells with the depletion of AMPKα, BAP1, or both were subjected to western blot by indicated antibodies. Data are representative of three independent experiments. **B** PANC-1 cells stably expressing FLAG-AMPKα2 CA were infected with lentivirus expressing control or BAP1 shRNAs, and western blot was performed with indicated antibodies. Data are representative of three independent experiments. **C** PANC-1 cells stably expressing control or BAP1 shRNAs were cultured with glucose starvation medium (1 mM glucose) or normal DMEM for 12 h, and western blot was performed with indicated antibodies. Data are representative of three independent experiments. **D** PANC-1 cells stably expressing BAP1 shRNA were transfected with vector, BAP1 WT or its phosphorylation mutant 3A, and cycloheximide pulse-chase assay was performed. Results represent the mean ± s.d. of three independent experiments (biological replicates). Statistical significance was determined by one-way ANOVA followed by Tukey’s multiple comparisons test. **E** PANC-1 cells stably expressing BAP1 shRNA were transfected with indicated plasmids and western blot was performed with indicated antibodies. Data are representative of three independent experiments. **F** PANC-1 cells stably expressing BAP1 shRNA were transfected with BAP1 WT or its phosphorylation mutant 3A and were cultured with glucose starvation medium (1 mM glucose) or normal DMEM for 12 h. Data are representative of three independent experiments. **G** PANC-1 cells stably expressing BAP1 shRNA were transfected with indicated plasmids and treated with vehicle or metformin (1 mM) for 24 h. Western blot was performed with indicated antibodies. Data are representative of three independent experiments. **H** Cell proliferation assay was performed in PANC-1 cells as in (**G**). Results represent the mean ± s.d. of three independent experiments (biological replicates). Statistical significance was determined by one-way ANOVA followed by Tukey’s multiple comparisons test. **I** Tumor sphere formation abilities of PANC-1 cells as in (**G**), were measured by tumor sphere formation assays. Results represent the mean ± s.d. of three independent experiments (biological replicates). Statistical significance was determined by one-way ANOVA followed by Tukey’s multiple comparisons test. **J** PANC-1 cells as in (**G**), were treated with indicated concentrations of gemcitabine or oxaliplatin, and cell viability was determined. Results represent the mean ± s.d. of three independent experiments (biological replicates). Statistical significance was determined by one-way ANOVA followed by Tukey’s multiple comparisons test. **K** PDAC PDXs were subcutaneously implanted into nude mice and xenograft were injected with lentivirus expressing indicated constructs when tumor volume reached 30 mm^3^. Mice were then treated with saline, gemcitabine (50 mg/kg three times a week) or metformin (100 mg/kg every two days) (*n* = 6 per group). Xenograft tumors were dissected, and tumor weights were measured in right panel. Results represent the mean ± s.d. from six mice. Statistical significance was determined by one-way ANOVA followed by Tukey’s multiple comparisons test. **L** PANC-1 cells stably expressing vector, FLAG-BAP1 WT or 3A were injected into the pancreatic tail of female nude mice orthotopically (*n* = 6 per group). Mice were sacrificed and metastatic nodules on liver were counted. Representative images and quantitative analysis of liver surface nodules were showed in right panel. Statistical significance was determined by one-way ANOVA followed by Tukey’s multiple comparisons test.

We next investigated whether BAP1 serves as a potential link between AMPKα and pVHL. As shown in Fig. 6A, knockdown of AMPKα reduced pVHL protein levels, while simultaneous depletion of BAP1 did not further diminish pVHL levels. Furthermore, glucose starvation or overexpression of the catalytically active AMPKα2 CA mutant significantly decreased the ubiquitination of pVHL and increased its protein level in control cells, but these effects were not observed in BAP1-depleted cells (Figs. 6B, C and S8G, H). Overexpression of BAP1 WT significantly decreased pVHL ubiquitination, which was markedly attenuated by the AMPK inhibitor Compound C (Fig. S8I). Additionally, overexpression of BAP1 WT significantly increased pVHL protein levels in BAP1-deficient/mutant cell lines H226 and H2452, which was largely reversed by depletion of AMPKα (Fig. S8J). Conversely, activation of AMPKα by glucose starvation or metformin treatment increased pVHL protein levels in cells reconstituted with BAP1 WT but had no effect in BAP1-deficient/mutant cells (Fig. S8K, L), suggesting that regulation of pVHL by AMPK is primarily mediated by BAP1.

We next investigated effects of the AMPKα-interaction defective mutants BAP1^Δ595-C^ on pVHL ubiquitination and stabilization. As shown in Fig. S9A, B, compared to BAP1 WT, BAP1^Δ595-C^ only partially reduced the ubiquitination of pVHL and moderately increased its stability, whereas the enzymatic mutant C91S completely lacked this function. Functionally, overexpression of BAP1^Δ595-C^ led to a partial suppression of pancreatic cancer cell proliferation and a modest increase in sensitivity to chemotherapeutic agents, as compared to BAP1 WT (Fig. S9C, D). Additionally, a clinically relevant truncation mutation, S509^*^, was identified within the 595-C region of BAP1. Similar to the BAP1^Δ595-C^ mutant, the BAP1S509^*^ mutant exhibited reduced binding to AMPKα, partial retention of pVHL deubiquitination, and moderate stabilization of pVHL (Fig. S9E–G). Functionally, BAP1S509^*^ also only partially suppressed pancreatic cancer cell proliferation (Fig. S9H).

Next, we evaluated whether AMPK-mediated phosphorylation affected BAP1’s ability to stabilize pVHL. As showed in Figs. 6D, E and S9I, reconstitution of BAP1 WT, but not the 3 A mutant, restored pVHL stability in BAP1-depleted cells. Glucose starvation increased pVHL protein levels in cells reconstituted with BAP1 WT but not in those expressing the 3 A mutant (Fig. 6F). Moreover, BAP1 WT markedly decreased pVHL ubiquitination, while the 3 A mutant had minimal effect (Fig. S9J). Treatment with metformin further reduced pVHL ubiquitination level in cells reconstituted with BAP1 WT, but not in cells expressing the 3 A mutant (Fig. S9K).

We further investigated the effects of AMPK-mediated phosphorylation of BAP1 on tumor progression. Reconstitution of BAP1 WT, but not the 3A mutant, in endogenous BAP1-deficient PANC-1 and BxPC3 cells significantly suppressed cell proliferation (Figs. 6G, H and S9L, M) and reduced sphere formation efficiency (Fig. 6I). Additionally, reconstitution of BAP1 WT increased cellular sensitivity to gemcitabine or oxaliplatin (Fig. 6J). Notably, metformin treatment further enhanced cellular sensitivity to gemcitabine in PANC-1-derived xenograft and two PDAC PDXs overexpressing BAP1 WT, but this effect of metformin was not observed in xenografts with the 3A mutant (Figs. 6K and S9N, O). Furthermore, BAP1 WT reconstitution significantly suppressed liver metastases compared to the 3A mutant in endogenous BAP1-deficient cells (Fig. 6L).

### Expression of p-AMPKα and pSer123-BAP1 positively correlates with pVHL levels in PDAC

**Fig. 7:**
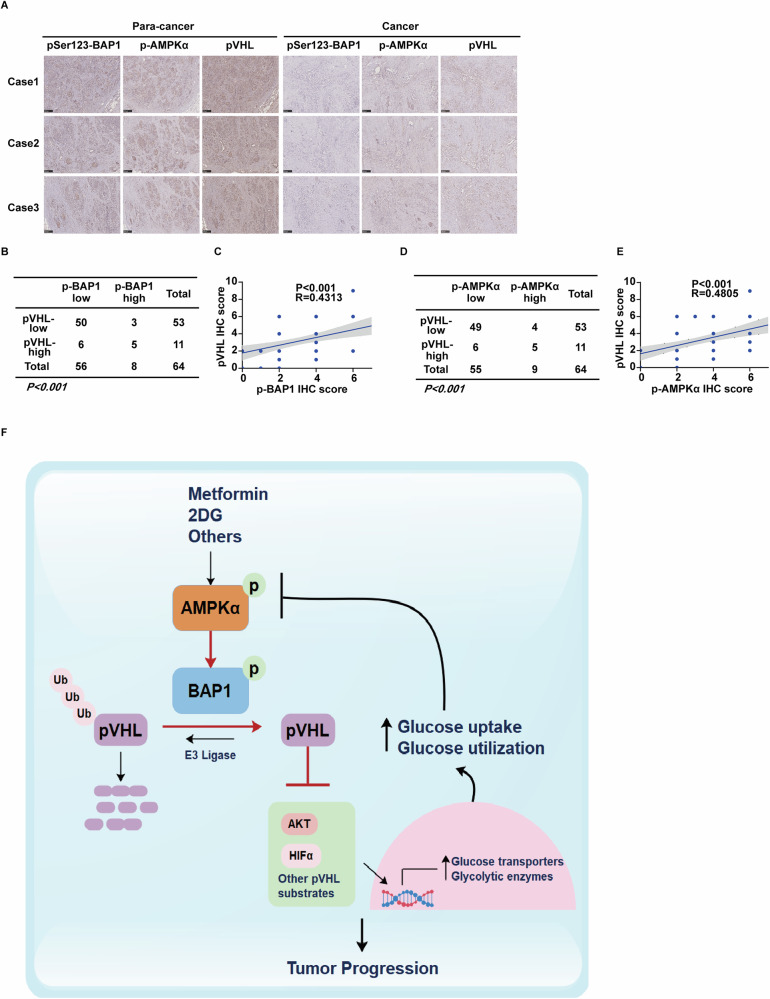
Expression of p-AMPKα and pSer123-BAP1 positively correlates with pVHL levels in PDAC. **A** Representative immunohistochemical staining of pVHL, pSer123-BAP1, and p-AMPKα in PDAC and adjacent normal tissues. Scale bar, 250 μm. **B**, **C** Positive correlation of pVHL expression with pSer123-BAP1 expression in PDAC tissues. **D**, **E** Positive correlation of pVHL expression with p-AMPKα expression in PDAC tissues. **F** The working model to illustrate that AMPKα phosphorylation-dependent activation of BAP1 suppresses tumor growth, metastasis, and chemoresistance in PDAC through stabilizing pVHL.

Our results demonstrate that AMPKα-mediated activation of BAP1 is critical for the stabilization and tumor-suppressive function of pVHL in PDAC. To assess the clinical relevance of this axis, we evaluated 64 PDAC specimens by immunohistochemistry staining. As shown in Fig. 7A, the expression of pVHL, pSer123-BAP1, and p-AMPKα were significantly downregulated in PDAC tissues compared with adjacent non-cancerous tissues. Moreover, expression levels of pSer123-BAP1 were positively correlated with pVHL expression in PDAC samples (Fig. 7B, C), with approximately 89.3% of samples exhibiting low pSer123-BAP1 also showing low pVHL expression. Similarly, pVHL expression positively correlated with levels of p-AMPKα (Fig. 7D, E), with about 89.1% of samples with low p-AMPKα expression displaying low pVHL level. Additionally, low *BAP1* expression was associated with poorer OS and relapse-free survival in PDAC patients (Fig. S10A, B). We further extended these findings to colorectal and ovarian cancers, where positive correlations between p-AMPKα, pSer123-BAP1, and pVHL expression were consistently observed (Fig. S10C–L).

Collectively, these results highlight a crucial role for AMPK in linking glucose-mediated energy stress to the stabilization and tumor-suppressive activity of pVHL. Our study provides preclinical evidence that targeting the AMPKα-BAP1-pVHL axis may represent a promising therapeutic strategy for PDAC and potentially other aggressive cancers harboring wild-type *BAP1* and *VHL* (Fig. 7F).

## Discussion

In this study, we present several novel findings with significant clinical implications regarding the role of glucose metabolism in tumor progression. We identify aberrant glucose status as an upstream event that destabilizes the key tumor suppressor protein pVHL in various cancers. Our data demonstrate that, in response to glucose starvation or activators such as metformin and AICAR, activated AMPK directly phosphorylates BAP1, a deubiquitinase whose specific role in deubiquitinating and stabilizing pVHL had not been previously recognized. This phosphorylation event enhances the stabilization and tumor-suppressive function of pVHL both in vitro and in vivo. Additionally, our histological analyses reveal a positive correlation among p-AMPKα, pSer123-BAP1, and pVHL in PDAC specimens. In summary, these findings underscore a pivotal role for AMPK in linking glucose-mediated energy stress to tumor-suppressive activity of the BAP1-pVHL axis, suggesting that activation of this pathway may represent a promising therapeutic strategy for cancers harboring wild-type *VHL* and aberrant glucose metabolism (Fig. 7F).

Tumor cells can reprogram their metabolism to enhance glucose uptake and utilization through aerobic glycolysis, generating metabolic intermediates and energy essential for tumor growth [46]. Given the established role of pVHL in tumor suppression, particularly through the destabilization or inactivation of HIFα, Akt, and other substrates, it is plausible that pVHL may regulate the expression or activity of key glycolytic enzymes and glucose transporters, thereby modulating cancer metabolism. For instance, inactivation of *VHL* in ccRCC has been demonstrated to upregulate GLUT1 and erythropoietin expressions via a HIFα-dependent pathway, promoting aberrant glucose metabolism and tumor progression [47, 48]. However, whether aberrant glucose metabolism affects pVHL turnover or tumor-suppressive activity remains unknown. In this study, we elucidate a novel role of glucose homeostasis in controlling the turnover of pVHL. Our findings reveal that glucose starvation significantly elevates pVHL protein levels in PDAC cells, with this effect reversing upon glucose reintroduction (Figs. 1B, C and S1I). Similarly, treatment with 2-DG markedly increased pVHL protein levels (Figs. 1D and S1K). Although this study primarily focuses on PDAC, the observed metabolic regulation of pVHL stability in response to glucose levels may represent a broader mechanism, as we observed similar results in other aggressive cancers with wild-type *VHL*, such as colorectal and ovarian cancer (Fig. S1L). Thus, our study provides new mechanistic insights into how aberrant glucose status destabilizes pVHL and promotes tumor progression, which may partially explain the increased incidence and mortality of pancreatic cancer in patients with diabetes mellitus [49–51]. Furthermore, pVHL has broader metabolic roles beyond HIF regulation. Emerging evidence suggests that pVHL modulates multiple metabolic processes independent of HIF. For instance, pVHL has been shown to inhibit nutrient deprivation-induced autophagy initiation by binding hydroxylated Beclin1 [52]. Additionally, pVHL promotes the degradation of hydroxylated SFMBT1 and suppresses the expression of its downstream target, sphingosine kinase 1 (SPHK1), thereby regulating lipid metabolism [3]. These pathways play critical roles in maintaining metabolic homeostasis and regulating tumor cell to adapt diverse stress conditions.

AMPK act as key sensor of intracellular energy status, modulating cellular metabolism and influencing cell fate under stress conditions [25]. Previous studies have demonstrated that AMPK decreases HIF1α protein levels, negatively regulates the Warburg effect in Eμ-Myc lymphomas cells, and suppresses tumor growth [53]. Our results suggest that pVHL may act as the link between AMPKα and HIF1α. Specifically, we observed that AMPKα activation in response to glucose-mediated energy stress promotes pVHL stabilization and HIFα degradation based on the following evidence. First, the knockout or depletion of AMPKα in MEFs and cancer cells markedly decreased pVHL protein levels, while energy stresses, such as glucose starvation, glycolysis blockade (2DG), AICAR and metformin, or the use of catalytically active AMPKα2 CA mutant significantly stabilized pVHL without altering its mRNA levels (Figs. 1B–I, S1I–O, and S2D–H). Second, inhibiting the proteasome with MG132 restored the decreased pVHL protein levels in AMPKα-deficient cells (Fig. 1J). Additionally, targeting AMPKα by its genetic ablation and inhibition significantly increased pVHL ubiquitination levels, whereas overexpression of the catalytically active AMPKα2 CA mutant or activation of AMPK by metformin dramatically decreased this ubiquitination (Figs. 1L and S2J–L). Third, AMPKα suppresses cell proliferation and stemness while sensitizing PDAC cells to chemotherapies in a pVHL-dependent manner (Figs. 1M–Q and S2M–O). Given pVHL’s established tumor-suppressive function, the therapeutic potential of AMPK activation by its agonists [25, 54], calorie restriction [55, 56], or ketogenic diets [30, 57] in stabilizing pVHL and inhibiting tumor growth warrants further investigation in various cancers harboring wild-type *VHL*.

SIRT1 is also a key sensor of intracellular energy status [22]. Several studies have reported SIRT1’s role in regulating HIF1α, although these findings remain highly context-dependent and, in some cases, contradictory. For instance, one study demonstrated that SIRT1 directly deacetylates and inactivates HIF1α in various mouse tissues, reducing acetylation without affecting total HIF1α in HT1080 xenografts [58]. In contrast, another group reported that SIRT1 binds and stabilizes HIF1α via deacetylation during hypoxia [59]. Follow-up work by the same group showed that under chronic hypoxia, elevated NADH levels inactivate SIRT1, leading to increased p300-mediated acetylation of HIF1α at lysine 709. This acetylated form is subsequently recognized by pVHL and targeted for degradation in a manner independently of proline hydroxylation [60]. However, in our PDAC models, we found that genetic ablation or knockdown of SIRT1 had no impact on pVHL and HIF1α protein levels, AMPK phosphorylation, or pVHL ubiquitination levels (Fig. S2A–C). These findings indicate that AMPKα, rather than SIRT1, serves as the primary upstream regulator of pVHL stability in response to glucose-mediated energy stress in PDAC.

Interestingly, our findings reveal that the effect of AMPKα on pVHL stabilization is indirect as we could not detect any direct interaction between purified AMPKα and pVHL, nor could we observe phosphorylation of pVHL by AMPKα (Fig. S3A, B). The ubiquitin E3 ligase WSB1 has been shown to target pVHL for ubiquitination and degradation, promoting melanoma metastasis [61]. Notably, targeting AMPKα did not affect the WSB1-pVHL interaction (Fig. S3C, D), suggesting that unidentified factors may play crucial roles in the regulation of pVHL by AMPKα. Indeed, our study identifies BAP1 as the bona fide deubiquitinase of pVHL, potentially acting as the connector between AMPKα and pVHL. Recent evidence highlights the context-dependent role of BAP1, which functions as either a tumor suppressor or an oncogene depending on cancer type [8, 39]. BAP1 is critical for the efficient recruitment of homologous recombination factors such as BRCA1 and RAD51 in response to ionizing radiation, whereas BAP1 loss or inactivation impairs homologous recombination repair, leading to elevated rates of spontaneous DNA damage and ultimately genomic instability [62]. Beyond its role in DNA repair, BAP1 exerts additional nuclear functions, including deubiquitinating H2Aub on chromatin to repress SLC7A11 expression, thereby promoting lipid peroxidation and ferroptosis in UMRC6 and other renal cancer cell lines [35]. BAP1 also forms the ASXL1-BAP1 complex to activate *Pten* transcription in myeloid malignancies [63], and directly stabilizes PTEN in prostate and hepatocellular carcinomas [36, 64]. In contrast, BAP1 has been reported to promote tumor growth and metastasis via stabilization of KLF5 in breast cancer [8]. However, the role of BAP1 in PDAC is not yet fully understood. Our findings support its tumor-suppressive functions in this context. Firstly, BAP1 interacts with pVHL in cells and in cell-free conditions, directly targeting pVHL for deubiquitination and stabilization in an enzymatic activity-dependent manner (Figs. 2 and S4). Consistently with these results, the depletion of BAP1 in PDAC cells suppresses tumor progression in a pVHL-dependent manner (Figs. 3 and S6). One limitation of our study is that we cannot rule out additional mechanisms involved in BAP1-mediated tumor suppression in PDAC, as ectopic expression of pVHL did not fully rescue functional alterations caused by BAP1 depletion. Although BAP1 knockdown did not alter protein levels of several known BAP1 substrates, including PTEN, LKB1, or SLC7A11 under our experimental conditions (Fig. S4A), it remains possible that other substrates or pathways are involved. For instance, previous studies have shown that heterozygous loss of BAP1 in mice induces chronic pancreatitis and accelerates *Kras*^G12D^-driven pancreatic cancer by inducing genomic instability in a catalytic-independent manner [65]. Thus, the potential involvement of BAP1 substrates or interactors beyond pVHL in PDAC and other malignancies warrants further investigation.

Emerging evidence indicates that BAP1 function can be regulated by post-translational modifications such as phosphorylation and ubiquitination. In response to DNA damage, the phosphorylation of BAP1 at several sites by multiple DNA damage-responsive kinases is crucial for promoting DNA repair and cellular recovery from DNA damage [62]. The atypical ubiquitin ligase UBE2O catalyzes multi-monoubiquitinations of BAP1, which facilitates its cytoplasmic localization and adipocyte differentiation. Intriguingly, this effect can be counteracted by BAP1-mediated autodeubiquitination [66]. Additionally, monoubiquitination of the deubiquitinase adaptor (DEUBAD) domain enhances ASXL2 stabilization, which in turn stimulates BAP1’s deubiquitinating activity and tumor-suppressive function [67]. In this study, we demonstrate a novel AMPK-catalyzed phosphorylation of BAP1 in response to glucose-mediated energy stress, which is crucial for BAP1-mediated deubiquitination of pVHL and the maintenance of its tumor-suppressive activity. Firstly, AMPKα directly binds and phosphorylates BAP1 at Ser123, Ser469, and Ser583 (Fig. 5A–J). Secondly, this AMPKα-mediated phosphorylation of BAP1 significantly promotes the deubiquitination process of pVHL, as evidenced by increased interaction between BAP1 and pVHL (Fig. 5K–N), decreased ubiquitination (Fig. S8H, I), and prolonged half-life of pVHL in cells under glucose-mediated energy stress or in cells reconstituted with BAP1 WT compared to the phosphorylation-deficient 3A mutant (Figs. 6D–F and S9I–K). Importantly, treatment of AMPK activator metformin markedly inhibited cell proliferation, sphere formation efficiency, and increased chemosensitivity in PDAC cells reconstituted with BAP1 WT, but had no effect on cells with the 3A mutant (Figs. 6G–K and S9L–O). These results strongly support the notion that AMPKα-mediated phosphorylation of BAP1 is crucial for pVHL stability and its tumor-suppressive effect. Importantly, our histological analyses reveal that phosphorylated AMPKα and pSer123-BAP1 are positively correlated with pVHL expression in PDAC, colorectal, and ovarian cancer tissues (Figs. 7A–E and S10C–L), underscoring the clinical relevance of the AMPKα-BAP1-pVHL axis.

While our findings highlight the anti-tumor effects of metformin via the AMPKα-BAP1-pVHL axis, therapeutic strategies targeting AMPK must account for potential limitations, including off-target effects and resistance mechanisms. For instance, at clinical relevant doses, metformin exerts AMPK-dependent effects through the PEN2-ATP6AP1-v-ATPase axis [68], whereas at higher concentrations, it inhibits mitochondrial complex I, leading to increased AMP/ATP ratios [69]. However, AMPK-independent effects of metformin, such as mTOR inhibition via REDD1 [70] or Rag GTPase-dependent pathways [71], may compromise specificity. Furthermore, resistance to metformin therapy can also arise through mechanisms such as PTGR1 upregulation via SRF/RUNX3 [72], OCT1 downregulation [73], or activation of DOCK1-RAC1 signaling and mitochondrial electron transport chain mutations [74, 75]. While AMPK activation via metformin holds therapeutic promise, the development of selective AMPK activators, identification of predictive biomarker, and rational combinational strategies will be critical to optimizing its clinical application. In summary, the tumor-suppressive function of the AMPKα-BAP1-pVHL axis underscores its potential as a therapeutic strategy. Further investigation in preclinical models, including *Kras*^G12D^-driven murine models, as well as clinical studies, is warranted to explore the therapeutic benefits of targeting this axis in the treatment of PDAC and other aggressive cancers with wild-type *VHL* and dysregulated glucose metabolism.

## Supplementary information

Supplementary figures

Original western blots

## Data Availability

All data generated or analyzed during this study are available within the article and Supplementary Files. Additional supporting data are available from the corresponding authors upon reasonable request.
