# Supplementary material for: AMPK-activated BAP1 regulates pVHL stability and tumor-suppressive functions
Source: Cell Death Differ. 2025 Sep 27;33(3):447–64. doi: 10.1038/s41418-025-01590-9 (PMC13035857; doi:10.1038/s41418-025-01590-9)
Supplement: Supplementary file 1 — Supplementary figures [file 41418_2025_1590_MOESM1_ESM.docx]

**AMPK-activated BAP1 Regulates pVHL Stability and Tumor-suppressive Functions Supplementary Text1**

**Supplementary Methods**

**Western blot analysis**

Cells were lysed in NETN buffer [300 mM NaCl, 20 mM Tris-HCl (PH 8.0), 0.5% NP-40, 1 mM ethylenediaminetetraacetic acid (EDTA)] containing protease inhibitors [1× protease inhibitor cocktail (Roche), 1 mM sodium orthovanadate, 10 mM β-glycerophosphate, 1 mM phenylmethylsulfonyl fluoride, and 10 mM sodium fluoride]. Proteins were separated by SDS-PAGE gel electrophoresis and transferred to PVDF membranes, then incubated with the indicated primary and secondary antibodies.

**Coimmunoprecipitation assay**

Cells were lysed with NETN buffer containing protease inhibitors in ice for 30 min. Cell lysates were incubated with anti-HA magnetic beads, anti-FLAG affinity gel or S-protein agarose for 2 or 4 hours at 4°C. Following precipitation, immunoprecipitates were washed for 4 times with NETN buffer and then analyzed by immunoblotting.

**Quantitative Real-Time PCR (qRT-PCR)**

Total RNA was isolated using TRIzol (Invitrogen), and 200 ng of RNA was reversed transcribed into cDNA using the FastKing gDNA Dispelling RT SuperMix (TIANGEN, Beijing, China). Real-time quantitative PCR was performed using the FastFire qPCR PreMix (SYBR Green) (TIANGEN). GAPDH was used as an internal control for normalization.

Primer sequences are listed：

*VHL* Forward: CTGCCCGTATGGCTCAACTT; Reverse: GTGTGTCCCTGCATCTCTGAAG.

*MMP2* Forward: TGACTTTCTTGGATCGGGTCG, Reverse: AAGCACCACATCAGATGACTG.

*GLUT1* Forward: CTTTGTGGCCTTCTTTGAAGT; Reverse: CCACACAGTTGCTCCACAT.

*GAPDH* Forward: AATCCCATCACCATCTTCCAG; Reverse: AAATGAGCCCCAGCCTTC.

**Glutathione S-transferase (GST) pulldown assay**

Indicated cDNA was cloned into pGEX4T-1 or pET28a vector and transfected into *Escherichia coli* strain BL21. After the induction by 200 μM Isopropyl β-D-1-thiogalactopyranoside (IPTG) (I6758-1G, Sigma) at 18°C for overnight, GST and GST-fusion proteins were purified by using Pierce Glutathione Agarose (Thermo Scientific). Purified GST or GST fusion proteins bound to Pierce Glutathione Agarose were incubated with His-tagged fusion proteins purified from the *Escherichia coli* strain BL21 at 4°C for 4 h. Then beads were washed with NETN buffer for 4 times, followed by western blot.

***In Vitro* Kinase Assay**

The recombinant GST-BAP1 WT and 3A mutant protein was expressed in *Escherichia coli* strain BL21 and purified using Pierce Glutathione Agarose. The proteins were then eluted with GST elution buffer and purified with Ultrafiltration tube. Purified GST-BAP1 WT and 3A were incubated with recombinant active AMPK (14-840, Millipore) in kinase assay buffer (50 mM Tris pH 8.0, 10 mM MgCl2, 1 mM DTT, 1 mM EDTA, 0.3 mM NaCl, 200 µM AMP and 100 µM ATP). The reaction was carried out at 30°C for 30 min and stopped by the addition of SDS loading buffer, then analyzed by western blot analysis.

**Cell Proliferation Assay**

PANC-1 or BxPC3 (4×10^4^) cells were seeded in 6-well plates and cells were digested with 0.25% trypsin at 37°C at the indicated periods. Cell pellets were collected by centrifugation (3 000 rpm for 5 min), washed by PBS twice, re-suspended in PBS, and then counted under microscope.

**Tumor sphere formation Assay**

Tumor sphere formation assay was performed as previously described ^1^. PANC-1 cells were harvested and suspended as single cells in stem-cell culture medium, which was composed of DMEM/F12, supplemented with 20 ng/mL recombinant human EGF (rhEGF) and 10 ng/mL of basic fibroblast growth factor (bFGF) together with 1×B27. After accurate cell counting, 1 000 cells/well were added to a low adhesion 24-well plate containing 500 μL of stem-cell culture medium, and each group was in 3 wells. After 10 days of incubation, images were taken with an inverted microscope, and the spheres >50 cells were counted.

**Cell viability Assay**

The sensitivity to gemcitabine or oxaliplatin of the PDAC cell lines (PANC-1 and BxPC3) was measured by the trypan blue exclusion method. A total of 2 000 cells in a volume of 100 μL per well were cultured in four replicate wells in 96-well plates in medium containing 10% FBS. Cells were exposed to different concentrations of gemcitabine or oxaliplatin for 48 h and then digested with 0.25% trypsin at 37°C. Cells were resuspended in PBS was diluted 1:1 with 0.4% trypan blue (Solarbio, C0040). After 3 minutes incubation, cells were loaded onto a hemocytometer and counted under a light microscope. The ratio of unstained cell numbers to total cell numbers was reported as the viability percentage for each cell category.

**Flow cytometry analysis**

PANC-1 cells stably expressing AMPKα or BAP1 shRNAs were reconstituted with *VHL* WT and digested with 0.25% trypsin at 37°C. Cell pellets were collected by centrifugation (1500 rpm for 5 min) and then washed by PBS twice. Cells were stained with antibodies anti-CD44-FITC (FHF0442-025, 4A Biotech, dilution: 1:100), anti-CD24-PE (555428, BD Pharmingen, dilution: 1:100) and anti-Ep-CAM-APC (324207, Biolegend, dilution: 1:100) at 4°C in the darkness for 1 h. The pellets were then washed by PBS twice and re-suspended in 500 μL PBS. Flow cytometry analysis was performed with a FACS-LSRFortessa flow cytometer.

**Tandem affinity purification and mass spectrometry analyses**

In brief, tandem affinity purification in cells expressing FLAG-pVHL was conducted by anti-FLAG affinity gel. BxPC3 cells stably expressing FLAG-pVHL were treated with MG132 (10 µM) for 10 hours and cell pellets were then lysed in NETN buffer containing protease inhibitors. 20 µL Anti-FLAG Affinity Gel was added into cell lysates and rotated at 4 °C for 2 h. Anti-FLAG immunoprecipitates were washed three times with cold NETN buffer. The beads were resuspended in 500 μL 6 M urea in PBS, 25 μL of 200 mM DTT in 25 mM NH_4_HCO_3_ buffer was added and the reaction was incubated for 37°C for 30 min. For alkylation, 25 μL of 400 mM IAA in 25 mM NH_4_HCO_3_ buffer was added followed by incubation for 30 min at room temperature in dark. The supernatant was then removed and the beads were washed with 1 mL PBS once. For the digestion, 150 μL 2 M urea in PBS, 150 μL 1 mM CaCl_2_ in 50 mM NH_4_HCO_3_ and 1 μL of trypsin (1.0 μg/μL) were added. The reaction was incubated at 37°C overnight. After evaporation in speedvac, the samples were tested by LC-MS/MS, equipped with an EASY-nLC 1200 HPLC system and QE Plus mass spectrometer (Thermo Fisher Scientific). About 1 μg of each sample was injected into a trap column (75 μm×2 cm, C18, 3 μm, 100 Å, 164535) and an AcclaimPepMapC18 RSLC 75 μm ID×25 cm separation column in an EASY-Spray setting, three injections were loaded for each sample continuously. Peptides were separated with a 70-min gradient (buffer A: 0.1% formic acid in deionized water; buffer B: 0.1% formic acid in 80% acetonitrile with a flow rate of 0.3 μL/min: 51 min of 4-28% B, 5 min of 28-38% B, 4 min of 38-90% B, 5 min of 90-4%B, 5 min of 4%B). Separated peptides were then directly analyzed on the QE Plus in a data-dependent manner. Peptides were ionized by using spray voltage of 2.4 kV and the ion transfer tube temperature was 320 °C, with automatic switching between MS and MS/MS scans using exclusion duration 25 s. Mass spectra were acquired at a resolution of 60,000 with a target value of 3 × 10^6^ ions or a maximum integration time of 20 ms. The scan range was limited from 350 to 1500 m/z. Peptide S44 fragmentation was performed via higher-energy collision dissociation with the energy set to 30%. High-resolution MS2 spectra were acquired with an exclusion duration of 25 s in the QE Plus with a maximum injection time of 40 ms at 17500 resolution (isolation window 1.6 m/z), an AGC target value of 1× 10^5^ and normalized collision energy of 30%. The fixed first m/z was 100, and the isolation window was 1.6 m/z^2^. The raw data were processed by using Proteome Discoverer 2.5 and processed as per default workflow. MS tolerance is 4.5 ppm, and MS/MS tolerance is 20 ppm. Searches were performed against the Homo sapiens uniport canonical 20395 entries 20210516 nm fasta. Reversed database searches were used to evaluate false discovery rate (FDR) of site, peptide and protein identifications. Two missed cleavage sites of trypsin were allowed. The mass spectrometry proteomics data have been deposited to the ProteomeXchange Consortium (https://proteomecentral.proteomexchange.org) via the iProX partner repository^3, 4^ with the dataset identifier: PXD067816.

**Immunofluorescence staining**

Cells were seeded onto glass coverslips for the experiment. Cells were washed with PBS, fixed with 4% formaldehyde for 20 min, permeabilized with 0.1% Triton X-100 for 5 min, washed with PBS for three times, blocked with 0.5% BSA for 1 h, incubated with primary antibodies for overnight at 4℃ and then incubated with secondary antibodies. The primary antibodies used in the immunofluorescence staining was anti-pSer123-BAP1 (4443-M, custom antibody, dilution: 1:1,00). Nuclei were stained with DAPI.

**Nuclear-Cytoplasmic Fractionation**

Nuclear and cytoplasmic protein fractions was prepared using the Nuclear and Cytoplasmic Protein Extraction Kit (Beyotime Biotechnology, P0027). For cytoplasmic extraction, cell pellets were resuspended in 200 μL of Cytoplasmic Extraction Reagent A containing PMSF by vortexing at maximum speed for 5 seconds. After 15 minutes on ice,

10 μL of Cytoplasmic Extraction Reagent B was added, followed by another 5 seconds vortex at maximum speed. The mixture was incubated on ice for 1 minute, vortexed briefly, and centrifuged at 12,000 g for 5 minutes at 4°C. The cytoplasmic supernatant was transferred to pre-chilled tubes. For nuclear extraction, the residual supernatant was completely removed, and the pellet was resuspended in 50 μL of Nuclear Extraction Reagent with PMSF by votexing for 30 seconds at maximum speed. The mixture was further vortexed for 20 seconds every 2 minutes intervals during a 30 minutes ice incubation. After final centrifugation at 12,000 g for 10 minutes at 4°C, the nuclear protein-containing supernatant was collected into pre-cooled tubes for analysis.

**In silico modeling and binding of pSer123-BAP1 to VHL**

The structure of ubiquitin and BAP1 were retrieved from the BAP1-ASXL1 complex bound to chromatosome (PDB ID: 8H1T). The Gly76 of ubiquitin was linked to Lys171 of VHL (PDB ID: 1VCB) and a phosphate group was added to Ser123 of BAP1. The simulations were performed with Desmond. OPLS4 force filed was used for the system. The system was relaxed for 5 ns before 500 ns production simulations and the simulations were run in NPT ensemble at 300 K and 1 atm with default settings.

**Validation of the phosphorylation specificity of anti-pSer123-BAP1 antibody** **​**

To validate the specificity of the anti-pSer123-BAP1 antibody, the pSer123-BAP1 antibody (1 μg) was pre-incubated with 1 mL PBS containing BSA (Ctrl, 10μg), phosphorylated peptide (P-p123, 10 μg), or non-phosphorylated peptide (NP-p123, 10 μg) at 4°C for 2 h prior to immunoblotting of PANC-1 cell lysates.

**Kaplan-Meier survival analysis**

Kaplan-Meier survival analysis was performed using the Kaplan-Meier Plotter online tool (http://kmplot.com) to evaluate the correlation between *BAP1* and the prognosis of clinical PDAC patient survival. The survival curves and corresponding hazard ratios were calculated based on default settings. The analysis was conducted as previously described^5^.

**Immunohistochemical staining**

Pathological tissue sections were obtained from the tissue bank at The First Affiliated Hospital of Jinan University in accordance with the approval document of the Institutional Medical Ethics Committee (Ethics Approval License: JNUKY-2022-097). Tissue samples with anti-phospho-AMPKα (Thr172) (2535, CST, 1:100), anti-pSer123-BAP1 (4443-M, custom antibody, dilution: 1:100) and anti-pVHL (68547, CST, 1:100) antibodies were used for immunohistochemical staining of formalin-fixed paraffin-embedded of pancreatic cancer tissues were incubated out at 4°C for 12 h. The immunostaining was randomly scored by two pathologists. The IHC score was calculated by combining the quantity score (percentage of positive stained tissues) with the staining intensity score. The quantity score ranges from 0 to 4, For example: 0, no immunostaining; 1, 1-24% of tissues are stained; 2, 25-49% are positive; 3, 50-74% are positive; and 4, ≥ 75% of tissues are positive. The staining intensity was scored as: 0 (negative), 1 (weak), 2 (moderate) and 3 (strong). The score for each tissue was calculated by multiplying the quantity with the intensity score (the range of this calculation was therefore 0-12). An IHC score of 9-12 was considered a strong immunoreactivity; 5-8, moderate; 1-4, weak; and 0, negative. Samples with IHC score > 4 were considered to be high, and ≤ 4 were considered to be low. The χ2-test was used for statistical analysis of the correlation between p-AMPKα, pSer123-BAP1 and pVHL.

**Statistical details of confidence interval in main figures**

Fig. 1. **Glucose homeostasis regulates pVHL in an AMPK-dependent manner. A,** ****p*<0.001; [95% CI, -5.098— -3.429]; R^2^=0.4758; effect size= 0.6870519. **F,** 2h glucose vs glucose starvation: ***p*＜0.01; [95% CI, 20.40 — 68.71]; effect size= -0.9021004. 4h glucose vs glucose starvation: ***p*＜0.01; [95% CI, 38.81 — 76.91]; effect size= -0.9603369. **K,** 1h Ctrl vs AMPKα shRNA: ***p*＜0.01; [95% CI, -76.06 — -30.97]; effect size= 0.93731. 2h Ctrl vs AMPKα shRNA: ***p*＜0.01; [95% CI, -49.02 — -17.84]; effect size= 0.9248076. 3h Ctrl vs AMPKα shRNA: ***p*＜0.01; [95% CI, -38.66 — -9.728]; effect size= 0.8845066. 4h Ctrl vs AMPKα shRNA: **p*＜0.05; [95% CI, -34.69 — -4.450]; effect size= 0.8262336. **N,** Day4 Ctrl vs AMPKα shRNA: ***p*＜0.01; [95% CI, -116.6 — -35.87]; effect size= 0.8656971. AMPKα shRNA vs AMPKα shRNA + FLAG-VHL: ***p*＜0.01; [95% CI, 20.87 — 101.6]; effect size= 0.8851296. Day5 Ctrl vs AMPKα shRNA: ****p*＜0.001; [95% CI, -253.6 — -101.4]; effect size= -0.9318683. AMPKα shRNA vs AMPKα shRNA + FLAG-VHL: ***p*＜0.01; [95% CI, 70.62 — 222.7]; effect size= 0.9152997. Day6 Ctrl vs AMPKα shRNA: ****p*＜0.001; [95% CI, -381.1 — -228.9]; effect size= -0.9838309. AMPKα shRNA vs AMPKα shRNA + FLAG-VHL: ****p*＜0.001; [95% CI, 144.5 — 296.8]; effect size= 0.958382. **O,** Sphere diameter: Ctrl vs AMPKα shRNA: ****p*＜0.001; [95% CI, -266.6 — -120.1]; effect size= -0.9519513. AMPKα shRNA vs AMPKα shRNA + FLAG-VHL: ***p*＜0.01; [95% CI, 40.06 — 186.6]; effect size= 0.9099239. No. of tumor spheres: Ctrl vs AMPKα shRNA: ***p*＜0.01; [95% CI, -56.53 — -20.13]; effect size= -0.9199693. AMPKα shRNA vs AMPKα shRNA + FLAG-VHL: ***p*＜0.01; [95% CI, 10.13 — 46.53]; effect size= 0.8663466. **P,** Ctrl vs AMPKα shRNA: ****p*＜0.001; [95% CI, -6.273 — -4.594]; effect size= -0.9955134. AMPKα shRNA vs AMPKα shRNA + FLAG-VHL: ****p*＜0.001; [95% CI, 2.861 — 4.539]; effect size= 0.9790113. **Q, Gemcitabine:** 1 μM Ctrl vs AMPKα shRNA: ***p*＜0.01; [95% CI, -30.53 — -9.470]; effect size= -0.9004557. AMPKα shRNA vs AMPKα shRNA + FLAG-VHL: **p*＜0.05; [95% CI, 4.803 — 25.86]; effect size= 0.9650743. 3 μM Ctrl vs AMPKα shRNA: ***p*＜0.01; [95% CI, -32.86 — -11.80]; effect size= -0.9178416. AMPKα shRNA vs AMPKα shRNA + FLAG-VHL: ***p*＜0.01; [95% CI, 7.470 — 28.53]; effect size= 0.9522037. 10 μM Ctrl vs AMPKα shRNA: ***p*＜0.01; [95% CI, -37.23 — -13.44]; effect size= -0.9162379. AMPKα shRNA vs AMPKα shRNA + FLAG-VHL: ***p*＜0.01; [95% CI, 9.769 — 33.56]; effect size= 0.9198304. 30 μM Ctrl vs AMPKα shRNA: ***p*＜0.01; [95% CI, -34.27 — -11.73]; effect size= -0.9075003. AMPKα shRNA vs AMPKα shRNA + FLAG-VHL: **p*＜0.05; [95% CI, 5.068 — 27.60]; effect size= 0.8762081. **Oxaliplatin:** 10 μM Ctrl vs AMPKα shRNA: ***p*＜0.01; [95% CI, -28.81 — -10.52]; effect size= -0.9947694. AMPKα shRNA vs AMPKα shRNA + FLAG-VHL: ***p*＜0.01; [95% CI, 4.852 — 23.15]; effect size= 0.9540539. 20 μM Ctrl vs AMPKα shRNA: ****p*＜0.001; [95% CI, -34.98 — -18.36]; effect size= -0.9682749. AMPKα shRNA vs AMPKα shRNA + FLAG-VHL: ****p*＜0.001; [95% CI, 12.36 — 28.98]; effect size= 0.9759084. 40 μM Ctrl vs AMPKα shRNA: ****p*＜0.001; [95% CI, -38.15 — -19.85]; effect size= -0.9622231. AMPKα shRNA vs AMPKα shRNA + FLAG-VHL: ***p*＜0.001; [95% CI, 13.19 — 31.48]; effect size= 0.9718412. 80 μM Ctrl vs AMPKα shRNA: ****p*＜0.001; [95% CI, -31.84 — -14.16]; effect size= -0.9509315. AMPKα shRNA vs AMPKα shRNA + FLAG-VHL: ***p*＜0.01; [95% CI, 10.50 — 28.17]; effect size= 0.9318323.

**Fig. 2. Identification of BAP1 as the *bona fide*** **deubiquitinase of pVHL. F,** PANC-1 Ctrl vs BAP1 shRNA#1: *p*＞0.05; [95% CI, -0.3022 — 0.08546]; effect size= -0.5541527. PANC-1 Ctrl vs BAP1 shRNA#2: *p*＞0.05; [95% CI, -0.3890 — -0.001408]; effect size= -0.7185983. BxPC3 Ctrl vs BAP1 shRNA#1: *p*＞0.05; [95% CI, -0.1131 — 0.3510]; effect size= 0.4901496. BxPC3 Ctrl vs BAP1 shRNA#2: *p*＞0.05; [95% CI, -0.1498 — 0.3143]; effect size= 0.3493177. ns, not significant. **H,** 1h Ctrl vs BAP1 shRNA#1: ****p*＜0.001; [95% CI, 30.51 — 63.81]; effect size= 0.9737167. 1h Ctrl vs BAP1 shRNA#2: ****p*＜0.001; [95% CI, 33.47 — 66.77]; effect size= 0.9573213. 2h Ctrl vs BAP1 shRNA#1: ***p*＜0.01; [95% CI, 12.88 — 48.99]; effect size= 0.8872874. Ctrl vs BAP1 shRNA#2: **p*＜0.05; [95% CI, 3.861 — 39.96]; effect size= 0.8037599. 4h Ctrl vs BAP1 shRNA#1: **p*＜0.05; [95% CI, 3.095 — 40.36]; effect size= 0.7704014. Ctrl vs BAP1 shRNA#2: **p*＜0.05; [95% CI, 2.692 — 39.96]; effect size= 0.7621402. **I,** Statistical significance was determined by one-way ANOVA followed by Tukey's multiple comparisons test. Vector vs FLAG-BAP1 WT: *p*＞0.05; [95% CI, -0.5467 — 0.4252]; effect size= -0.150985. Vector vs FLAG-BAP1 CS: *p*＞0.05; [95% CI, -0.7407 — 0.2312]; effect size= -0.5986276. ns, not significant. **J,** 2h Vector vs FLAG-BAP1 WT: ****p*＜0.001; [95% CI, -50.52 — -23.02]; effect size= -0.9567549. Vector vs FLAG-BAP1 CS: *p*＞0.05; [95% CI, -14.79 — 12.70]; effect size= -0.0829685. 4h Vector vs FLAG-BAP1 WT: ***p*＜0.01; [95% CI, -43.15 — -10.13]; effect size= -0.8751414. Vector vs FLAG-BAP1 CS: *p*＞0.05; [95% CI, -25.51 — 7.514]; effect size= -0.5975591.

**Fig. 3. BAP1 regulates PDAC progression through stabilizing pVHL. B,** PANC-1 Ctrl vs BAP1 shRNA#1: ****p*＜0.001; [95% CI, -2.749 — -0.9819]; effect size= -0.9226998. PANC-1 Ctrl vs BAP1 shRNA#2: ***p*＜0.01; [95% CI, -2.493 — -0.7263]; effect size= -0.9544014. PANC-1 BAP1 shRNA#2 vs BAP1 shRNA#2 + FLAG-VHL: ***p*＜0.01; [95% CI, 0.6703 — 2.437]; effect size= 0.9420244. BxPC3 Ctrl vs BAP1 shRNA#1: ****p*＜0.001; [95% CI, -2.894 — -1.490]; effect size= -0.8300253. BxPC3 Ctrl vs BAP1 shRNA#2: ****p*＜0.001; [95% CI, -2.388 — -0.9833]; effect size= -0.7464823. BxPC3 BAP1 shRNA#2 vs BAP1 shRNA#2 + FLAG-VHL: ***p*＜0.01; [95% CI, 0.8334 — 2.238]; effect size= 0.9282747. **C,** PANC-1 Day5 Ctrl vs BAP1 shRNA#1: ****p*＜0.001; [95% CI, -132.6 — -53.23]; effect size= -0.9402524. PANC-1 Ctrl vs BAP1 shRNA#2: ***p*＜0.01; [95% CI, -112.2 — -32.82]; effect size= -0.9353843. PANC-1 BAP1 shRNA#2 vs BAP1 shRNA#2 + FLAG-VHL: **p*＜0.05; [95% CI, 13.65 — 93.02]; effect size= 0.8946044. PANC-1 Day6 Ctrl vs. BAP1 shRNA#1: ****p*＜0.001（<0.0001）; [95% CI, -229.8 — -131.8]; effect size= -0.9722738. PANC-1 Ctrl vs BAP1 shRNA#2: ****p*＜0.001; [95% CI, -203.2 — -105.2]; effect size= -0.9910032. PANC-1 BAP1 shRNA#2 vs BAP1 shRNA#2 + FLAG-VHL: ****p*＜0.001; [95% CI, 64.34 — 162.3]; effect size= 0.9660883. BxPC3 Day5 Ctrl vs BAP1 shRNA#1: ***p*＜0.01; [95% CI, -150.8 — -29.20]; effect size= -0.8957613. BxPC3 Ctrl vs BAP1 shRNA#2: **p*＜0.05; [95% CI, -137.1 — -15.45]; effect size= -0.8414932. BxPC3 BAP1 shRNA#2 vs BAP1 shRNA#2 + FLAG-VHL: *p*＞0.05; [95% CI, -4.969 — 116.6]; effect size= 0.3829774. BxPC3 Day6 Ctrl vs BAP1 shRNA#1: ****p*＜0.001; [95% CI, -267.7 — -94.01]; effect size= -0.9248264. BxPC3 Ctrl vs BAP1 shRNA#2: ***p*＜0.01; [95% CI, -237.7 — -64.01]; effect size= -0.9359965. BxPC3 BAP1 shRNA#2 vs BAP1 shRNA#2+ FLAG-VHL: **p*＜0.05; [95% CI, 24.01 — 197.7]; effect size= 0.8880228. **D,** Sphere diameter: Ctrl vs BAP1 shRNA#1: ****p*＜0.001; [95% CI, -287.0 — -119.6]; effect size= -0.9452232. Ctrl vs BAP1 shRNA#2: ****p*＜0.001; [95% CI, -263.7 — -96.29]; effect size= -0.9340935. BAP1 shRNA#2 vs BAP1 shRNA#2 + FLAG-VHL: ***p*＜0.01; [95% CI, 49.62 — 217.0]; effect size=0.9190752. No. of tumor spheres: Ctrl vs BAP1 shRNA#1: ****p*＜0.001; [95% CI, -65.16 — -28.18]; effect size= -0.9638023. Ctrl vs BAP1 shRNA#2: ****p*＜0.001; [95% CI, -60.16 — -23.18]; effect size= -0.9552047. BAP1 shRNA#2 vs BAP1 shRNA#2 + FLAG-VHL: ***p*＜0.01; [95% CI, 9.845 — 46.82]; effect size= 0.8802636. **E,** Ctrl vs BAP1 shRNA#1: ****p*＜0.001; [95% CI, -5.514 — -3.020]; effect size= -0.9806237. Ctrl vs BAP1 shRNA#2: ****p*＜0.001; [95% CI, -5.347 — -2.853]; effect size= -0.9652197. BAP1 shRNA#2 vs BAP1 shRNA#2 + FLAG-VHL: ****p*＜0.001; [1.920 — 4.414]; effect size= 0.9495541. **F, Gemcitabine:** 1 μM Ctrl vs BAP1 shRNA#1: ***p*＜0.01; [95% CI, -34.05 — -9.287]; effect size= -0.8949848. Ctrl vs BAP1 shRNA#2: ****p*＜0.001; [95% CI, -38.05 — -13.29]; effect size= -0.9181821. BAP1 shRNA#2 vs BAP1 shRNA#2 + FLAG-VHL: ***p*＜0.01; [95% CI, 9.287 — 34.05]; effect size= 0.9392851. 3 μM Ctrl vs BAP1 shRNA#1: ****p*＜0.001; [95% CI, -40.13 — -19.87]; effect size= -0.9635757. Ctrl vs BAP1 shRNA#2: ****p*＜0.001; [95% CI, -37.46 — -17.21]; effect size= -0.9523183. BAP1 shRNA#2 vs BAP1 shRNA#2 + FLAG-VHL: ****p*＜0.001; [95% CI, 11.21 — 31.46]; effect size= 0.9485682. 10 μM Ctrl vs BAP1 shRNA#1: ****p*＜0.001; [95% CI, -45.34 — -19.33]; effect size= -0.9372439. 10 μM Ctrl vs BAP1 shRNA#1: ****p*＜0.001; [95% CI, -45.34 — -19.33]; effect size= -0.9372439. BAP1 shRNA#2 vs BAP1 shRNA#2 + FLAG-VHL: ****p*＜0.001; [95% CI, 13.99 — 40.01]; effect size= 0.9653107. 30 μM Ctrl vs BAP1 shRNA#1: ****p*＜0.001; [95% CI, -38.79 — -19.88]; effect size= -0.9582115. Ctrl vs BAP1 shRNA#2: ****p*＜0.001; [95% CI, --41.79 — -22.88]; effect size= -0.9704318. BAP1 shRNA#2 vs BAP1 shRNA#2 + FLAG-VHL: ****p*＜0.001; [95% CI, 14.54 — 33.46]; effect size= 0.9765419. Oxaliplatin: 10 μM Ctrl vs BAP1 shRNA#1: ****p*＜0.001; [95% CI, -29.57 — -11.77]; effect size= -0.9568853. Ctrl vs BAP1 shRNA#2: ***p*＜0.01; [95% CI, -25.57 — -7.768]; effect size= -0.9158786. BAP1 shRNA#2 vs BAP1 shRNA#2 + FLAG-VHL: ***p*＜0.01; [95% CI, 5.768 — 23.57]; effect size= 0.8950967. 20 μM Ctrl vs BAP1 shRNA#1: ****p*＜0.001; [95% CI, -32.70 — -15.30]; effect size= -0.9566844. Ctrl vs BAP1 shRNA#2: ****p*＜0.001; [95% CI, -28.70 — -11.30]; effect size= -0.9291415. BAP1 shRNA#2 vs BAP1 shRNA#2 + FLAG-VHL: ***p*＜0.01; [95% CI, 7.295 — 24.70]; effect size= 0.9373963. 40 μM Ctrl vs BAP1 shRNA#1: ***p*＜0.01; [95% CI, -38.70 — -12.64]; effect size= -0.9272793. Ctrl vs BAP1 shRNA#2: ***p*＜0.01; [95% CI, -34.03 — -7.970]; effect size= -0.8903371. BAP1 shRNA#2 vs BAP1 shRNA#2 + FLAG-VHL: ***p*＜0.01; [95% CI, 6.303 — 32.36]; effect size= 0.8965241. 80 μM Ctrl vs BAP1 shRNA#1: ***p*＜0.01; [95% CI, -37.64 — -11.02]; effect size= -0.9244674. Ctrl vs BAP1 shRNA#2: ***p*＜0.01; [95% CI, -35.64 — -9.022]; effect size= -0.9261648. BAP1 shRNA#2 vs BAP1 shRNA#2 + FLAG-VHL: ***p*＜0.01; [95% CI, 6.022 — 32.64]; effect size= 0.8820083. **G,** Ctrl vs BAP1 shRNA#1: ****p*＜0.001; [95% CI, -1.362 — -0.6176]; effect size= -0.6181362. Ctrl vs BAP1 shRNA#2: ****p*＜0.001; [95% CI, -1.271 — -0.5259]; effect size= -0.8786032. Ctrl vs Ctrl + Gemcitabine: ***p*＜0.01; [95% CI, 0.1309 — 0.8757]; effect size= 0.3771534. BAP1 shRNA#2 vs BAP1 shRNA#2 + FLAG-VHL: ****p*＜0.001; [95% CI, 0.2959 — 1.041]; effect size= 0.8498569. Ctrl + Gemcitabine vs BAP1 shRNA#1 + Gemcitabine: ****p*＜0.001; [95% CI, -1.109 — -0.3643]; effect size= -0.9074302. Ctrl+ Gemcitabine vs BAP1 shRNA#2 + Gemcitabine: ****p*＜0.001; [95% CI, -1.207 — -0.4626]; effect size= -0.976023. BAP1 shRNA#2 + Gemcitabine vs BAP1 shRNA#2 + FLAG-VHL + Gemcitabine: ****p*＜0.001; [95% CI, 0.3209 — 1.066]; effect size= 0.6755506. **H,** Ctrl vs BAP1 shRNA#1: ****p*＜0.001; [95% CI, -1.276 — -0.5411]; effect size= -0.8802593. Ctrl vs Ctrl + Gemcitabine: ***p*＜0.01; [95% CI, 0.1328 — 0.8672]; effect size= 0.7912326. BAP1 shRNA#1 vs BAP1 shRNA#1 + FLAG-VHL: ****p*＜0.001; [95% CI, 0.4244 — 1.159]; effect size= 0.8533509. Ctrl + Gemcitabine vs BAP1 shRNA#1 + Gemcitabine: ****p*＜0.001; [95% CI, -1.396 — -0.6611]; effect size= -0.8711559. BAP1 shRNA#1 + Gemcitabine vs BAP1 shRNA#1 + FLAG-VHL + Gemcitabine: ****p*＜0.001; [95% CI, 0.5644 — 1.299]; effect size= 0.9273443. **I,** Ctrl vs BAP1 shRNA#1: ***p*＜0.01; [95% CI, -35.16 — -6.502]; effect size= -0.7710573. Ctrl vs BAP1 shRNA#2: **p*＜0.05; [95% CI, -32.33 — -3.669]; effect size= -0.6780689. BAP1 shRNA#2 vs BAP1 shRNA#2 + FLAG-VHL: **p*＜0.05; [95% CI, 0.5022 — 29.16]; effect size= 0.6306615. **K,** Day4 Vector vs FLAG-BAP1 WT: ****p*＜0.001; [95% CI, 24.45 — 46.22]; effect size= 0.9512726. FLAG-BAP1 WT vs FLAG-BAP1 WT + VHL shRNA: ***p*＜0.01; [95% CI, -31.05 — -9.279]; effect size= -0.9288939. Day5 Vector vs FLAG-BAP1 WT: ****p*＜0.001; [95% CI, 36.57 — 87.93]; effect size= 0.9474951. FLAG-BAP1 WT vs FLAG-BAP1 WT + VHL shRNA: **p*＜0.05; [95% CI, -61.51 — -10.15]; effect size= -0.9015626. Day6 Vector vs FLAG-BAP1 WT: ****p*＜0.001; [95% CI, 146.0 — 224.6]; effect size= 0.9924304. FLAG-BAP1 WT vs FLAG-BAP1 WT + VHL shRNA: ****p*＜0.001; [95% CI, -146.8 — -68.21]; effect size= -0.9513453. **L,** Sphere diameter: Vector vs FLAG-BAP1 WT: ****p*＜0.001; [95% CI, 50.55 — 88.12]; effect size=0.9789573. FLAG-BAP1 WT vs FLAG-BAP1 WT + shVHL: ****p*＜0.001; [95% CI, -68.12 — -30.55]; effect size= -0.9718809. No. of tumor spheres: Vector vs FLAG-BAP1 WT: ****p*＜0.001; [95% CI, 11.12 — 15.54]; effect size=0.9925793. FLAG-BAP1 WT vs FLAG-BAP1 WT + VHL shRNA: ****p*＜0.001; [95% CI, -13.21 — -8.791]; effect size= -0.9838699. **M,** Vector vs FLAG-BAP1 WT: ****p*＜0.001; [95% CI, 0.5828 — 1.084]; effect size= 0.9636628. FLAG-BAP1 WT vs FLAG-BAP1 WT + VHL shRNA: ****p*＜0.001; [95% CI, -0.6505 — -0.1495]; effect size= -0.960757.

**Fig. 4. BAP1 N-Terminal domain and catalytic activity are critical for pVHL stabilization and tumor suppression in PDAC. D,** Day4 Vector vs FLAG-BAP1 WT: ***p*＜0.01; [95% CI, 7.047 — 24.62]; effect size= 0.8880282. Vector vs FLAG-BAP1 CS: *p*＞0.05; [95% CI, -17.45 — 0.1196]; effect size= -0.6978777. FLAG-BAP1 WT vs FLAG-BAP1^Δ1-240^: **p*＜0.05; [95% CI, -20.12 — -2.547]; effect size= -0.9591365. Day5 Vector vs FLAG-BAP1 WT: ****p*＜0.001; [95% CI, 23.58 — 61.42]; effect size= 0.9201491. Vector vs FLAG-BAP1 CS: *p*＞0.05; [95% CI, -40.17 — -2.333]; effect size= -0.8347993. FLAG-BAP1 WT vs FLAG-BAP1^Δ1-240^: ****p*＜0.001; [95% CI, -56.83 — -19.00]; effect size= -0.9304736. Day6 Vector vs FLAG-BAP1 WT: ****p*＜0.001; [95% CI, 94.57 — 153.8]; effect size= 0.9846735. Vector vs FLAG-BAP1 CS: *p*＞0.05; [95% CI, -32.93 — 26.26]; effect size= -0.1612318. FLAG-BAP1 WT vs FLAG-BAP1^Δ1-240^: ****p*＜0.001; [95% CI, -122.9 — -63.74]; effect size= -0.9676637. **E,** Gemcitabine: 1 μM Vector vs FLAG-BAP1 WT: ***p*＜0.01; [95% CI, 9.205 — 36.79]; effect size= 0.897318. Vector vs FLAG-BAP1 CS: *p*＞0.05; [95% CI, -12.46 — 15.13]; effect size= 0.1254313. FLAG-BAP1 WT vs FLAG-BAP1^Δ1-240^: ***p*＜0.01; [95% CI, -33.79 — -6.205]; effect size= -0.8838878. 3 μM Vector vs FLAG-BAP1 WT: ***p*＜0.01; [95% CI, 7.065 — 33.60]; effect size= 0.8591221. Vector vs FLAG-BAP1 CS: *p*＞0.05; [95% CI, -9.268 — 17.27]; effect size= 0.3573076. FLAG-BAP1 WT vs FLAG-BAP1^Δ1-240^: **p*＜0.05; [95% CI, -30.93 — -4.398]; effect size= -0.8738371. 10 μM Vector vs FLAG-BAP1 WT: ***p*＜0.01; [95% CI, 4.883 — 31.12]; effect size= 0.8504139. Vector vs FLAG-BAP1 CS: *p*＞0.05; [95% CI, -12.46 — 15.13]; effect size= 0.2181508. FLAG-BAP1 WT vs FLAG-BAP1^Δ1-240^: **p*＜0.05; [95% CI, -24.78 — 1.450]; effect size= -0.8360175. 30 μM Vector vs FLAG-BAP1 WT: ***p*＜0.01; [95% CI, 4.062 — 25.94]; effect size= 0.8500506. Vector vs FLAG-BAP1 CS: *p*＞0.05; [95% CI, -9.271 — 12.60]; effect size= 0.1676059. FLAG-BAP1 WT vs FLAG-BAP1^Δ1-240^: **p*＜0.05; [95% CI, -22.60 — -0.7286]; effect size= -0.8677061. Oxaliplatin**:** 10 μM Vector vs FLAG-BAP1 WT: ****p*＜0.001; [95% CI, 23.87 — 37.46]; effect size= 0.9891858. Vector vs FLAG-BAP1 CS: *p*＞0.05; [95% CI, -4.460 — 9.127]; effect size= 0.3591211. FLAG-BAP1 WT vs FLAG-BAP1^Δ1-240^: ****p*＜0.001; [95% CI, -32.46 — -18.87]; effect size= -0.9870868. 20 μM Vector vs FLAG-BAP1 WT: ****p*＜0.001; [95% CI, 17.28 — 36.72]; effect size= 0.8591221. Vector vs FLAG-BAP1 CS: *p*＞0.05; [95% CI, -5.058 — 14.39]; effect size= 0.3573076. FLAG-BAP1 WT vs FLAG-BAP1^Δ1-240^: ****p*＜0.001; [95% CI, -31.72 — -12.28]; effect size= -0.8738371. 40 μM Vector vs FLAG-BAP1 WT: ****p*＜0.001; [95% CI, 12.78 — 37.22]; effect size= 0.9086375. Vector vs FLAG-BAP1 CS: *p*＞0.05; [95% CI, -9.884 — 14.55]; effect size= 0.1940978. FLAG-BAP1 WT vs FLAG-BAP1^Δ1-240^: ***p*＜0.01; [95% CI, -34.22 — -9.782]; effect size= -0.9647638. 80 μM Vector vs FLAG-BAP1 WT: ****p*＜0.001; [95% CI, 8.897 — 26.44]; effect size= 0.9353048. Vector vs FLAG-BAP1 CS: *p*＞0.05; [95% CI, -9.103 — 8.437]; effect size= -0.0463076. FLAG-BAP1 WT vs FLAG-BAP1^Δ1-240^: ****p*＜0.001; [95% CI, -27.77 — -10.23]; effect size= -0.9495835. **I,** Day4 Vector vs FLAG-BAP1 WT: ****p*＜0.001; [95% CI, 17.42 — 50.58]; effect size= 0.9790518. FLAG-BAP1 WT vs FLAG-BAP1 M115L: ***p*＜0.01; [95% CI, -43.91 — -10.75]; effect size= -0.9635002. FLAG-BAP1 WT vs FLAG-BAP R179W: ****p*＜0.001; [95% CI, -49.58 — -16.42]; effect size= -0.9055827. Day5 Vector vs FLAG-BAP1 WT: ***p*＜0.01; [95% CI, 29.84 — 122.7]; effect size= 0.9254317. FLAG-BAP1 WT vs FLAG-BAP1 M115L: **p*＜0.05; [95% CI, -104.7 — -11.92]; effect size= -0.9014269. FLAG-BAP1 WT vs FLAG-BAP R179W: ***p*＜0.01; [95% CI, -123.5 — -30.67]; effect size= -0.932442. Day6 Vector vs FLAG-BAP1 WT: ****p*＜0.001; [95% CI, 81.85 — 238.1]; effect size= 0.930761. FLAG-BAP1 WT vs FLAG-BAP1 M115L: ****p*＜0.001; [95% CI, -247.3 — -91.02]; effect size= -0.956634. FLAG-BAP1 WT vs FLAG-BAP R179W: ***p*＜0.01; [95% CI, -201.5 — -45.19]; effect size= -0.9262723. **J,** Gemcitabine: 1 μM Vector vs FLAG-BAP1 WT: ***p*＜0.01; [95% CI, 7.522 — 29.81]; effect size= 0.925175. FLAG-BAP1 WT vs FLAG-BAP1 M115L: ****p*＜0.001; [95% CI, -49.81 — -27.52]; effect size= -0.9746879. FLAG-BAP1 WT vs FLAG-BAP R179W: ****p*＜0.001; [95% CI, -53.14 — -30.86]; effect size= -0.9877551. 3 μM Vector vs FLAG-BAP1 WT: **p*＜0.05; [95% CI, 2.671 — 35.33]; effect size= 0.9095978. FLAG-BAP1 WT vs FLAG-BAP1 M115L: ****p*＜0.001; [95% CI, -57.00 — -24.34]; effect size= -0.9605479. FLAG-BAP1 WT vs FLAG-BAP R179W: ****p*＜0.001; [95% CI, --58.33 — -25.67]; effect size= -0.9527411. 10 μM Vector vs FLAG-BAP1 WT: **p*＜0.05; [95% CI, 0.4944 — 31.51]; effect size= 0.8280873. FLAG-BAP1 WT vs FLAG-BAP1 M115L: ****p*＜0.001; [95% CI, -52.84 — -21.83]; effect size= -0.9612375. FLAG-BAP1 WT vs FLAG-BAP R179W: ****p*＜0.001; [95% CI, -54.84 — -23.83]; effect size= -0.9672075. 30 μM Vector vs FLAG-BAP1 WT: **p*＜0.05; [95% CI, 2.306 — 31.69]; effect size= 0.8972092. FLAG-BAP1 WT vs FLAG-BAP1 M115L: ****p*＜0.001; [95% CI, -47.69 — -18.31]; effect size= -0.9602592. FLAG-BAP1 WT vs FLAG-BAP R179W: ****p*＜0.001; [95% CI, -53.03 — -23.64]; effect size= -0.9619083. Oxaliplatin: 10 μM Vector vs FLAG-BAP1 WT: ****p*＜0.001; [95% CI, 21.38 — 37.29]; effect size= 0.9826364. FLAG-BAP1 WT vs FLAG-BAP1 M115L: ****p*＜0.001; [95% CI, -54.95 — -39.05]; effect size= -0.9929816. FLAG-BAP1 WT vs FLAG-BAP R179W: ****p*＜0.001; [95% CI, -54.62 — -38.71]; effect size= -0.9915336. 20 μM Vector vs FLAG-BAP1 WT: ****p*＜0.001; [95% CI, 14.87 — 35.79]; effect size= 0.9837861. FLAG-BAP1 WT vs FLAG-BAP1 M115L: ****p*＜0.001; [95% CI, -56.79 — -35.87]; effect size= -0.9772076. FLAG-BAP1 WT vs FLAG-BAP R179W: ****p*＜0.001; [95% CI, -57.13 — -36.21]; effect size= -0.9948357. 40 μM Vector vs FLAG-BAP1 WT: ****p*＜0.001; [95% CI, 12.78 — 37.22]; effect size= 0.9773219. FLAG-BAP1 WT vs FLAG-BAP1 M115L: ****p*＜0.001; [95% CI, -60.22 — -35.78]; effect size= -0.9690902. FLAG-BAP1 WT vs FLAG-BAP R179W: ****p*＜0.001; [95% CI, -61.22 — -36.78]; effect size= -0.9972337. 80 μM Vector vs FLAG-BAP1 WT: ****p*＜0.001; [95% CI, 9.710 — 24.96]; effect size= 0.9662871. FLAG-BAP1 WT vs FLAG-BAP1 M115L: ****p*＜0.001; [95% CI, -46.29 — -31.04]; effect size= -0.9860273. FLAG-BAP1 WT vs FLAG-BAP R179W: ****p*＜0.001; [95% CI, -48.96 — -33.71]; effect size= -0.9984422.

**Fig. 6. AMPKα-mediated phosphorylation of BAP1 regulates the stability and tumor-suppressive function of pVHL. D,** 1h Vector vs FLAG-BAP1 WT: ****p*＜0.001; [95% CI, -68.30 — -28.41]; effect size= -0.9412911. Vector vs FLAG-BAP1 3A: *p*＞0.05; [95% CI, -27.80 — 12.09]; effect size= -0.4169309. 2h Vector vs FLAG-BAP1 WT: **p*＜0.05; [95% CI, -47.51 — 0.3121]; effect size= -0.7290095. Vector vs FLAG-BAP1 3A: *p*＞0.05; [95% CI, -23.79 — 24.04]; effect size= 0.0099114. 4h Vector vs FLAG-BAP1 WT: **p*＜0.05; [95% CI, -36.99 — -6.376]; effect size= -0.8646044. Vector vs FLAG-BAP1 3A: *p*＞0.05; [95% CI, -16.50 — 14.11]; effect size= -0.1244947. **H,** Day5 FLAG-BAP1 WT vs FLAG-BAP1 WT + Metformin: *p*＞0.05; [95% CI, -13.65 — 73.81]; effect size= 0.7639796. Day5 FLAG-BAP1 WT vs FLAG-BAP1 3A: **p*＜0.05; [95% CI, -131.6 — -44.10]; effect size= -0.9198015. Day6 FLAG-BAP1 WT vs FLAG-BAP1 WT + Metformin: **p*＜0.05; [95% CI, 8.380 — 137.0]; effect size= 0.9014213. Day6 FLAG-BAP1 WT vs. FLAG-BAP1 3A: ****p*＜0.001; [95% CI, -231.0 — -102.4]; effect size= -0.9373623. **I,** Sphere diameter: FLAG-BAP1 WT vs FLAG-BAP1 WT + Metformin: ***p*＜0.01; [95% CI, 56.43 — 216.9]; effect size= 0.9443514. FLAG-BAP1 WT vs FLAG-BAP1 3A: ***p*＜0.01; [95% CI, -230.2 — -69.76]; effect size= -0.8958879. No. of tumor spheres: FLAG-BAP1 WT vs FLAG-BAP1 WT + Metformin: ***p*＜0.01; [95% CI, 8.651 — 51.35]; effect size=0.9486833. FLAG-BAP1 WT vs FLAG-BAP1 3A: ***p*＜0.01; [95% CI, -59.68 — -16.98]; effect size= -0.9476868. **J,** Gemcitabine: 1 μM FLAG-BAP1 WT vs FLAG-BAP1 WT + Metformin: ***p*＜0.01; [95% CI, 5.457 — 31.21]; effect size= 0.886829. FLAG-BAP1 WT vs FLAG-BAP1 3A: ***p*＜0.01; [95% CI, -34.21 — -8.457]; effect size= -0.9114728. 3 μM FLAG-BAP1 WT vs FLAG-BAP1 WT + Metformin: **p*＜0.05; [95% CI, 3.395 — 31.27]; effect size= 0.9250021. FLAG-BAP1 WT vs FLAG-BAP1 3A: ***p*＜0.01; [95% CI, -33.27 — -5.395]; effect size= -0.8907833. 10 μM FLAG-BAP1 WT vs FLAG-BAP1 WT + Metformin: **p*＜0.05; [95% CI, 3.023 — 27.64]; effect size= 0.8215484. FLAG-BAP1 WT vs FLAG-BAP1 3A: ***p*＜0.01; [95% CI, -32.64 — -8.023]; effect size= -0.9002539. 30 μM FLAG-BAP1 WT vs FLAG-BAP1 WT + Metformin: **p*＜0.05; [95% CI, 0.7964 — 27.20]; effect size= 0.7925939. BAP1 WT vs BAP1 3A: **p*＜0.05; [95% CI, -31.20 — -4.796]; effect size= -0.8181774. Oxaliplatin: 10 μM FLAG-BAP1 WT vs FLAG-BAP1 WT + Metformin: ****p*＜0.001; [95% CI, 15.13 — 36.21]; effect size= 0.9459911. FLAG-BAP1 WT vs FLAG-BAP1 3A: ***p*＜0.01; [95% CI, -27.87 — -6.793]; effect size= -0.9190069. 20 μM FLAG-BAP1 WT vs FLAG-BAP1 WT + Metformin: ***p*＜0.01; [95% CI, 7.439 — 37.89]; effect size= 0.978317. FLAG-BAP1 WT vs FLAG-BAP1 3A: ***p*＜0.01; [95% CI, -37.23 — -6.772]; effect size= -0.9198568. 40 μM FLAG-BAP1 WT vs FLAG-BAP1 WT + Metformin: ***p*＜0.01; [95% CI, 10.36 — 34.98]; effect size= 0.947381. FLAG-BAP1 WT vs FLAG-BAP1 3A: ***p*＜0.01; [95% CI, -34.64 — -10.02]; effect size= -0.9167458. 80 μM FLAG-BAP1 WT vs FLAG-BAP1 WT + Metformin: **p*＜0.05; [95% CI, 3.325 — 28.68]; effect size= 0.8944199. FLAG-BAP1 WT vs FLAG-BAP1 3A: ***p*＜0.01; [95% CI, -35.68 — -10.32]; effect size= -0.9000523. **K,** BAP1 WT vs BAP1 WT + Metformin: ***p*＜0.01; [95% CI, 0.1328 — 0.8072]; effect size= 0.8173872. BAP1 WT vs BAP1 3A: ****p*＜0.001; [95% CI, -1.224 — -0.5495]; effect size= -0.9081564. BAP1 WT vs BAP1 WT + Gemcitabine: ***p*＜0.01; [95% CI, 0.1445 — 0.8188]; effect size= 0.8013151. BAP1 WT + Gemcitabine vs BAP1 WT + Metformin + Gemcitabine: ****p*＜0.001; [95% CI, 0.2278 — 0.9022]; effect size= 0.8961787. BAP1 WT + Gemcitabine vs BAP1 3A + Gemcitabine: ****p*＜0.001; [95% CI, -1.024 — -0.3495]; effect size= -0.8853954. **L,** FLAG-BAP1 WT vs FLAG-BAP1 WT + Metformin: **p*＜0.05; [95% CI, 0.3077 — 24.69]; effect size= 0.7089029. FLAG-BAP1 WT vs FLAG-BAP1 3A: **p*＜0.05; [95% CI, -24.86 — -0.4744]; effect size= -0.5707936.

**Supplementary Figure 1.**


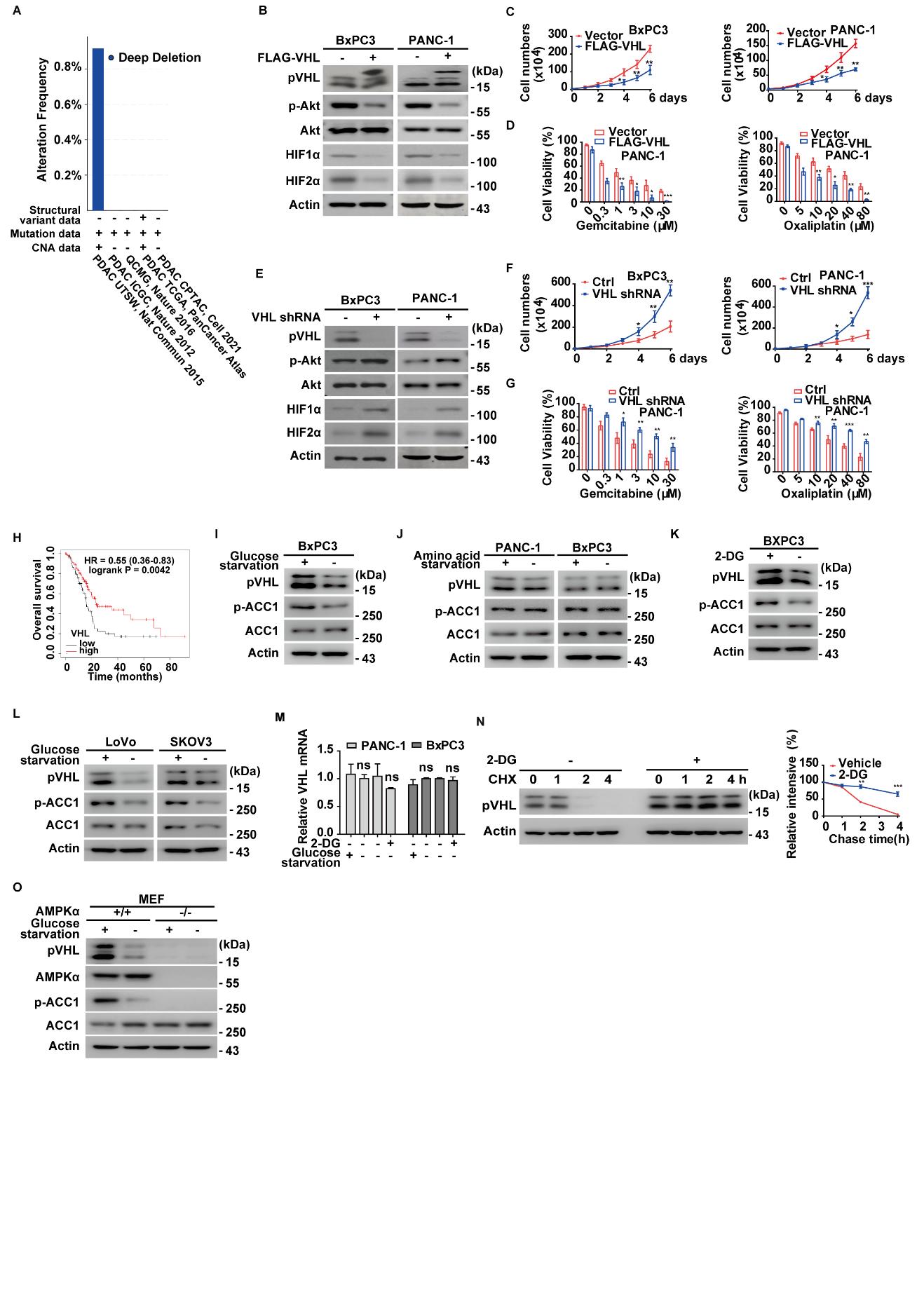
**Fig. S1. Glucose homeostasis regulates pVHL in an AMPK-dependent manner. (A)** Analysis of the status of VHL in PDAC by cBioPortal for Cancer Genomics databases. PDAC represents Pancreatic Ductal Adenocarcinoma. **(B)** BxPC3 and PANC-1 cells stably expressing vector or FLAG-VHL were generated, and western blot was performed with indicated antibodies. Data are representative of three independent experiments. **(C)** Cell proliferation assay was performed in BxPC3 and PANC-1 cells. Results represent the mean ± s.d. of three independent experiments (biological replicates). Statistical significance was determined by a two-tailed Student's t-test. BxPC3 Day4 Vector vs FLAG-VHL: **p*＜0.05; [95% CI, -95.17 — -16.50]; effect size= 0.8492592. BxPC3 Day5 Vector vs FLAG-VHL: ***p*＜0.01; [95% CI, -118.9 — -32.73]; effect size= 0.8939809. BxPC3 Day6 Vector vs FLAG-VHL: ***p*＜0.01;[95% CI, -173.2 — -66.84];effect size= 0.9313732. PANC-1 Day4 Vector vs FLAG-VHL: **p*＜0.05; [95% CI, -66.72 — -0.9445]; effect size= 0.7590278. PANC-1 Day5 Vector vs FLAG-VHL: ***p*＜0.01; [95% CI, -102.0 — -4.690]; effect size= 0.7790925. PANC-1 Day6 Vector vs FLAG-VHL: ***p*＜0.01; [95% CI, -127.1 — -46.87]; effect size= 0.9262023. **(D)** PANC-1 cells as in (B) were treated with indicated concentrations of gemcitabine or oxaliplatin, and cell viability was determined. Results represent the mean ± s.d. of three independent experiments (biological replicates). Statistical significance was determined by a two-tailed Student's t-test. Gemcitabine: 1 μM Vector vs FLAG-VHL: ***p*＜0.01; [95% CI, -36.66 — -9.335]; effect size= 0.8857087**.** 3 μM Vector vs FLAG-VHL: **p*＜0.05; [95% CI, -32.97 — -3.700]; effect size= 0.8175831. 10 μM Vector vs FLAG-VHL: **p*＜0.05; [95% CI, -36.32 — -5.015]; effect size= 0.8315134. 30 μM Vector vs FLAG-VHL: ****p*＜0.01; [95% CI, -21.14 — -12.86]; effect size= 0.9776865. Oxaliplatin: 10 μM Vector vs FLAG-VHL: ***p*＜0.01; [95% CI, -36.37 — -12.96]; effect size= 0.9224338. 20 μM Vector vs FLAG-VHL: **p*＜0.05; [95% CI, -36.85 — -14.48]; effect size= 0.9333957. 40 μM Vector vs FLAG-VHL: ***p*＜0.01; [95% CI, -32.64 — -12.03]; effect size= 0.9261621. 80 μM Vector vs FLAG-VHL: ***p*＜0.01; [95% CI, -28.08 — -12.59]; effect size= 0.9479127. **(E)** BxPC3 and PANC-1 cells stably expressing control or VHL shRNA were generated, and western blot was performed with indicated antibodies. Data are representative of three independent experiments. **(F)** Cell proliferation assay was performed in BxPC3 and PANC-1 cells. Results represent the mean ± s.d. of three independent experiments (biological replicates). Statistical significance was determined by a two-tailed Student's t-test. BxPC3 Day4 Ctrl vs VHL shRNA: **p*＜0.05; [95% CI, 18.79 — 147.0]; effect size= -0.8260519. BxPC3 Day5 Ctrl vs VHL shRNA: ***p*＜0.01; [95% CI, 68.29 — 265.0]; effect size= -0.8868688. BxPC3 Day6 Ctrl vs VHL shRNA: ***p*＜0.01; [95% CI, 218.8 — 447.9]; effect size= -0.9569679. PANC-1 Day4 Ctrl vs VHL shRNA: **p*＜0.05; [95% CI, 0.6188 — 138.4]; effect size= -0.7526795**.** PANC-1 Day5 Ctrl vs VHL shRNA: **p*＜0.05; [95% CI, 61.55 — 251.8]; effect size= -0.8815664. PANC-1 Day6 Ctrl vs VHL shRNA: ****p*＜0.001; [95% CI, 287.7 — 509.0]; effect size= 0.7158742. **(G)** PANC-1 cells as in (E) were treated with indicated concentrations of gemcitabine or oxaliplatin, and cell viability was determined. Results represent the mean ± s.d. of three independent experiments (biological replicates). Statistical significance was determined by a two-tailed Student's t-test. Gemcitabine: 1 μM Ctrl vs VHL shRNA: **p*＜0.05; [95% CI, 8.277 — 40.39]; effect size= -0.8641894**.** 3 μM Ctrl vs VHL shRNA: ***p*＜0.01; [95% CI, 10.19 — 32.48]; effect size= -0.985356. 10 μM Ctrl vs VHL shRNA: ***p*＜0.01; [95% CI, 17.16 — 36.84]; effect size= -0.9520209. 30 μM Ctrl vs pVHL shRNA: ***p*＜0.01; [95% CI, 8.678 — 33.99]; effect size= -0.8859574. Oxaliplatin: 10 μM Ctrl vs VHL shRNA: ***p*＜0.01; [95% CI, 4.684 — 15.32]; effect size= -0.9053477. 20 μM Ctrl vs VHL shRNA: ***p*＜0.01; [95% CI, 9.441 — 32.56]; effect size= -0.8995358. 40 μM Ctrl vs VHL shRNA: ****p*＜0.001; [95% CI, 18.06 — 30.61]; effect size= -0.9750544. 80 μM Ctrl vs VHL shRNA: ***p*＜0.01; [95% CI, 14.25 — 33.75]; effect size= -0.9413498. (**H)** Survival curve evaluating the prognostic value of pVHL in pancreatic cancer patients from Kaplan-Meier plots. *p*-value is shown in the graph. **(I)** BxPC3 cells were glucose starved (2 mM glucose) for 12 h, and western blot was performed with indicated antibodies. Data are representative of three independent experiments. **(J)** PANC-1 and BxPC3 cells were cultured with amino acid starvation medium for 12 h, and western blot was performed with indicated antibodies. Data are representative of three independent experiments. **(K)** BxPC3 cells were treated with 5 mM 2-DG for 24 h, and western blot was performed with indicated antibodies. Data are representative of three independent experiments. **(L)** LoVo and SKOV3 cells were glucose starved (2 mM glucose) for 12 h, and western blot was performed with indicated antibodies. Data are representative of three independent experiments. **(M)** Total RNA was isolated, and reverse transcribed into cDNA from PANC-1 and BxPC3 cells. The mRNA level of *VHL* was determined by quantitative PCR. The relative levels of mRNA were normalized to GAPDH transcript levels. Results represent the mean ± s.d. of three independent experiments (biological replicates). Statistical significance was determined by a two-tailed Student's t-test. PANC-1 Glucose vs Glucose stavation: *P*＞0.05; [95% CI, -0.6122 — 0.4576]; effect size= 0.1610506. BxPC3 Glucose vs Glucose stavation: *P*＞0.05; [95% CI, -0.1527 — 0.3695]; effect size= -0.4248142. PANC-1 Vehicle vs 2-DG: *P*＞0.05; [95% CI, -0.8446 — 0.3963]; effect size= 0.3794818. BxPC3 Vehicle vs 2-DG: *P*＞0.05; [95% CI, -0.8446 — 0.3963]; effect size= 0.3794818. ns, not significant. **(N)** Cycloheximide pulse-chase assay was performed in PANC-1 cells treated with vehicle or 2-DG. The relative level of pVHL to actin was measured by image J. Results represent the mean ± s.d. of three independent experiments (biological replicates). Statistical significance was determined by a two-tailed Student's t-test. 2h Vehicle vs 2-DG: ***p*＜0.01; [95% CI, 30.39 — 60.93]; effect size= -0.9591336. 4h Vehicle vs 2-DG: ****p*＜0.001; [95% CI, 41.84 — 78.48]; effect size= -0.965785. **(O)** AMPKα1/2 wild-type or AMPKα1/2 double knockout (AMPK DKO) mouse embryonic fibroblasts (MEFs) were cultured with glucose starvation (1 mM glucose) or normal DMEM for 12 h, and western blot was performed with indicated antibodies. Data are representative of three independent experiments.

**Supplementary Figure 2.**


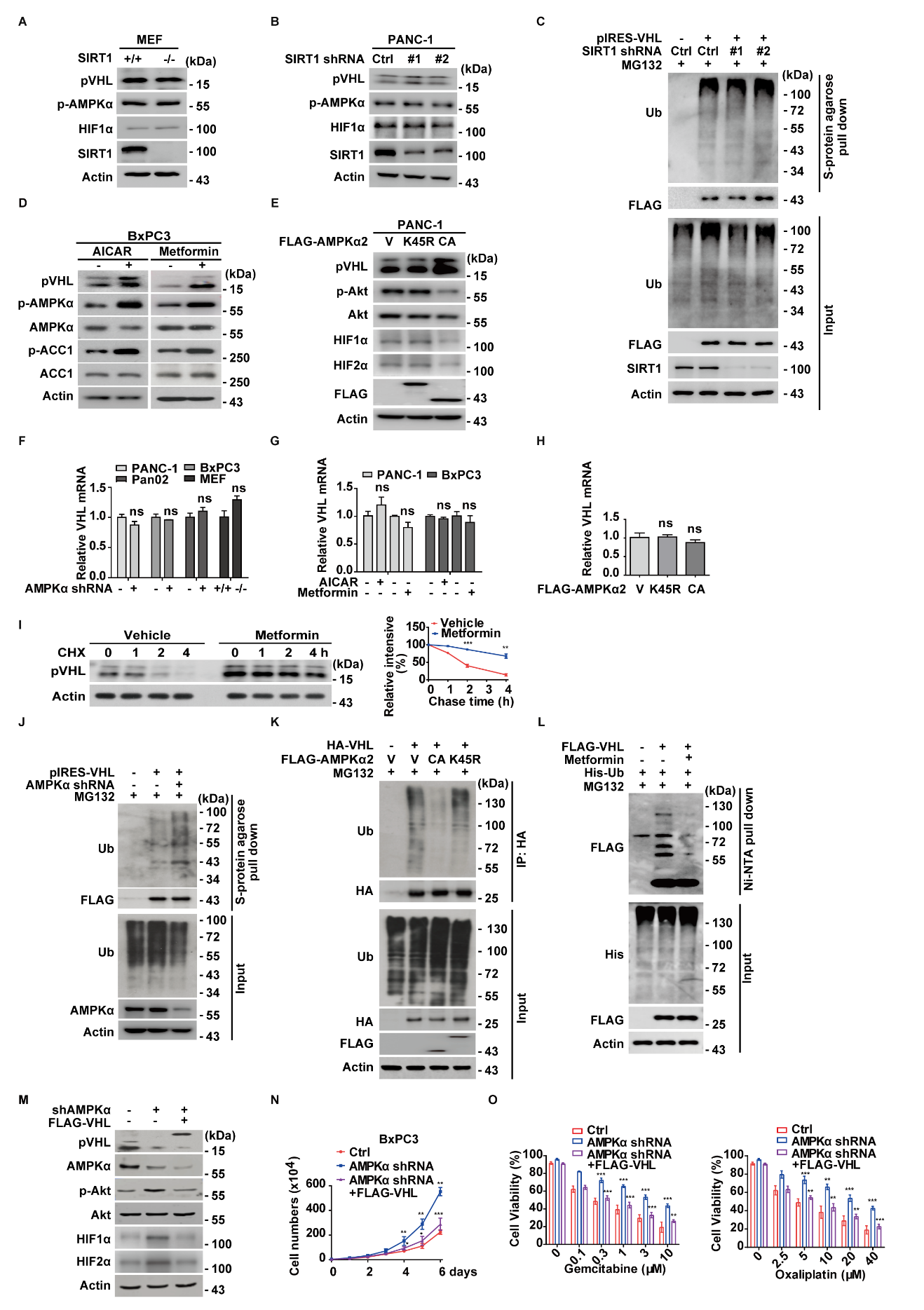


**Fig. S2.** **AMPKα regulates PDAC progression through stabilizing pVHL. (A)** SIRT1 wild-type (SIRT1^+/+^) or knockout (SIRT1^-/-^) mouse embryonic fibroblasts (MEFs) were cultured with DMEM, and western blotting was performed. Data are representative of three independent experiments. **(B)** PANC-1 cells stably expressing control or SIRT1 shRNAs (#1 and #2) were generated, and western blotting was performed. Data are representative of three independent experiments. **(C)** HEK293T cells stably expressing control or SIRT1 shRNAs were transfected with vector or pIRES-VHL, treated with MG132 (10 μM) for 10 h before harvest, and subjected to S-protein agarose pulldown. pVHL ubiquitination was assessed by western blotting with anti-ubiquitin antibody. Data are representative of three independent experiments. **(D)** BxPC3 cells were treated with AMPK activator AICAR (1 mM) for 8 h, or metformin (2 mM) for 24 h, respectively. Western blot was then performed with indicated antibodies. Data are representative of three independent experiments. **(E)** PANC-1 cells stably expressing vector, FLAG-AMPKα2 kinase dead mutant (K45R) or FLAG-AMPKα2 catalytically active mutant (CA), and western blot was performed with indicated antibodies. Data are representative of three independent experiments. **(F)** Total RNA was isolated, and reverse transcribed into cDNA from PANC-1, BxPC3, Pan02 murine pancreatic adenocarcinoma cells and MEFs. The mRNA level of *VHL* was determined by quantitative PCR. The relative levels of mRNA were normalized to GAPDH transcript levels. Results represent the mean ± s.d. of three independent experiments (biological replicates). Statistical significance was determined by a two-tailed Student's t-test. PANC-1 Ctrl vs AMPKα shRNA: *P*＞0.05; [95% CI, -0.3420 — 0.08510]; effect size= 0.5620472. BXPC3 Ctrl vs AMPKα shRNA: *P*＞0.05; [95% CI, -0.1897 — 0.1037]; effect size= 0.3173081. mPan02 Ctrl vs AMPKα shRNA: *P*＞0.05; [95% CI, -0.1528 — 0.3523]; effect size= -0.4060612. MEF AMPKα^+/+^ vs AMPKα^-/-^: *P*＞0.05; [95% CI, -0.04886 — 0.6166]; effect size= -0.6951862. ns, not significant. **(G)** Total RNA was isolated, and reverse transcribed into cDNA from PANC-1 and BxPC3 cells treated with vehicle, AICAR or metformin. The mRNA level of *VHL* was determined by quantitative PCR. The relative levels of mRNA were normalized to GAPDH transcript levels. Results represent the mean ± s.d. of three independent experiments (biological replicates). Statistical significance was determined by a two-tailed Student's t-test. PANC-1 Vehicle vs AICAR: *P*＞0.05; [95% CI, -0.2610 — 0.6535]; effect size= -0.4370139. BxPC3 Vehicle vs AICAR: *P*＞0.05; [95% CI, -0.1553 — 0.06121]; effect size= 0.4445005. PANC-1 Vehicle vs Metformin: *P*＞0.05; [95% CI, -0.4623 — 0.05811]; effect size= 0.6602437. BxPC3 Vehicle vs Metformin: *P*＞0.05; [95% CI, -0.5168 — 0.2835]; effect size= 0.3136317. ns, not significant. **(H)** Total RNA was isolated, and reverse transcribed into cDNA from PANC-1 cells stably expressing vector, FLAG-AMPKα2 K45R or FLAG-AMPKα2 CA. The mRNA level of *VHL* was determined by quantitative PCR. The relative levels of mRNA were normalized to GAPDH transcript levels. Results represent the mean ± s.d. of three independent experiments (biological replicates). Statistical significance was determined by one-way ANOVA followed by Tukey's multiple comparisons test. Vector vs FLAG-AMPKα K45R: *P*＞0.05; [95% CI, -0.3919 — 0.3649]; effect size= -0.0431003. Vector vs FLAG-AMPKα CA: *P*＞0.05; [95% CI, -0.2406 — 0.5161]; effect size= 0.3740063. ns, not significant. **(I)** Cycloheximide pulse-chase assay was performed in PANC-1 cells treated with metformin (1 mM) for 24 h. The relative level of pVHL to actin was measured by image J. Results represent the mean ± s.d. of three independent experiments (biological replicates). Statistical significance was determined by a two-tailed Student's t-test. 2h Vehicle vs Metformin: ****p*＜0.001; [95% CI, 33.79 — 58.90]; effect size= -0.9726042. 4h Vehicle vs Metformin: ***p*＜0.01; [95% CI, 34.55 — 72.56]; effect size= -0.9543444. **(J)** HEK293T cells stably expressing control or AMPKα shRNA were transfected with vector or pIRES-VHL (containing FLAG and S tag), and treated with MG132 (10 μM) for 10 h before harvest. Cell lysates were pull-downed by S-protein agarose and the ubiquitination of pVHL was measured by western blot with anti-ubiquitin antibody. Data are representative of three independent experiments. **(K)** HEK293T cells stably expressing vector, FLAG-AMPKα2 CA or FLAG-AMPKα2 K45R were transfected with indicated plasmids and treated with MG132 (10 μM) for 10 h before harvest. Cell lysates were subjected to immunoprecipitation with anti-HA magnetic beads and the ubiquitination of pVHL was measured by western blot with anti-ubiquitin antibody. Data are representative of three independent experiments. **(L)** Cells were cotransfected with indicated plasmids and Ni-NTA bead was used to pull down His-tagged ubiquitin, and the ubiquitination of pVHL was measured by western blot. Data are representative of three independent experiments. **(M)** BxPC3 cells stably expressing AMPKα shRNA were transfected with vector or FLAG-VHL. Western blot was performed with indicated antibodies. Data are representative of three independent experiments. **(N)** Cell proliferation assay was performed in BxPC3 cells. Results represent the mean ± s.d. of three independent experiments (biological replicates). Statistical significance was determined by one-way ANOVA followed by Tukey's multiple comparisons test. Day4 Ctrl vs AMPKα shRNA: ***p*＜0.01; [95% CI, -136.1 — -28.04]; effect size= -0.8551381**.** AMPKα shRNA vs AMPKα shRNA + FLAG-VHL: **p*＜0.05; [95% CI, 4.280 — 112.4]; effect size= 0.7449378. Day5 Ctrl vs AMPKα shRNA: ***p*＜0.01; [95% CI, -260.8 — -91.87]; effect size= -0.938063. AMPKα shRNA vs AMPKα shRNA + FLAG-VHL: **p*＜0.05; [95% CI, 49.70 — 218.6]; effect size= 0.8651076. Day6 Ctrl vs AMPKα shRNA：***p*＜0.01; [95% CI, -417.0 — -238.0]; effect size= -0.987047. AMPKα shRNA vs AMPKα shRNA + FLAG-VHL: ****p*＜0.001; [95% CI, 174.4 — 353.3]; effect size= 0.987178. **(O)** Cells as in (M) were treated with indicated concentrations of gemcitabine or oxaliplatin, and cell viability was determined. Results represent the mean ± s.d. of three independent experiments (biological replicates). Statistical significance was determined by one-way ANOVA followed by Tukey's multiple comparisons test. **Gemcitabine:** 0.3 μM Ctrl vs AMPKα shRNA: ****p*＜0.001; [95% CI, -30.90 — -16.43]; effect size= -0.9961913. AMPKα shRNA vs AMPKα shRNA + FLAG-VHL: ****p*＜0.001; [95% CI, 12.77 — 27.23]; effect size= 0.9697534. 1 μM Ctrl vs AMPKα shRNA: ****p*＜0.001; [95% CI, -35.13 — -17.54]; effect size= -0.9932322. AMPKα shRNA vs AMPKα shRNA + FLAG-VH: ****p*＜0.001; [95% CI, 12.54 — 30.13]; effect size= 0.9732857. 3 μM Ctrl vs AMPKα shRNA: ****p*＜0.001; [95% CI, -31.54 — -15.79]; effect size= -0.9650041. AMPKα shRNA vs AMPKα shRNA + FLAG-VHL: ****p*＜0.001; [95% CI, 12.46 — 28.21]; effect size= 0.9648414. 10 μM Ctrl vs AMPKα shRNA: ****p*＜0.001; [95% CI, -33.59 — -15.07]; effect size= -0.9405041. AMPKα shRNA vs AMPKα shRNA + FLAG-VHL: ***p*＜0.01; [95% CI, 8.072 — 26.59]; effect size= 0.97853. Oxaliplatin: 5 μM Ctrl vs AMPKα shRNA: ****p*＜0.001; [95% CI, -33.43 — -15.91]; effect size= -0.9507691. AMPKα shRNA vs AMPKα shRNA + FLAG-VHL: ***p*＜0.01; [95% CI, 10.57 — 28.09]; effect size= 0.9489467. 10 μM Ctrl vs AMPKα shRNA: ***p*＜0.01; [95% CI, -39.91 — -15.42]; effect size= -0.9347229. AMPKα shRNA vs AMPKα shRNA + FLAG-VHL: ***p*＜0.01; [95% CI, 10.09 — 34.58]; effect size= 0.9527711. 20 μM Ctrl vs AMPKα shRNA: ****p*＜0.001; [95% CI, -34.55 — -14.79]; effect size= -0.939656. AMPKα shRNA vs AMPKα shRNA + FLAG-VHL: ***p*＜0.01; [95% CI, 10.12 — 29.88]; effect size= 0.956359. 40 μM Ctrl vs AMPKα shRNA: ****p*＜0.001; [95% CI, -31.81 — -15.53]; effect size= -0.9576279. AMPKα shRNA vs AMPKα shRNA + FLAG-VHL: ****p*＜0.001; [95% CI, 11.86 — 28.14]; effect size= 0.974347.

**Supplementary Figure 3.**


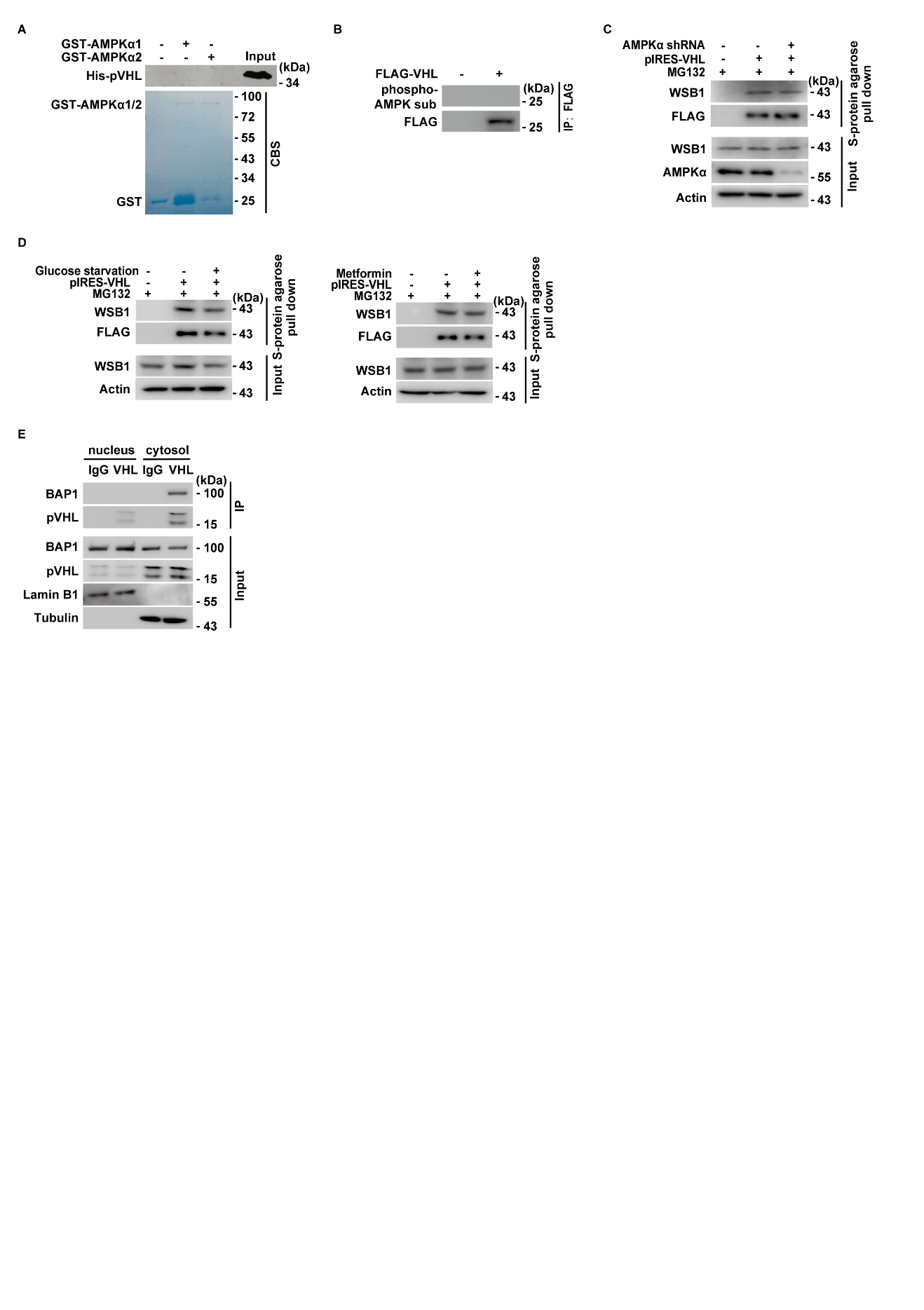


**Fig. S3. BAP1 interacts with pVHL.** **(A)** Purified recombinant GST, GST-AMPKα1, GST-AMPKα2 and His-pVHL were incubated *in vitro* as indicated. The interaction between pVHL and AMPKα1 or AMPKα2 was examined. CBS, Coomassie blue staining. Data are representative of three independent experiments. **(B)** Vector or FLAG-VHL were transfected in cells. Cell lysates were subjected to immunoprecipitation with anti-FLAG antibody and the phosphorylation of pVHL were examined by using phospho-AMPK substrate antibody. Data are representative of three independent experiments. **(C and D)** Cells were transfected with indicated plasmids and cell lysates were pull-downed by S-protein agaroses and western blot was performed. Data are representative of three independent experiments. **(E)** Subcellular fractionation and co-immunoprecipitation analysis of pVHL in PANC-1 cells. Nuclear and cytoplasmic fractions were prepared using a commercial fractionation kit. Co-immunoprecipitation was performed from each fraction using either control IgG or anti-pVHL antibody, followed by immunoblotting with the indicated antibodies. Data are representative of three independent experiments.

**Supplementary Figure 4.**


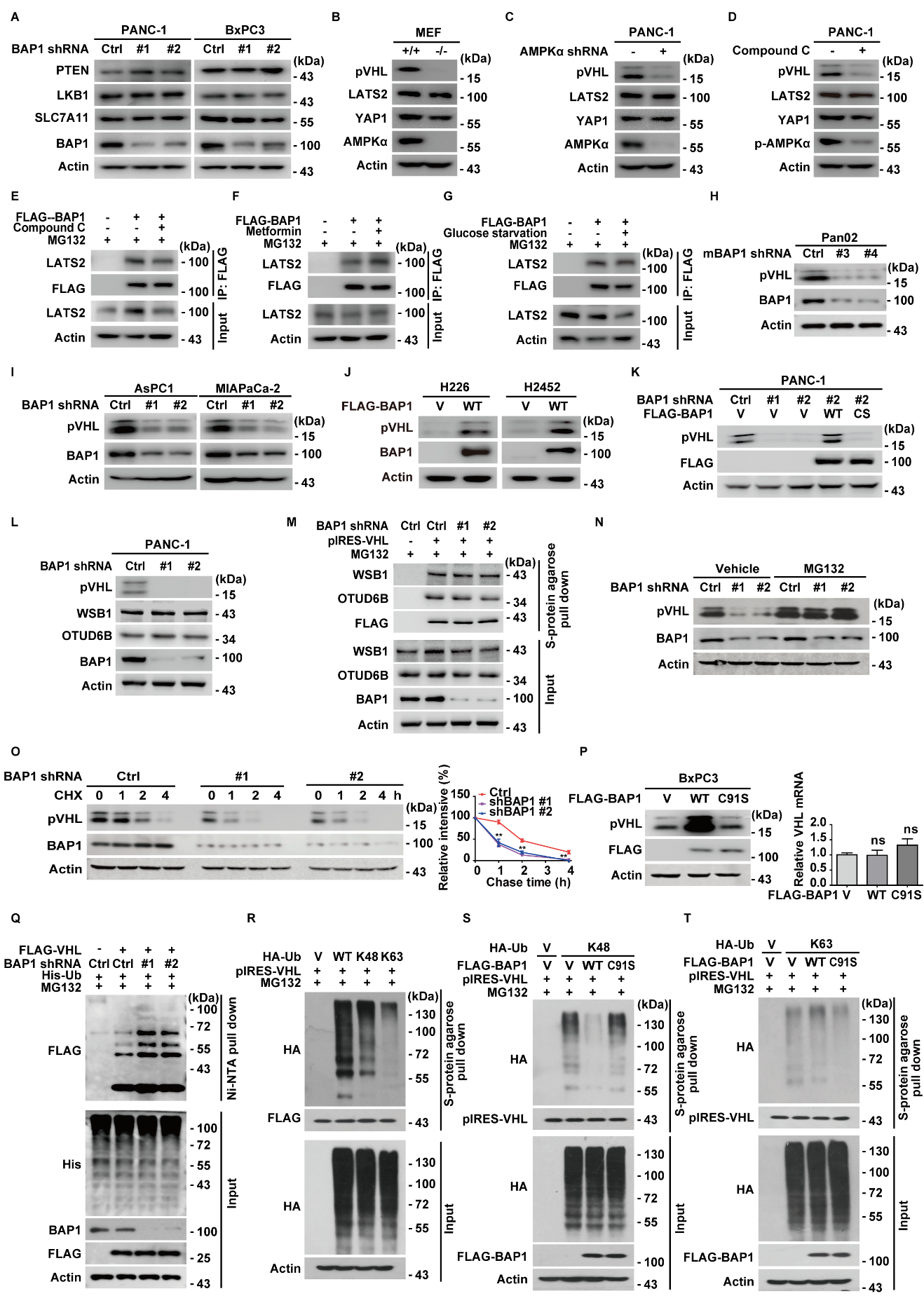


**Fig. S4. Identification of BAP1 as the *bona fide*** **deubiquitinase of pVHL.** **(A)** PANC-1 and BxPC3 cells stably expressing control or BAP1 shRNAs (#1 and #2) were generated, and western blotting was performed using the indicated antibodies. Data are representative of three independent experiments. **(B)** AMPKα1/2 wild type or AMPKα1/2 double knockout (AMPK DKO) MEFs cells were generated, and western blot was performed with indicated antibodies. Data are representative of three independent experiments. **(C)** PANC-1 cells stably expressing control or AMPKα shRNA were generated, and western blot was performed with indicated antibodies. Data are representative of three independent experiments. **(D)** PANC-1 cells were treated with vehicle or Compound C (5 μM) for 4 h. Western blot was performed with indicated antibodies. Data are representative of three independent experiments. **(E)** PANC-1 cells stably expressing vector or FLAG-BAP1 was treated with vehicle or Compound C (5 μM) for 4 h, and treated with MG132 (10 μM) for 10 h before harvest. Cell lysates were subjected to immunoprecipitation with anti-FLAG antibody and western blot was performed. Data are representative of three independent experiments. **(F)** PANC-1 cells stably expressing vector or FLAG-BAP1 was treated with vehicle or metformin (1 mM) for 24 h, and treated with MG132 (10 μM) for 10 h before harvest. Cell lysates were subjected to immunoprecipitation with anti-FLAG antibody and western blot was performed. Data are representative of three independent experiments. **(G)** PANC-1 cells stably expressing vector or FLAG-BAP1 was glucose starved (2 mM glucose) for 12 h, and treated with MG132 (10 μM) for 10 h before harvest. Cell lysates were subjected to immunoprecipitation with anti-FLAG antibody and western blot was performed. Data are representative of three independent experiments. **(H)** Pan02 murine pancreatic adenocarcinoma cells stably expressing control or BAP1 shRNAs (#3 and #4) were generated and western blot was performed with the indicated antibodies. Data are representative of three independent experiments. **(I)** AsPC1 and MIAPaCa-2 cells stably expressing control or BAP1 shRNAs (#1 and #2) were generated, and western blot was performed with indicated antibodies. Data are representative of three independent experiments. **(J)** H226 and H2452 cells stably expressing vector or FLAG-BAP1 WT were generated, and western blot was performed with indicated antibodies. Data are representative of three independent experiments. **(K)** PANC-1 cells stably expressing control or BAP1 shRNAs (#1 and #2) were transfected with vector, shRNA#2-resistant BAP1 wild-type (WT), or the catalytically inactive C91S mutant. Western blotting was performed with the indicated antibodies. Data are representative of three independent experiments. **(L)** PANC-1 cells stably expressing control or BAP1 shRNAs (#1 and #2) were generated, and western blotting was performed with the indicated antibodies. Data are representative of three independent experiments. **(M)** Cells were transfected as indicated, and cell lysates were subjected to S-protein agarose pulldown. Western blotting was performed with the indicated antibodies. Data are representative results from three independent experiments. **(N)** BxPC3 cells stably expressing control or BAP1 shRNAs were treated with vehicle or MG132 (10 μM) for 10 h before harvest, and western blot was performed with indicated antibodies. Data are representative of three independent experiments. **(O)** Cycloheximide pulse-chase assay was performed in BxPC3 cells. The relative level of pVHL to actin was measured by image J. Results represent the mean ± s.d. of three independent experiments (biological replicates). Statistical significance was determined by one-way ANOVA followed by Tukey's multiple comparisons test. 1h Ctrl vs BAP1 shRNA#1: ***p*＜0.01; [95% CI, 25.21 — 79.50]; effect size= 0.9506296. 1h Ctrl vs BAP1 shRNA#2: ***p*＜0.01; [95% CI, 21.04 — 75.33]; effect size= 0.9035457. 2h Ctrl vs BAP1 shRNA#1: ****p*＜0.001; [95% CI, 21.89 — 46.04]; effect size= 0.9668586. Ctrl vs BAP1 shRNA#2: ***p*＜0.01; [95% CI, 15.61 — 39.76]; effect size= 0.9248729. 4h Ctrl vs BAP1 shRNA#1: ***p*＜0.01; [95% CI, 6.013 — 28.86]; effect size= 0.8420548. Ctrl vs BAP1 shRNA#2: ***p*＜0.01; [95% CI, 8.346 — 31.19]; effect size= 0.8996104. **(P)** BxPC3 cells stably expressing vector, FLAG-BAP1 WT or BAP1 C91S mutant were generated, and western blot was performed with indicated antibodies. Total RNA was isolated, and reverse transcribed into cDNA from BxPC3 cells. The mRNA level of *VHL* was determined by quantitative PCR. The relative levels of mRNA were normalized to GAPDH transcript levels. Results represent the mean ± s.d. of three independent experiments (biological replicates). Statistical significance was determined by one-way ANOVA followed by Tukey's multiple comparisons test. Vector vs FLAG-BAP1 WT: *P*＞0.05; [95% CI, -0.6803 — 0.7189]; effect size= 0.0140421. Vector vs FLAG-BAP1 CS: *P*＞0.05; [95% CI, -1.015 — 0.3839]; effect size= -0.5016219. ns, not significant. **(Q)** Cells were cotransfected with indicated plasmids and Ni-NTA bead was used to pull down His-tagged ubiquitin, and the ubiquitination of pVHL was measured by western blot. Data are representative of three independent experiments. **(R)** Identify the type of ubiquitin linkage on pVHL. pIRES-VHL (containing FLAG and S tag), HA-ubiquitin WT and different mutants were co-transfected. Cell lysates were pull-downed by S-protein agaroses and immunoblotted as indicated. Data are representative of three independent experiments. **(S and T)** BAP1 WT, not the C91S mutant, decreases K48 ubiquitination of pVHL (L), but has no effect on pVHL K63 ubiquitination (M). Cells were transfected with indicated plasmids. Cell lysates were pull-downed by S-protein agaroses and immunoblotted as indicated. Data are representative of three independent experiments.

**Supplementary Figure 5.**


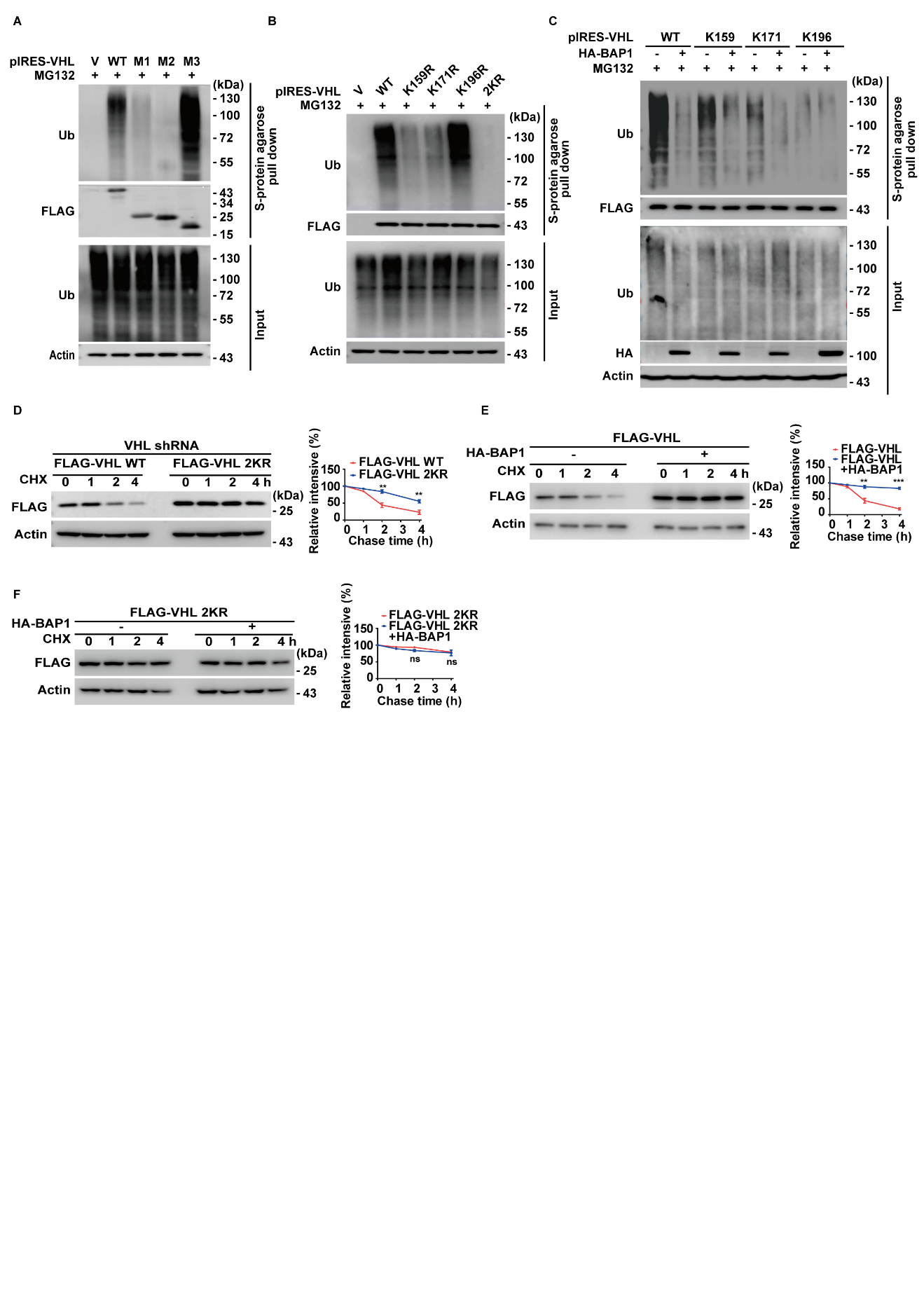


**Fig. S5. BAP1 mediates deubiquitination and stabilization of pVHL through targeting lysine residues K159 and K171.** **(A)** Full length pIRES-VHL or its truncation mutants were transfected with indicated plasmids in HEK293T cells and treated with MG132 (10 μM) for 10 h before harvest. Cell lysates were pull-downed by S-protein agaroses and immunoblotted as indicated. M1 (1-62 amino acids); M2 (63-156 amino acids); M3 (157-213 amino acids). Data are representative of three independent experiments. **(B)** Vector, pIRES-VHL WT or mutants were cotransfected with indicated plasmids in HEK293T cells and treated with MG132 (10 μM) for 10 h before harvest. Cell lysates were pull-downed by S-protein agaroses and the ubiquitination of pVHL was measured by western blot with anti-ubiquitin antibody. 2KR, K159/K171R. Data are representative of three independent experiments. **(C)** Vector, pIRES-VHL WT, K159, K171 or K196 were cotransfected with indicated plasmids in HEK293T cells and treated with MG132 (10 μM) for 10 h before harvest. Cell lysates were pull-downed by S-protein agaroses and the ubiquitination of pVHL was measured by western blot with anti-ubiquitin antibody. Data are representative of three independent experiments. **(D)** PANC-1 cells stably expressing *VHL* shRNA were transfected with FLAG-VHL or the 2KR mutant (K159 and K171 were replaced by arginine), and cycloheximide pulse-chase assay was performed. The relative level of FLAG-VHL to actin was measured by image J. Results represent the mean ± s.d. of three independent experiments (biological replicates). Statistical significance was determined by a two-tailed Student's t-test. 2h FLAG-VHL WT vs FLAG-VHL 2KR: ***p*＜0.01; [95% CI, 16.58 — 63.60]; effect size= -0.8881681. 4h FLAG-VHL WT vs FLAG-VHL 2KR: ***p*＜0.01; [95% CI, 33.26 — 71.68]; effect size= -0.9516105. (**E and F)** PANC-1 cells stably expressing FLAG-VHL (E) or the 2KR mutant (F) were transfected with vector or pCMV-HA-BAP1, and cycloheximide pulse-chase assay was performed. The relative level of FLAG-VHL to actin was measured by image J. Results represent the mean ± s.d. of three independent experiments (biological replicates). Statistical significance was determined by a two-tailed Student's t-test. 2h FLAG-VHL WT vs FLAG-VHL WT + HA-BAP1: ***p*＜0.01; [95% CI, 20.03 — 67.67]; effect size= -0.9018175. 4h FLAG-VHL WT vs FLAG-VHL WT + HA-BAP1: ****p*＜0.001; [95% CI, 51.28 — 78.06]; effect size= -0.9837306. 2h FLAG-VHL 2KR vs FLAG-VHL 2KR + HA-BAP1: *P*＞0.05; [95% CI, -18.97 — -0.4562]; effect size= 0.6655647. 4h FLAG-VHL 2KR vs FLAG-VHL 2KR + HA-BAP1: *P*＞0.05; [95% CI, -26.94 — 22.13]; effect size= 0.1106156. ns, not significant.

**Supplementary Figure 6.**


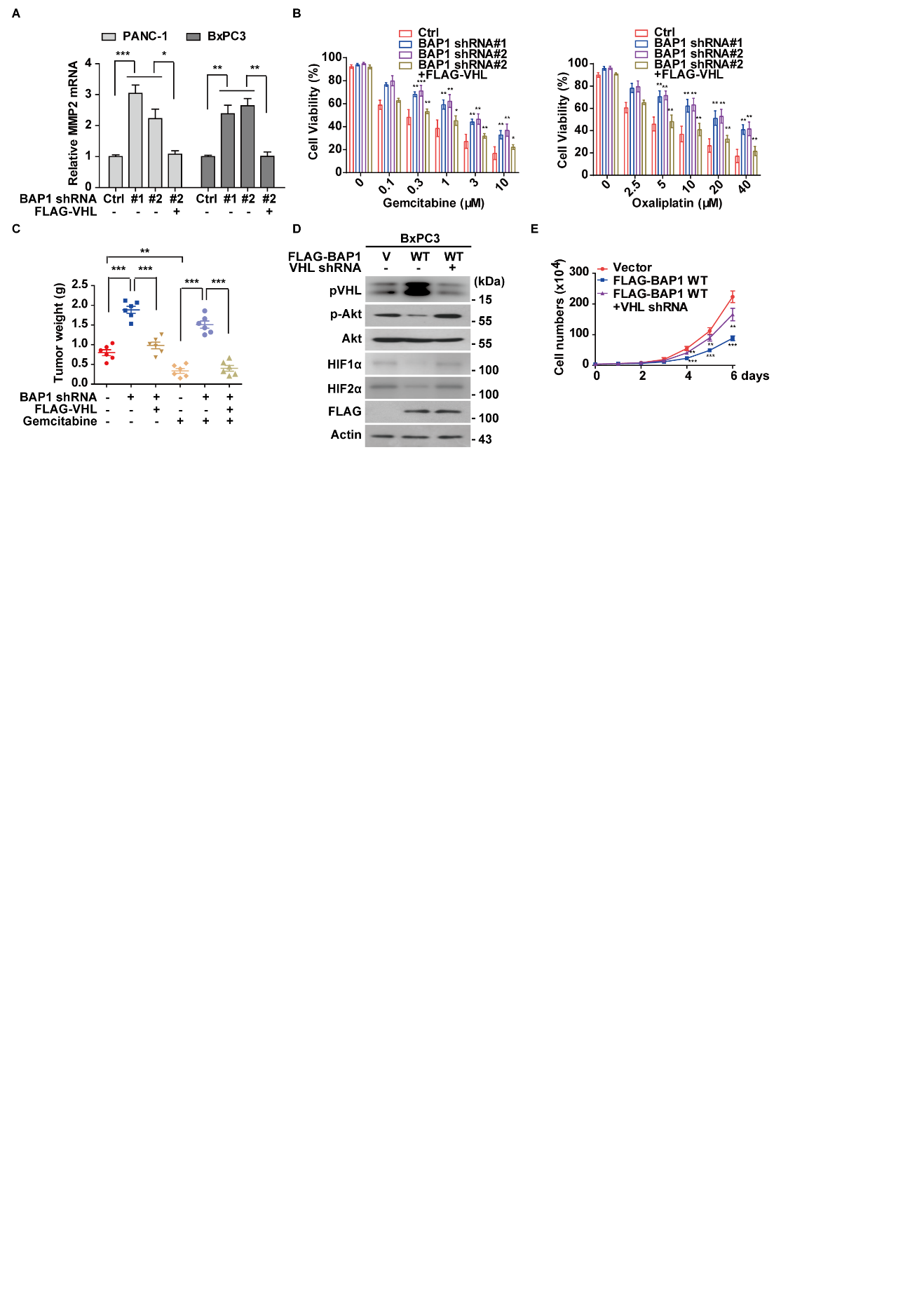


**Fig. S6. BAP1 regulates PDAC progression through stabilizing pVHL. (A)** Total RNA was isolated. Relative expression of *MMP2* of cells were determined by quantitative PCR. Results represent the mean ± s.d. of three independent experiments (biological replicates). Statistical significance was determined by one-way ANOVA followed by Tukey's multiple comparisons test. PANC-1 Ctrl vs BAP1 shRNA#1: ****p*＜0.001; [95% CI, -3.008 — -1.067]; effect size= -0.9728584. PANC-1 Ctrl vs BAP1 shRNA#2: **p*＜0.05; [95% CI, -2.193 — -0.2517]; effect size= -0.9358875. PANC-1 BAP1 shRNA#2 vs BAP1 shRNA#2 + FLAG-VHL: **p*＜0.05; [95% CI, 0.1765 — 2.118]; effect size= 0.8193487. BxPC3 Ctrl vs BAP1 shRNA#1: ***p*＜0.01; [95% CI, -2.259 — -0.5038]; effect size= -0.8974012. BxPC3 Ctrl vs BAP1 shRNA#2: ***p*＜0.01; [95% CI, -2.519 — -0.7632]; effect size= -0.9431227. BxPC3 BAP1 shRNA#2 vs BAP1 shRNA#2 + FLAG-VHL: ***p*＜0.01; [95% CI, 0.7573 — 2.513]; effect size= 0.9265932. **(B)** BxPC3 cells were treated with indicated concentrations of gemcitabine or oxaliplatin, and cell viability was determined. Results represent the mean ± s.d. of three independent experiments (biological replicates). Statistical significance was determined by one-way ANOVA followed by Tukey's multiple comparisons test. Gemcitabine: 0.3 μM Ctrl vs BAP1 shRNA#1: ***p*＜0.01; [95% CI, -31.20 — -8.804]; effect size= -0.9004575. Ctrl vs BAP1 shRNA#2: ****p*＜0.001; [95% CI, -34.20 — -11.80]; effect size= -0.8964112. BAP1 shRNA#2 vs BAP1 shRNA#2 + FLAG-VHL: ***p*＜0.01; [95% CI, 6.804 — 29.20]; effect size= 0.9940417. 1 μM Ctrl vs BAP1 shRNA#1: ***p*＜0.01; [95% CI, -34.77 — -6.566]; effect size= -0.8698223. Ctrl vs BAP1 shRNA#2: ***p*＜0.01; [95% CI, -37.77 — -9.566]; effect size= -0.8786296. BAP1 shRNA#2 vs BAP1 shRNA#2 + FLAG-VHL: **p*＜0.05; [95% CI, 2.899 — 31.10]; effect size= 0.8671469. 3 μM Ctrl vs BAP1 shRNA#1: ***p*＜0.01; [95% CI, -27.70 — -6.299]; effect size= -0.8786648. Ctrl vs BAP1 shRNA#2: ***p*＜0.01; [95% CI, -30.03 — -8.632]; effect size= -0.8742615. BAP1 shRNA#2 vs BAP1 shRNA#2 + FLAG-VHL: ***p*＜0.01; [95% CI, 3.966 — 25.37]; effect size= 0.9030967. 10 μM Ctrl vs BAP1 shRNA#1: ***p*＜0.01; [95% CI, -27.42 — -4.578]; effect size= -0.8626475. Ctrl vs BAP1 shRNA#2: ***p*＜0.01; [95% CI, -31.42 — -8.578]; effect size= -0.8787362. BAP1 shRNA#2 vs BAP1 shRNA#2 + FLAG-VHL: **p*＜0.05; [95% CI, 3.244 — 26.09]; effect size= 0.8768742. Oxaliplatin: 5 μM Ctrl vs BAP1 shRNA#1: ***p*＜0.01; [95% CI, -38.63 — -10.71]; effect size= -0.9073264. Ctrl vs BAP1 shRNA#2: ***p*＜0.01; [95% CI, -39.63 — -11.71]; effect size= -0.925318. BAP1 shRNA#2 vs BAP1 shRNA#2 + FLAG-VHL: ***p*＜0.01; [95% CI, 9.375 — 37.29]; effect size= 0.9211017. 10 μM Ctrl vs BAP1 shRNA#1: ***p*＜0.01; [95% CI, -41.06 — -9.609]; effect size= -0.8931702. Ctrl vs BAP1 shRNA#2: ***p*＜0.01; [95% CI, -42.06 — -10.61]; effect size= -0.8999776. BAP1 shRNA#2 vs BAP1 shRNA#2 + FLAG-VHL: ***p*＜0.01; [95% CI, 6.609 — 38.06]; effect size= 0.892989. 20 μM Ctrl vs BAP1 shRNA#1: ***p*＜0.01; [95% CI, -39.34 — -9.992]; effect size= -0.8924869. Ctrl vs BAP1 shRNA#2: ***p*＜0.01; [95% CI, -41.01 — -11.66]; effect size= -0.9063468. BAP1 shRNA#2 vs BAP1 shRNA#2 + FLAG-VHL: ***p*＜0.01; [95% CI, 5.658 — 35.01]; effect size= 0.9002496. 40 μM Ctrl vs BAP1 shRNA#1: ***p*＜0.01; [95% CI, -37.25 — -10.08]; effect size= -0.9165566. Ctrl vs BAP1 shRNA#2: ***p*＜0.01; [95% CI, -37.92 — -10.75]; effect size= -0.8973333. BAP1 shRNA#2 vs BAP1 shRNA#2 + FLAG-VHL: ***p*＜0.01; [95% CI, 6.414 — 33.59]; effect size= 0.8862012. **(C)** PDAC patient-derived tumor xenografts (PDXs) were subcutaneously implanted into nude mice and xenograft were injected with lentivirus expressing indicated constructs when tumor volume reached 30 mm^3^. Mice were then treated with saline or gemcitabine (50 mg/kg three times a week), respectively (n=6 per group). Xenograft tumors were dissected, and tumor weights were measured. Results represent the mean ± s.d. from six mice. Statistical significance was determined by one-way ANOVA followed by Tukey's multiple comparisons test. Ctrl vs BAP1 shRNA#1: ****p*＜0.001; [95% CI, -1.433 — -0.7432]; effect size= -0.9364191. Ctrl vs Ctrl + Gemcitabine: ***p*＜0.01; [95% CI, 0.1182 — 0.8085]; effect size= 0.8067245. BAP1 shRNA#1 vs BAP1 shRNA#1 + FLAG-VHL: ****p*＜0.001; [95% CI, 0.5599 — 1.250]; effect size= 0.9685709. Ctrl + Gemcitabine vs BAP1 shRNA#1 + Gemcitabine: ****p*＜0.001; [95% CI, -1.520 — -0.8299]; effect size= -0.9502564. BAP1 shRNA#1+ Gemcitabine vs BAP1 shRNA#1 + FLAG-VHL + Gemcitabine: ****p*＜0.001; [95% CI, 0.7649 — 1.455]; effect size= 0.8629518. **(D)** BxPC3 cells stably expressing FLAG-BAP1 were infected with lentivirus expressing control or VHL shRNA, and western blot was performed with indicated antibodies. Data are representative of three independent experiments. **(E)** Cell proliferation assay was performed in BxPC3 cells. Results represent the mean ± s.d. of three independent experiments (biological replicates). Statistical significance was determined by one-way ANOVA followed by Tukey's multiple comparisons test. Day4 Vector vs FLAG-BAP1 WT: ****p*＜0.001; [95% CI, 21.04 — 42.96]; effect size= 0.9512726. FLAG-BAP1 WT vs FLAG-BAP1 WT + VHL shRNA: ***p*＜0.01; [95% CI, -28.80 — -6.871]; effect size= -0.9789511. Day5 Vector vs FLAG-BAP1 WT: ****p*＜0.001; [95% CI, 40.85 — 84.99]; effect size= 0.968524. FLAG-BAP1 WT vs FLAG-BAP1 WT + VHL shRNA: ***p*＜0.01; [95% CI, -62.49 — -18.35]; effect size= -0.3036433. Day6 Vector vs FLAG-BAP1 WT: ****p*＜0.001; [95% CI, 91.36 — 172.0]; effect size= 0.9797488. FLAG-BAP1 WT vs FLAG-BAP1 WT + VHL shRNA: ***p*＜0.01; [95% CI, -114.5 — -33.86]; effect size= -0.9306977.

**Supplementary Figure 7.**


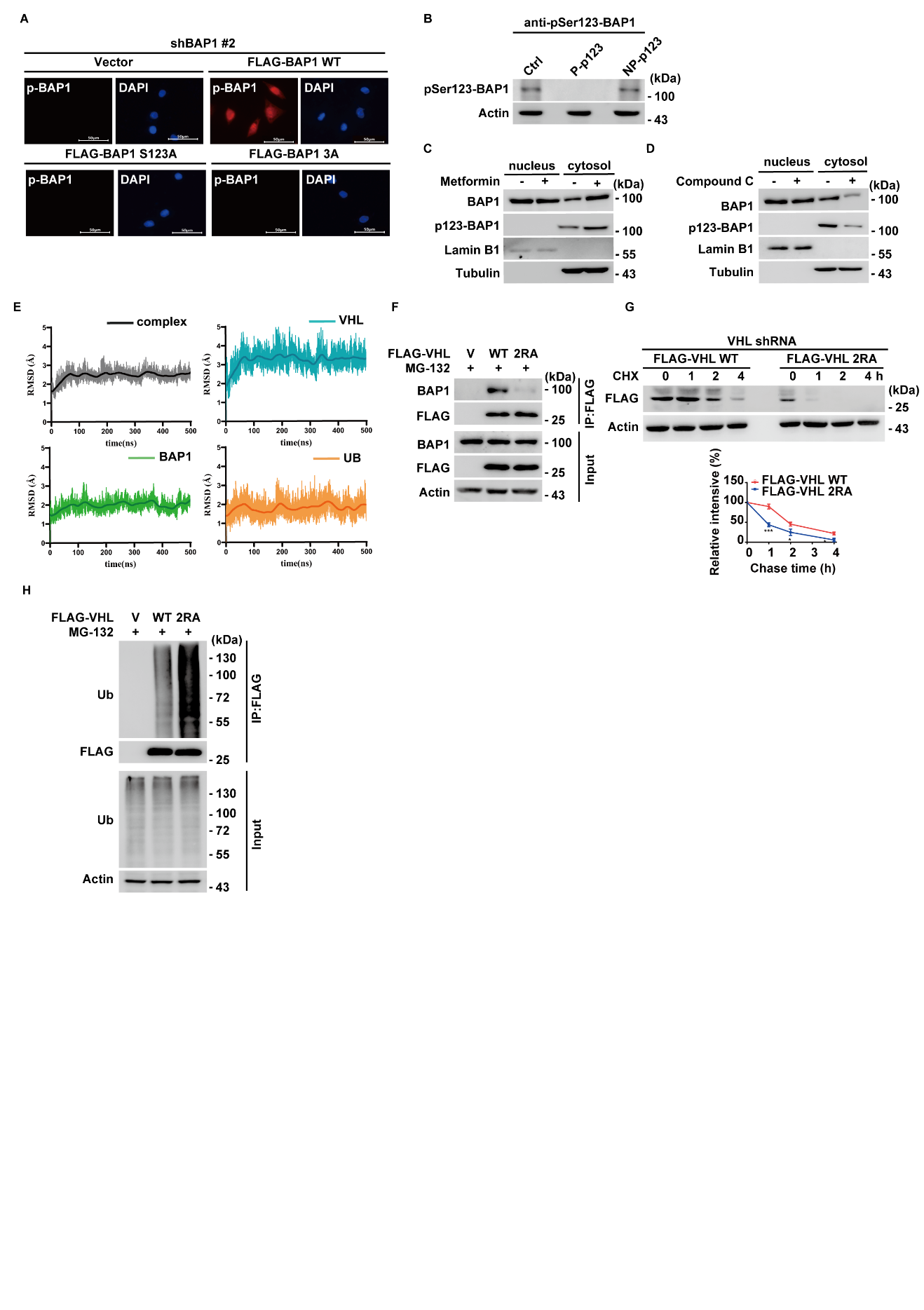


**Fig. S7. AMPKα-mediated phosphorylation of BAP1 regulates pVHL. (A)** Vector, FLAG-BAP1 WT or FLAG-BAP1 3A mutant were transfected in PANC-1 cells stably expressing BAP1 shRNA. Cells were detected by immunofluorescence staining. Scale bars, 50 μm. Data are representative of three independent experiments. **(B)** Validation of the phosphorylation specificity of the anti-pSer123-BAP1 antibody. The pSer123-BAP1 antibody (1 μg) was pre-incubated with 1 mL PBS containing BSA (Ctrl, 10μg), phosphorylated peptide (P-p123, 10 μg), or non-phosphorylated peptide (NP-p123, 10 μg) at 4°C for 2 h prior to immunoblotting of PANC-1 cell lysates. Data are representative of three independent experiments. **(C and D)** PANC-1 cells were treated with vehicle, 1 mM metformin for 24 hours (C), or 5 μM Compound C for 4 hours (D). Following treatment, nuclear and cytoplasmic fractions were isolated using a commercial subcellular fractionation kit, and western blotting was performed with the indicated antibodies. Data are representative of three independent experiments. **(E)** RMSD values for the 500 ns MD simulation. **(F)** HEK293T cells were transfected with vector, FLAG-VHL WT or the 2RA mutant (R82A/R161A) and treated with MG132 (10 μM) for 10 h before harvest. Cell lysates were subjected to immunoprecipitation with anti-FLAG antibody, followed by western blotting. Data are representative of three independent experiments. **(G)** PANC-1 cells stably expressing VHL shRNA were transfected with FLAG-VHL WT or the 2RA mutant. Cycloheximide pulse-chase assay was performed, and the relative FLAG-VHL level normalized to actin was quantified using image J. Results represent the mean ± s.d. of three independent experiments (biological replicates). 2RA, R82A/R161A. Statistical significance was determined by a two-tailed Student's t-test. 1h FLAG-VHL WT vs FLAG-VHL 2 RA: ****p*＜0.001; [95% CI, -58.56 — -33.28]; effect size= 0.9717586. 2h FLAG-VHL WT vs FLAG-VHL 2RA: **p*＜0.05; [95% CI, -35.67 — -5.233]; effect size= 0.836045. 4h FLAG-VHL WT vs FLAG-VHL 2RA: ***p*＜0.01（0.0072）; [95% CI, -25.38 — -7.408]; effect size= 0.9002673. **(H)** HEK293T cells were transfected with vector, FLAG-VHL WT or the 2RA mutant and treated with MG132 (10 μM) for 10 h before harvest. Cell lysates were subjected to immunoprecipitation with anti-FLAG antibody and pVHL ubiquitination was measured by western blotting with anti-ubiquitin antibody. Data are representative of three independent experiments.

**Supplementary Figure 8.**


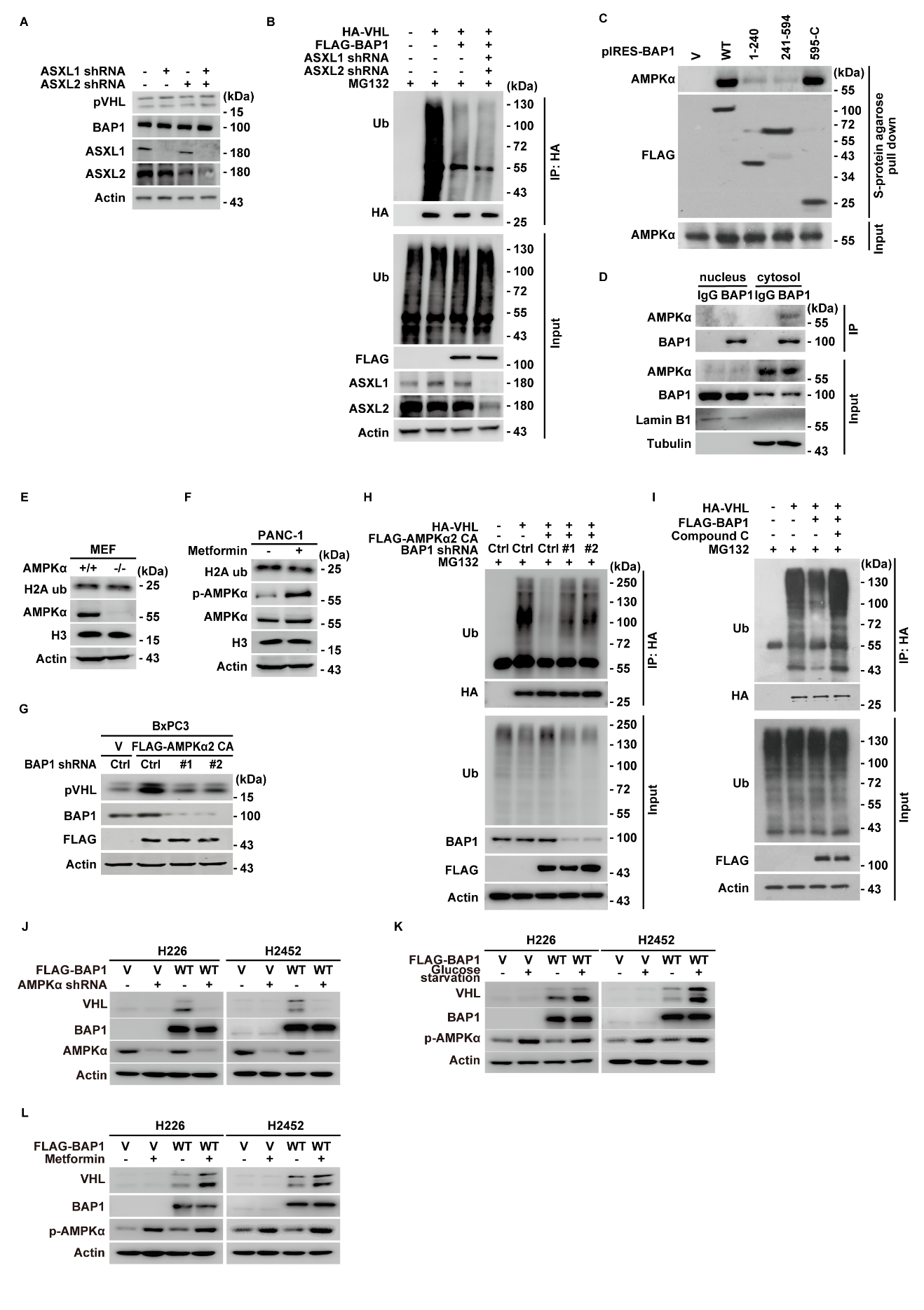
**Fig. S8. AMPKα phosphorylates BAP1 to promote pVHL deubiquitination and stabilization in the cytoplasm. (A)** PANC-1 cells were infected with lentivirus expressing control, ASXL1 shRNA, ASXL2 shRNA, or combined ASXL1/2 shRNAs, and western blotting was performed with the indicated antibodies. Data are representative of three independent experiments. **(B)** HEK293T cells stably expressing control or combined ASXL1/2 shRNAs were transfected as indicated and treated with MG-132 (10 μM) for 10 h before harvest. Cell lysates were subjected to immunoprecipitation with anti-HA magnetic beads and the pVHL ubiquitination was measured by western blotting with anti-ubiquitin antibody. Data are representative of three independent experiments. **(C)** pIRES vector, pIRES‐BAP1 WT, M1 (1-240 amino acids), M2 (241-594 amino acids) or M3 (595-C terminal) constructs were transfected in HEK293T cells. Cell lysates were subjected to S-protein agarose pulldown, and the interactions with AMPKα were detected by western blot. Data are representative of three independent experiments. **(D)** Subcellular fractionation and co-immunoprecipitation analysis of BAP1 in PANC-1 cells. Nuclear and cytoplasmic fractions were prepared using a commercial fractionation kit. Co-immunoprecipitation was performed from each fraction using either control IgG or anti-BAP1 antibody, followed by immunoblotting with the indicated antibodies. Data are representative of three independent experiments. **(E)** Cell lysates from AMPKα1/2 wild type (AMPKα^+/+^) or AMPKα1/2 double knockout (AMPKα^-/-^) mouse embryonic fibroblast cells (MEFs) were subjected to immunoblotting with indicated antibodies. Data are representative of three independent experiments. **(F)** PANC-1 cells were treated with vehicle or metformin (1mM) for 24 h. Western blotting was performed with the indicated antibodies. Data are representative of three independent experiments. **(G)** BxPC3 cells stably expressing FLAG-AMPKα2 CA were infected with lentivirus expressing control or BAP1 shRNAs, and western blot was performed with indicated antibodies. Data are representative of three independent experiments. **(H)** HEK293T cells stably expressing control or BAP1 shRNAs were transfected with indicated plasmids and treated with MG132 (10 μM) for 10 h before harvest. Cell lysates were subjected to immunoprecipitation with anti-HA magnetic beads and the ubiquitination of pVHL was measured by western blot with anti-ubiquitin antibody. Data are representative of three independent experiments. **(I)** Cells stably expressing vector or FLAG-BAP1 were transfected with indicated plasmids and treated with vehicle or Compound C (5 μM) for 4 h. Cell lysates were subjected to immunoprecipitation with anti-HA magnetic beads and the ubiquitination of pVHL was measured by western blot with anti-ubiquitin antibody. Data are representative of three independent experiments. **(J)** H226 and H2452 cells stably expressing control or AMPKα shRNA were transfected with vector or FLAG-BAP1, and western blot was performed with indicated antibodies. Data are representative of three independent experiments. **(K)** H226 and H2452 cells stably expressing vector or FLAG-BAP1 were cultured with glucose starvation medium (1 mM glucose) or normal DMEM for 12 h, and western blot was performed with indicated antibodies. Data are representative of three independent experiments. **(L)** H226 and H2452 cells stably expressing vector or FLAG-BAP1 were treated with vehicle or metformin (1 mM) for 24 h. Western blot was performed with indicated antibodies. Data are representative of three independent experiments.

**Supplementary Figure 9.**


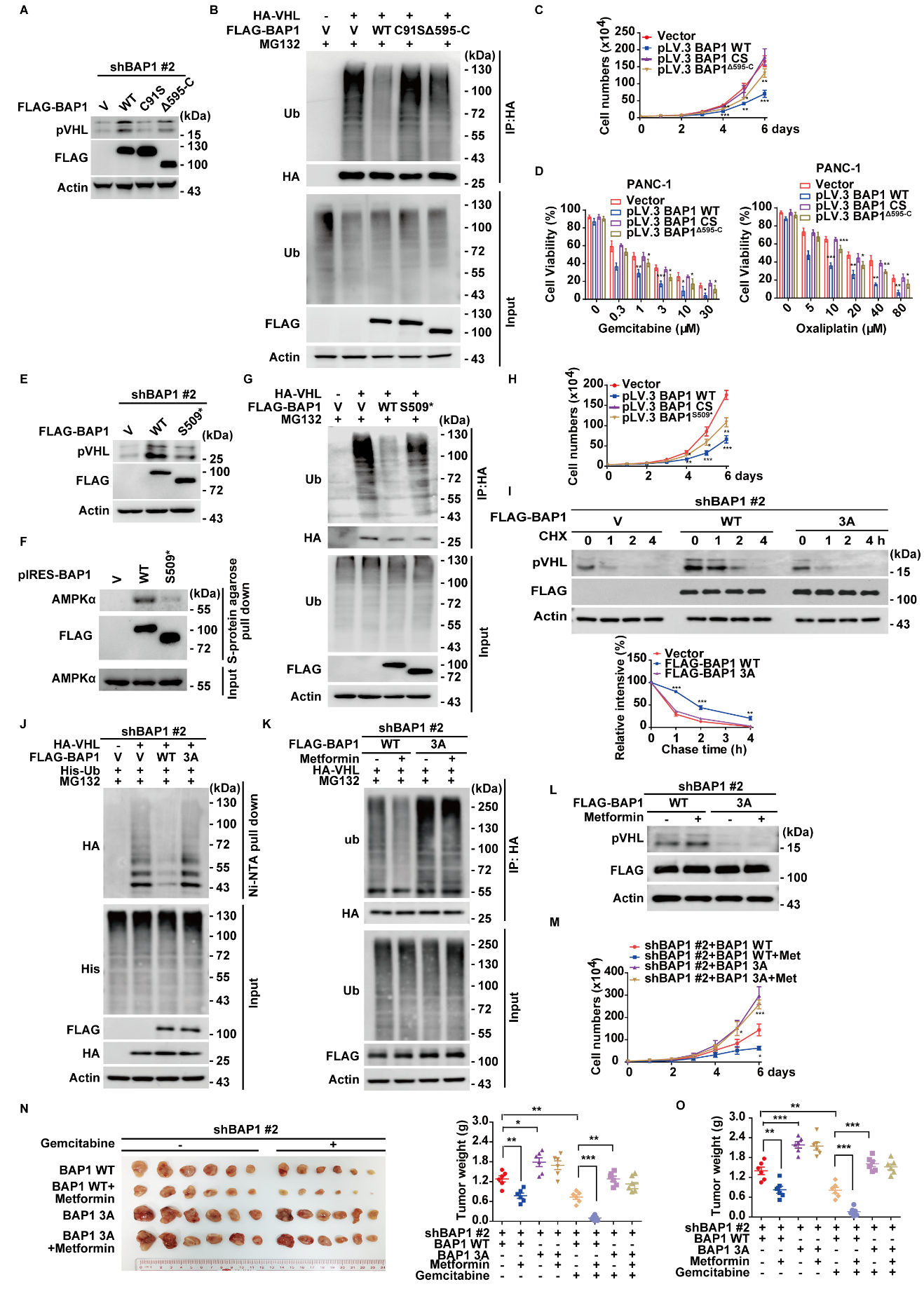


**Fig. S9. AMPKα-mediated phosphorylation of BAP1 regulates the stability and tumor-suppressive function of pVHL. (A)** PANC-1 cells stably expressing BAP1 shRNA were transfected with vector, BAP1 WT, C91S or the AMPK-binding region deletion mutant (Δ595-C). Western blotting was performed with the indicated antibodies. Data are representative of three independent experiments. **(B)** Cells stably expressing BAP1 shRNA were transfected with indicated plasmids. Cell lysates were subjected to immunoprecipitation with anti-HA magnetic beads, and pVHL ubiquitination was measured by western blotting with anti-ubiquitin antibody. Data are representative of three independent experiments. **(C)** Cell proliferation assay was performed in PANC-1 cells as in (A). Results represent the mean ± s.d. of three independent experiments (biological replicates). Statistical significance was determined by one-way ANOVA followed by Tukey's multiple comparisons test. Day4 Vector vs FLAG-BAP1 WT: ****p*＜0.001; [95% CI, 13.55 — 22.45]; effect size= 0.9901176. Vector vs FLAG-BAP1 CS: *P*＞0.05; [95% CI, -0.7828 — 8.116]; effect size= 0.7713796. FLAG-BAP1 WT vs FLAG-BAP1^Δ595-C^: ***p*＜0.01; [95% CI, -10.95 — -2.051; effect size= -0.8666969. Day5 Vector vs FLAG-BAP1 WT: ***p*＜0.01; [95% CI, 16.50 — 76.00]; effect size= 0.9243253. Vector vs FLAG-BAP1 CS: *P*＞0.05; [95% CI, -18.91 — 40.58]; effect size= 0.3257411. FLAG-BAP1 WT vs FLAG-BAP1^Δ595-C^: **p*＜0.05; [95% CI, -43.91 — 15.58]; effect size= 0.9849083. Day6 Vector vs FLAG-BAP1 WT: ****p*＜0.001; [95% CI, 52.76 — 139.7]; effect size= 0.9767248. Vector vs FLAG-BAP1 CS: *P*＞0.05; [95% CI, -55.99 — 30.99]; effect size= -0.6978777. FLAG-BAP1 WT vs FLAG-BAP1^Δ595-C^: ***p*＜0.01; [95% CI, -104.7 — -17.76]; effect size= -0.9591365. (**D)** PANC-1 cells as in (A) were treated with the indicated concentrations of gemcitabine or oxaliplatin, and cell viability was determined. Results represent the mean ± s.d. of three independent experiments (biological replicates). Statistical significance was determined by one-way ANOVA followed by Tukey's multiple comparisons test. **Gemcitabine:** 1 μM Vector vs FLAG-BAP1 WT: ***p*＜0.01; [95% CI, 7.907 — 30.09]; effect size= 0.918056. Vector vs FLAG-BAP1 CS: *P*＞0.05; [95% CI, -10.76 — 11.43]; effect size= 0.0381621. FLAG-BAP1 WT vs FLAG-BAP1^Δ595-C^: **p*＜0.05; [95% CI, -22.43 — -0.2401]; effect size= -0.8060659. 3 μM Vector vs FLAG-BAP1 WT: ****p*＜0.001; [95% CI, 9.906 — 26.09]; effect size= 0.9443148. Vector vs FLAG-BAP1 CS: *P*＞0.05; [95% CI, -6.094 — 10.09]; effect size= 0.3364476. FLAG-BAP1 WT vs FLAG-BAP1^Δ595-C^: **p*＜0.05; [95% CI, -15.09 — 1.094]; effect size= -0.7206898. 10 μM Vector vs FLAG-BAP1 WT: **p*＜0.05; [95% CI, 4.210 — 27.79]; effect size= 0.8560043. Vector vs FLAG-BAP1 CS: *P*＞0.05; [95% CI, -12.12 — 11.46]; effect size= -0.0504342. FLAG-BAP1 WT vs FLAG-BAP1^Δ595-C^: **p*＜0.05; [95% CI, -19.79 — 3.790]; effect size= -0.7939399. 30 μM Vector vs FLAG-BAP1 WT: **p*＜0.05; [95% CI, 2.836 — 19.16]; effect size= 0.9092618. Vector vs FLAG-BAP1 CS: *P*＞0.05; [95% CI, -10.83 — 5.498]; effect size= -0.4342785. FLAG-BAP1 WT vs FLAG-BAP1^Δ595-C^: **p*＜0.05; [95% CI, -15.16 — 1.164]; effect size= -0.7130531. **Oxaliplatin:** 10 μM Vector vs FLAG-BAP1 WT: ****p*＜0.001; [95% CI, 21.17 — 37.50]; effect size= 0.9793466. Vector vs FLAG-BAP1 CS: *P*＞0.05; [95% CI, -8.164 — 8.164]; effect size= 0. FLAG-BAP1 WT vs FLAG-BAP1^Δ595-C^: ****p*＜0.001; [95% CI, -26.50 — -10.17]; effect size= -0.93219. 20 μM Vector vs FLAG-BAP1 WT: ***p*＜0.01; [95% CI, 10.45 — 32.22]; effect size= 0.9351683. Vector vs FLAG-BAP1 CS: *P*＞0.05; [95% CI, -7.886 — 13.89]; effect size= 0.3389486. FLAG-BAP1 WT vs FLAG-BAP1^Δ595-C^: **p*＜0.05; [95% CI, -21.22 — 0.5526]; effect size= -0.7788641. 40 μM Vector vs FLAG-BAP1 WT: ****p*＜0.001; [95% CI, 17.43 — 35.23]; effect size= 0.9559924. Vector vs FLAG-BAP1 CS: *P*＞0.05; [95% CI, -5.899 — 11.90]; effect size= 0.3191811. FLAG-BAP1 WT vs FLAG-BAP1^Δ595-C^: ***p*＜0.01; [95% CI, -22.90 — -5.101]; effect size= -0.9676158. 80 μM Vector vs FLAG-BAP1 WT: ***p*＜0.01; [95% CI, 6.159 — 25.84]; effect size= 0.9299668. Vector vs FLAG-BAP1 CS: *P*＞0.05; [95% CI, -10.51 — 9.175]; effect size= -0.0871419. FLAG-BAP1 WT vs FLAG-BAP1^Δ595-C^: **p*＜0.05; [95% CI, -19.51 — 0.1747]; effect size= -0.7944031. **(E)** PANC-1 cells stably expressing BAP1 shRNA were transfected with vector, BAP1 WT or S509* mutation, and western blotting was performed with the indicated antibodies. Data are representative of three independent experiments. **(F)** Cells stably expressing BAP1 shRNA were transfected with the indicated plasmids. Cell lysates were pull-downed by S-protein agaroses, and western blotting was performed. Data are representative of three independent experiments. **(G)** Cells stably expressing BAP1 shRNA were transfected as indicated. Cell lysates were subjected to immunoprecipitation with anti-HA magnetic beads and pVHL ubiquitination was measured by western blotting with anti-ubiquitin antibody. Data are representative of three independent experiments. **(H)** Cell proliferation assay was performed in PANC-1 cells as in (E). Results represent the mean ± s.d. of three independent experiments (biological replicates). Statistical significance was determined by one-way ANOVA followed by Tukey's multiple comparisons test. Day4 Vector vs FLAG-BAP1 WT: ***p*＜0.01; [95% CI, 9.165 — 24.50]; effect size= 0.9351419. FLAG-BAP1 WT vs FLAG-BAP1^S509*^: **p*＜0.05; [95% CI, -16.83 — -1.499]; effect size= -0.898621. Day5 Vector vs FLAG-BAP1 WT: ****p*＜0.001; [95% CI, 31.91 — 74.42]; effect size= 0.9485037. FLAG-BAP1 WT vs FLAG-BAP1^S509*^: **p*＜0.05; [95% CI, -47.76 — -5.242]; effect size= -0.8932692. Day6 Vector vs FLAG-BAP1 WT: ****p*＜0.001; [95% CI, 83.09 — 136.1]; effect size= 0.9830854. FLAG-BAP1 WT vs FLAG-BAP1^S509*^: ***p*＜0.01; [95% CI, -68.57 — -15.59]; effect size= -0.899636. **(I)** BxPC3 cells stably expressing BAP1 shRNA were transfected with vector, BAP1 WT or its phosphorylation mutant 3A, and cycloheximide pulse-chase assay was performed. Results are quantified. Results represent the mean ± s.d. of three independent experiments (biological replicates). Statistical significance was determined by one-way ANOVA followed by Tukey's multiple comparisons test. 1h Vector vs FLAG-BAP1 WT: ****p*＜0.001; [95% CI, -63.91 — -36.80]; effect size= -0.9763984. Vector vs FLAG-BAP1 3A: *P*＞0.05; [95% CI, -20.55 — 6.552]; effect size= -0.4859466. 2h Vector vs FLAG-BAP1 WT: ****p*＜0.001; [95% CI, -43.78 — -18.09]; effect size= -0.9365628. Vector vs FLAG-BAP1 3A: *P*＞0.05; [95% CI, -19.49 — 6.207]; effect size= -0.6688828. 4h Vector vs FLAG-BAP1 WT: ***p*＜0.01; [95% CI, -30.11 — -7.367]; effect size= -0.8767316. Vector vs FLAG-BAP1 3A: *P*＞0.05; [95% CI, -12.96 — 9.777]; effect size= -0.3252721. **(J)** Cells were cotransfected with indicated plasmids and Ni-NTA bead was used to pull down His-tagged ubiquitin, and the ubiquitination of pVHL was measured by western blot. Data are representative of three independent experiments. **(K)** Cells were transfected with indicated plasmids and treated with vehicle or metformin (1 mM) for 24 h. Cell lysates were subjected to immunoprecipitation with anti-HA magnetic beads and the ubiquitination of pVHL was measured by western blot with anti-ubiquitin antibody. Data are representative of three independent experiments. **(L)** BxPC3 cells stably expressing BAP1 shRNA were transfected with indicated plasmids and treated with vehicle or metformin (2 mM) for 24 h. Western blot was performed with indicated antibodies. Data are representative of three independent experiments. **(M)** Cell proliferation assay was performed in BxPC3 cells as in (L). Results represent the mean ± s.d. of three independent experiments (biological replicates). Statistical significance was determined by one-way ANOVA followed by Tukey's multiple comparisons test. Day5 FLAG-BAP1 WT vs FLAG-BAP1 WT + Metformin: *P*＞0.05; [95% CI, -36.47 — 100.1]; effect size= 0.8492592. FLAG-BAP1 WT vs FLAG-BAP1 3A: **p*＜0.05; [95% CI, -137.5 — -0.8678]; effect size= 0.3789639. Day6 FLAG-BAP1 WT vs FLAG-BAP1 WT + Metformin: **p*＜0.05; [95% CI, 12.35 — 150.3]; effect size= 0.9014213. Day6 FLAG-BAP1 WT vs FLAG-BAP1 3A: ****p*＜0.001; [95% CI, -222.3 — -84.35]; effect size= -0.9142351. **(N)** PANC-1 cells (1×10^6^) were subcutaneously implanted into nude mice. When tumor reached 100 mm^3^, mice were treated with saline, gemcitabine (50 mg/kg three times a week) or metformin (100 mg/kg every two days) (n=6 per group). Xenograft tumors were dissected, and tumor weights were measured in right panel. Results represent the mean ± s.d. from six mice. Statistical significance was determined by one-way ANOVA followed by Tukey's multiple comparisons test. FLAG-BAP1 WT vs FLAG-BAP1 WT + Metformin: ***p*＜0.01; [95% CI, 0.08277 — 0.9372]; effect size= 0.7741499. FLAG-BAP1 WT vs FLAG-BAP1 3A: **p*＜0.05; [95% CI, -0.9322 — -0.07777]; effect size= -0.6667851. FLAG-BAP1 WT vs FLAG-BAP1 WT + Gemcitabine: ***p*＜0.01; [95% CI, 0.1228 — 0.9772]; effect size= 0.8115961. FLAG-BAP1 WT + Gemcitabine vs FLAG-BAP1 WT + Metformin + Gemcitabine: ****p*＜0.001; [95% CI, 0.2078 — 1.062]; effect size=0.9276355. FLAG-BAP1 WT + Gemcitabine vs FLAG-BAP1 3A + Gemcitabine: ***p*＜0.01; [95% CI, -0.9772 — -0.1228]; effect size= -0.8304126. **(O)** PDAC PDXs were subcutaneously implanted into nude mice and xenograft were injected with lentivirus expressing indicated constructs when tumor volume reached 30 mm^3^. Mice were then treated with saline, gemcitabine (50 mg/kg three times a week) or metformin (100 mg/kg every two days) (n=6 per group). Xenograft tumors were dissected, and tumor weights were measured. Results represent the mean ± s.d. from six mice. Statistical significance was determined by one-way ANOVA followed by Tukey's multiple comparisons test. FLAG-BAP1 WT vs FLAG-BAP1 WT + Metformin: ***p*＜0.01; [95% CI, 0.1440 — 1.006]; effect size= 0.7335313. FLAG-BAP1 WT vs FLAG-BAP1 3A: ****p*＜0.001; [95% CI, -1.214 — -0.3523]; effect size= 0.7926746. FLAG-BAP1 WT vs FLAG-BAP1 WT + Gemcitabine: ***p*＜0.01; [95% CI, 0.1623 — 1.024]; effect size= 0.762169. FLAG-BAP1 WT + Gemcitabine vs FLAG-BAP1 WT + Met + Gemcitabine: ****p*＜0.001; [95% CI, 0.2140 — 1.076]; effect size= 0.870867. FLAG-BAP1 WT + Gemcitabine vs FLAG-BAP1 3A + Gemcitabine: ****p*＜0.001; [95% CI, -1.236 — -0.3740]; effect size= -0.8801274.

**Supplementary Figure 10.**


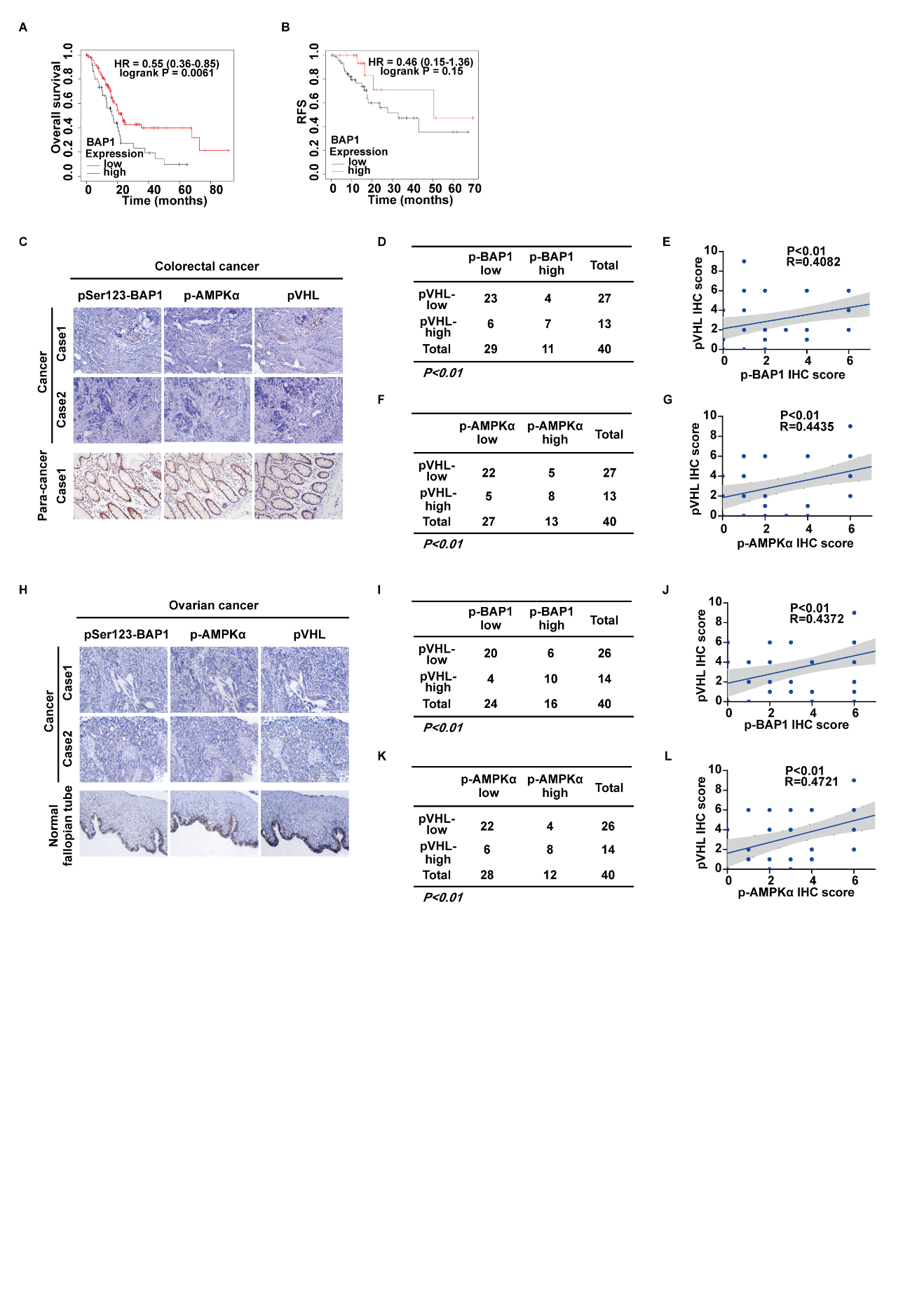


**Fig. S10. Expressions of p-AMPKα and pSer123-BAP1 positively correlates with pVHL levels in colorectal cancer and ovarian cancer. (A and B)** Analysis of the correlation between *BAP1* and the prognosis of clinical PDAC patient survival. Kaplan-Meier survival curves for overall survival (A) and RFS (B) was conducted using the KM Plotter database (http://kmplot.com). *p*-value is shown in the graph. **(C)** Representative immunohistochemical staining of pVHL, pSer123-BAP1 and p-AMPKα in colorectal cancer and adjacent normal tissues. Scale bar, 100 μm. (**D and E)** Positive correlation of pVHL expression and pSer123-BAP1 expression in colorectal cancer tissues. (**F and G**) Positive correlation of pVHL expression with p-AMPKα expression in colorectal cancer tissues. **(H)** Representative immunohistochemical staining of pVHL, pSer123-BAP1 and p-AMPKα in ovarian cancer and normal fallopian tube tissues. Scale bar, 100 μm. (**I and J)** Positive correlation of pVHL expression with pSer123-BAP1 expression in ovarian cancer tissues. (**K and L**) Positive correlation of pVHL expression with p-AMPKα expression in ovarian cancer tissues.

**References**

1. Huang W, Zhong Z, Luo C, Xiao Y, Li L, Zhang X*, et al.* The miR-26a/AP-2alpha/Nanog signaling axis mediates stem cell self-renewal and temozolomide resistance in glioma. *Theranostics* 2019, **9**(19)**:** 5497-5516.

2. Ma N, Hu J, Zhang ZM, Liu W, Huang M, Fan Y*, et al.* 2H-Azirine-Based Reagents for Chemoselective Bioconjugation at Carboxyl Residues Inside Live Cells. *J Am Chem Soc* 2020, **142**(13)**:** 6051-6059.

3. Ma J, Chen T, Wu S, Yang C, Bai M, Shu K*, et al.* iProX: an integrated proteome resource. *Nucleic Acids Res* 2019, **47**(D1)**:** D1211-D1217.

4. Chen T, Ma J, Liu Y, Chen Z, Xiao N, Lu Y*, et al.* iProX in 2021: connecting proteomics data sharing with big data. *Nucleic Acids Res* 2022, **50**(D1)**:** D1522-D1527.

5. Gyorffy B. Integrated analysis of public datasets for the discovery and validation of survival-associated genes in solid tumors. *Innovation (Camb)* 2024, **5**(3)**:** 100625.
