# Supplementary material for: AMPK-activated BAP1 regulates pVHL stability and tumor-suppressive functions
Source: Cell Death Differ. 2025 Sep 27;33(3):447–64. doi: 10.1038/s41418-025-01590-9 (PMC13035857; doi:10.1038/s41418-025-01590-9)
Supplement: Supplementary file 2 — Original western blots [file 41418_2025_1590_MOESM2_ESM.docx]

**AMPK-activated BAP1 Regulates pVHL Stability and Tumor-suppressive Functions**

**Supplementary Text2**

**Supplementary Figure 11**


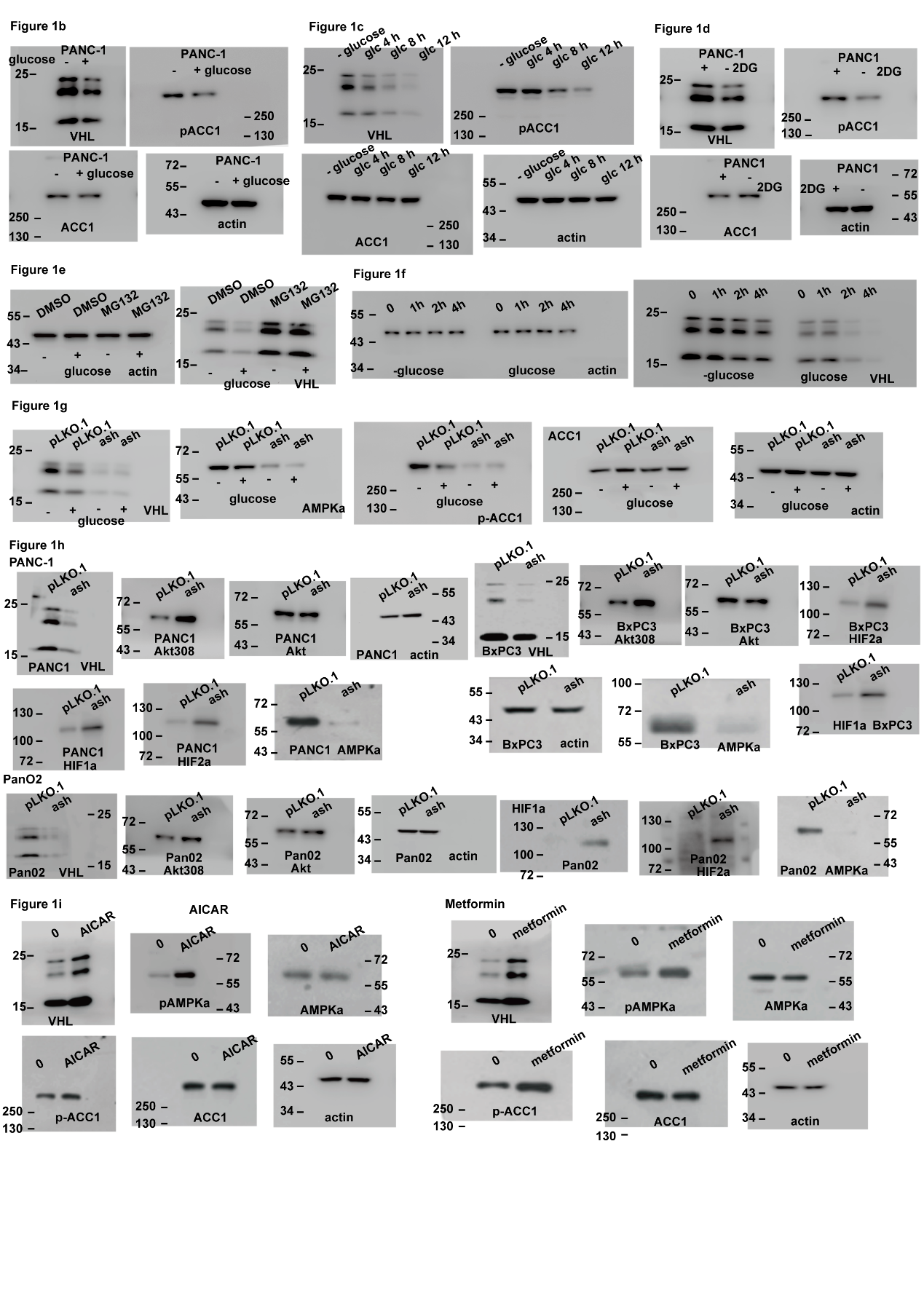


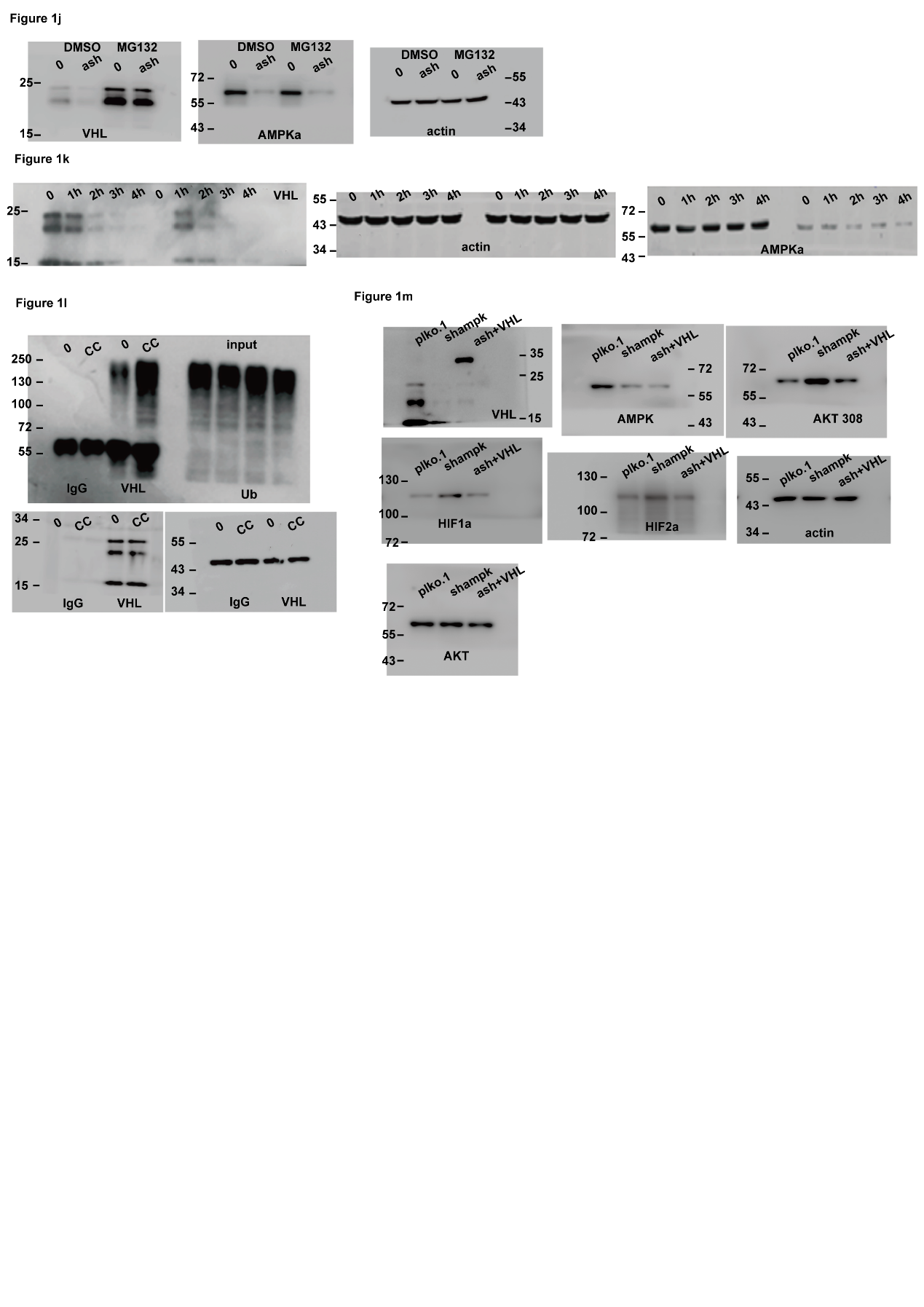


Supplementary Figure 11: Original scan of the blots presented in the main text. Related to Fig. 1.

**Supplementary Figure 12**


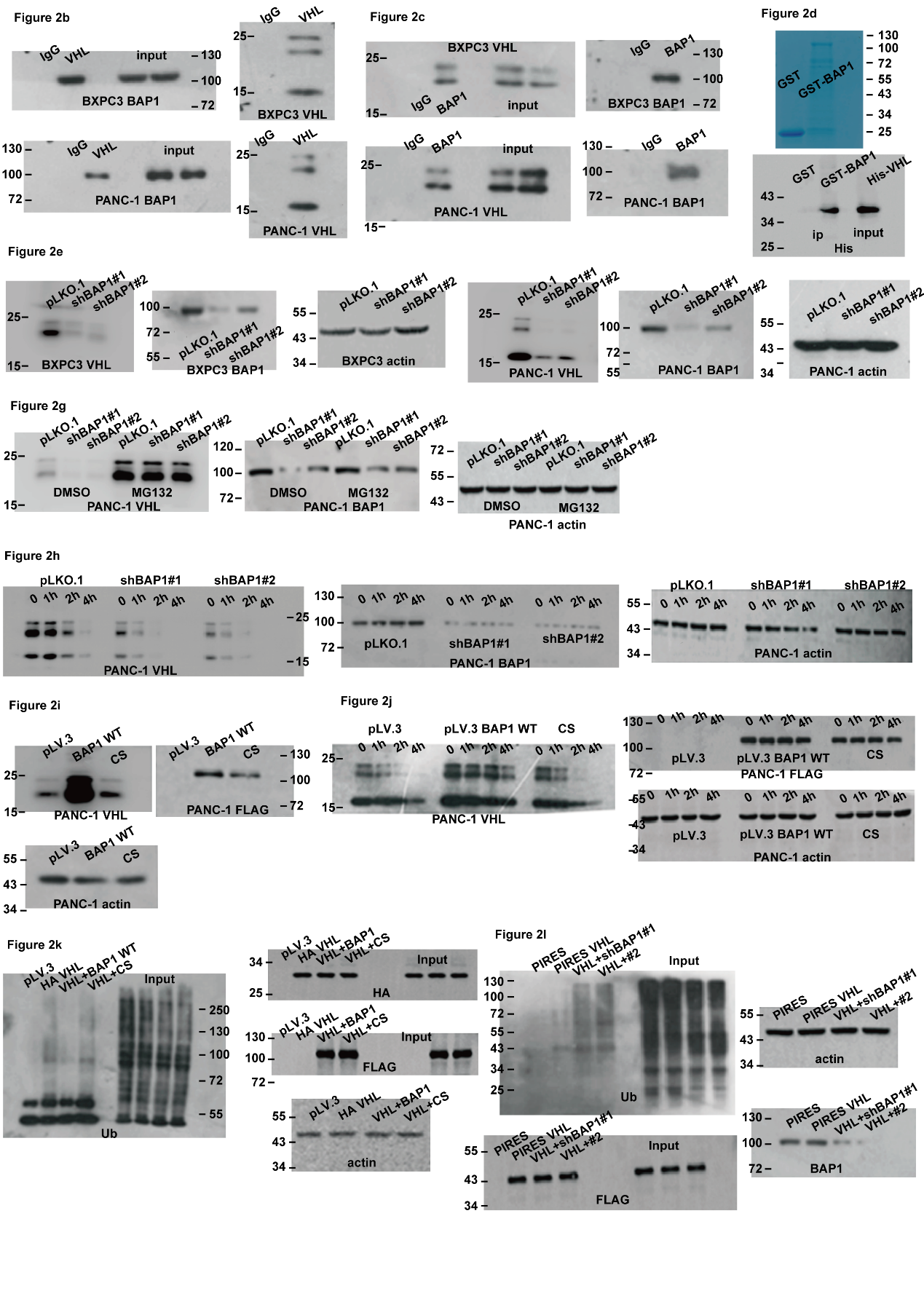


Supplementary Figure 12: Original scan of the blots presented in the main text. Related to Fig. 2.

**Supplementary Figure 13**


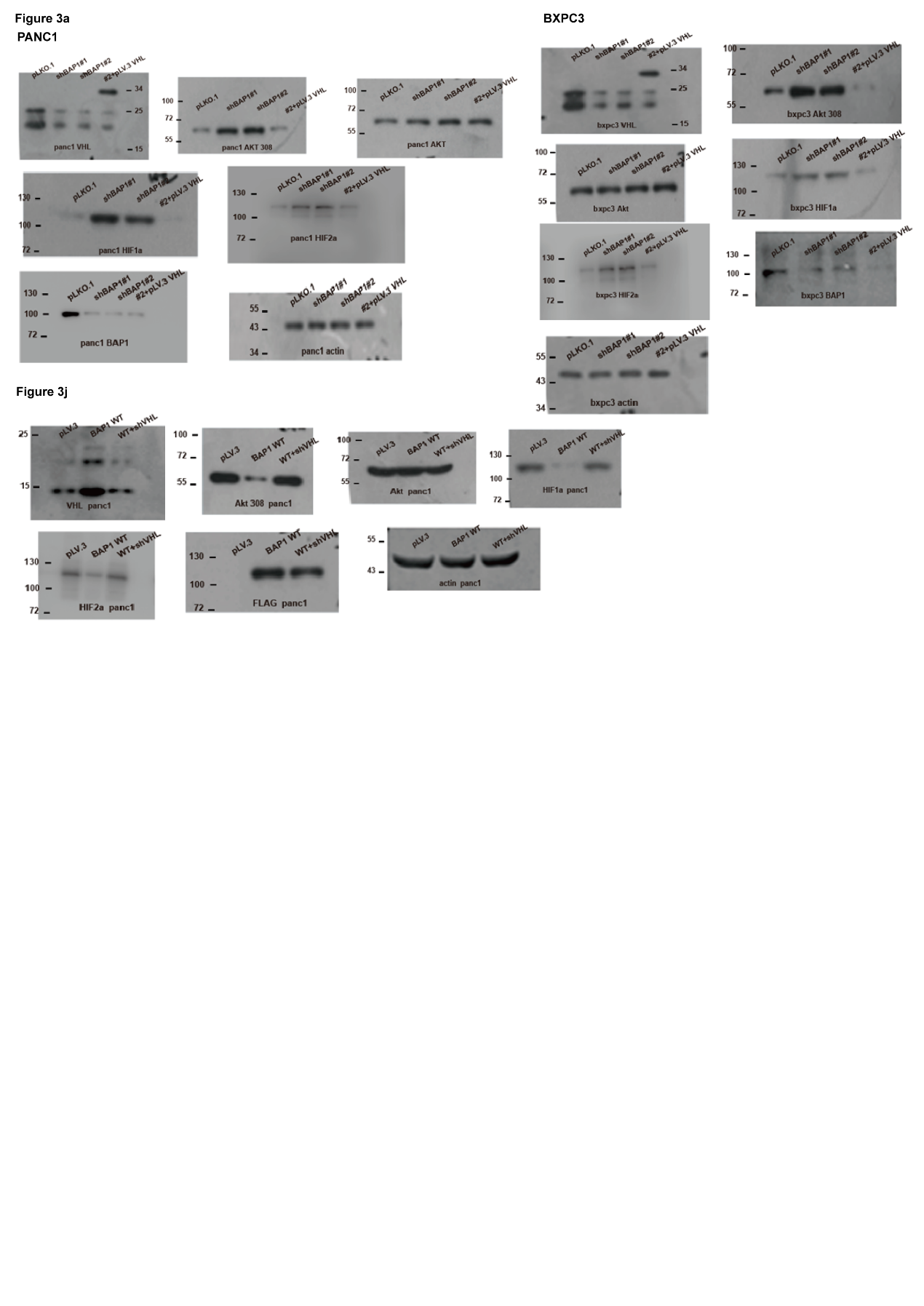


Supplementary Figure 13: Original scan of the blots presented in the main text. Related to Fig. 3.

**Supplementary Figure 14**


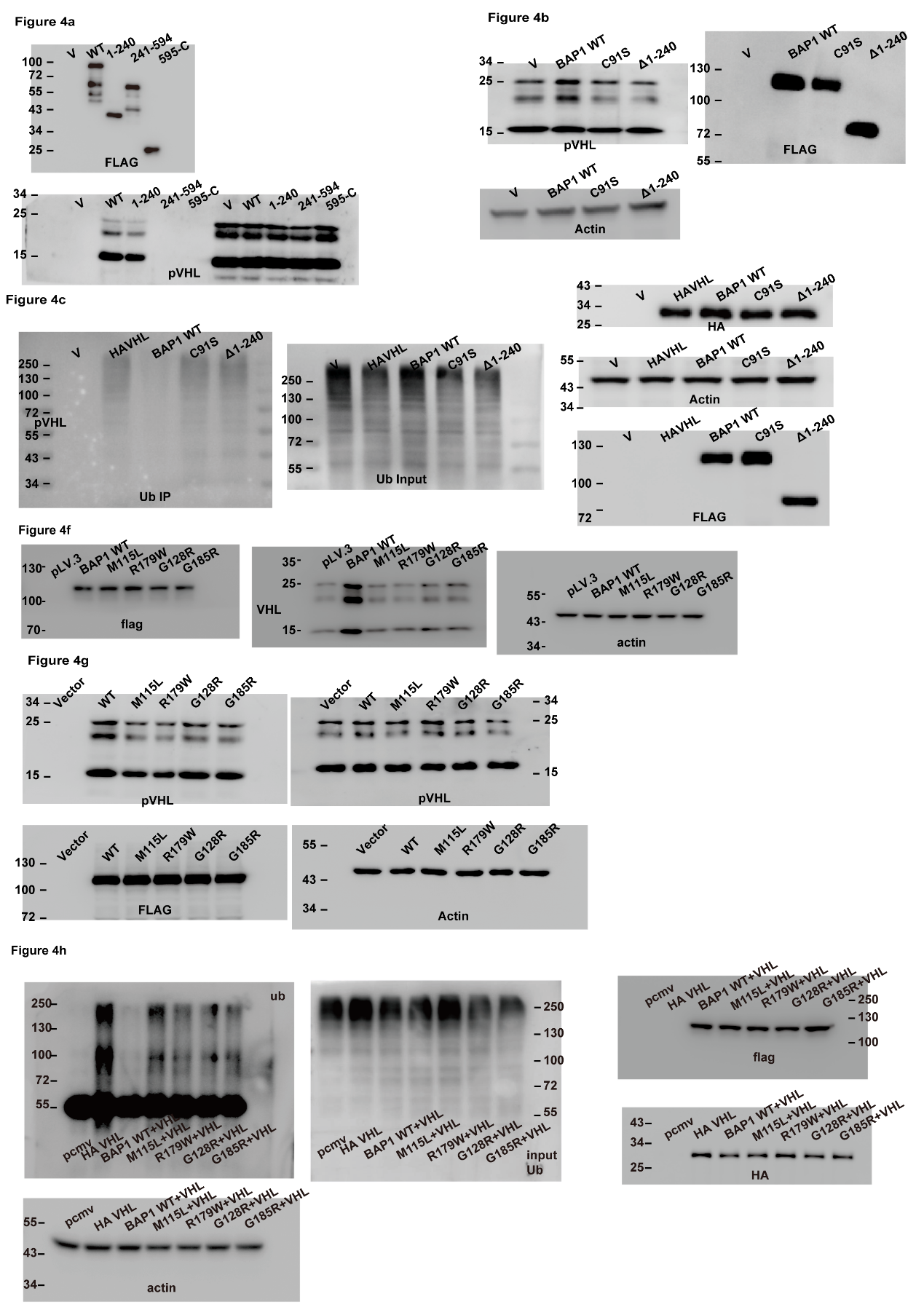


Supplementary Figure 14: Original scan of the blots presented in the main text. Related to Fig. 4.

**Supplementary Figure 15**


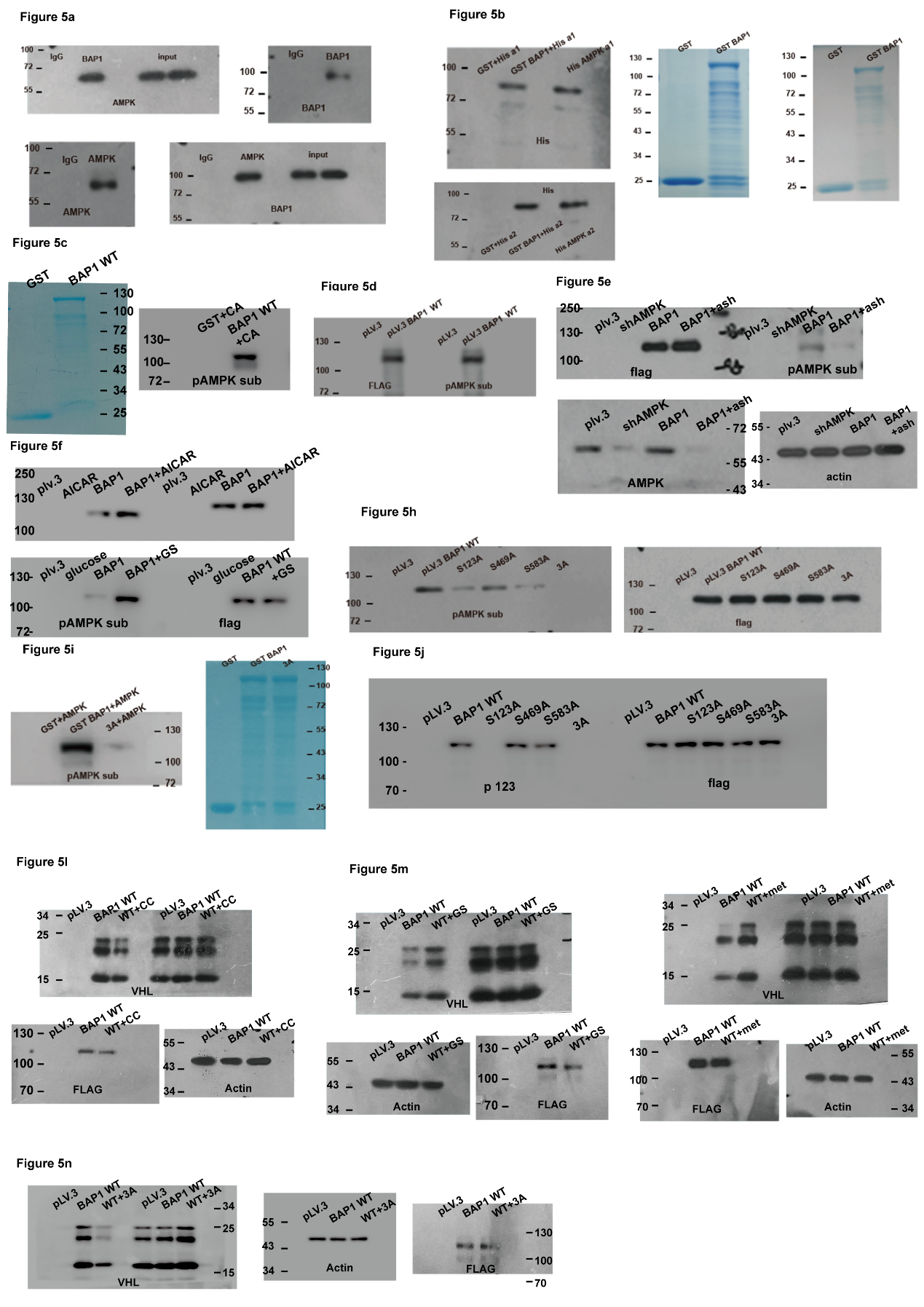


Supplementary Figure 15: Original scan of the blots presented in the main text. Related to Fig. 5.

**Supplementary Figure 16**


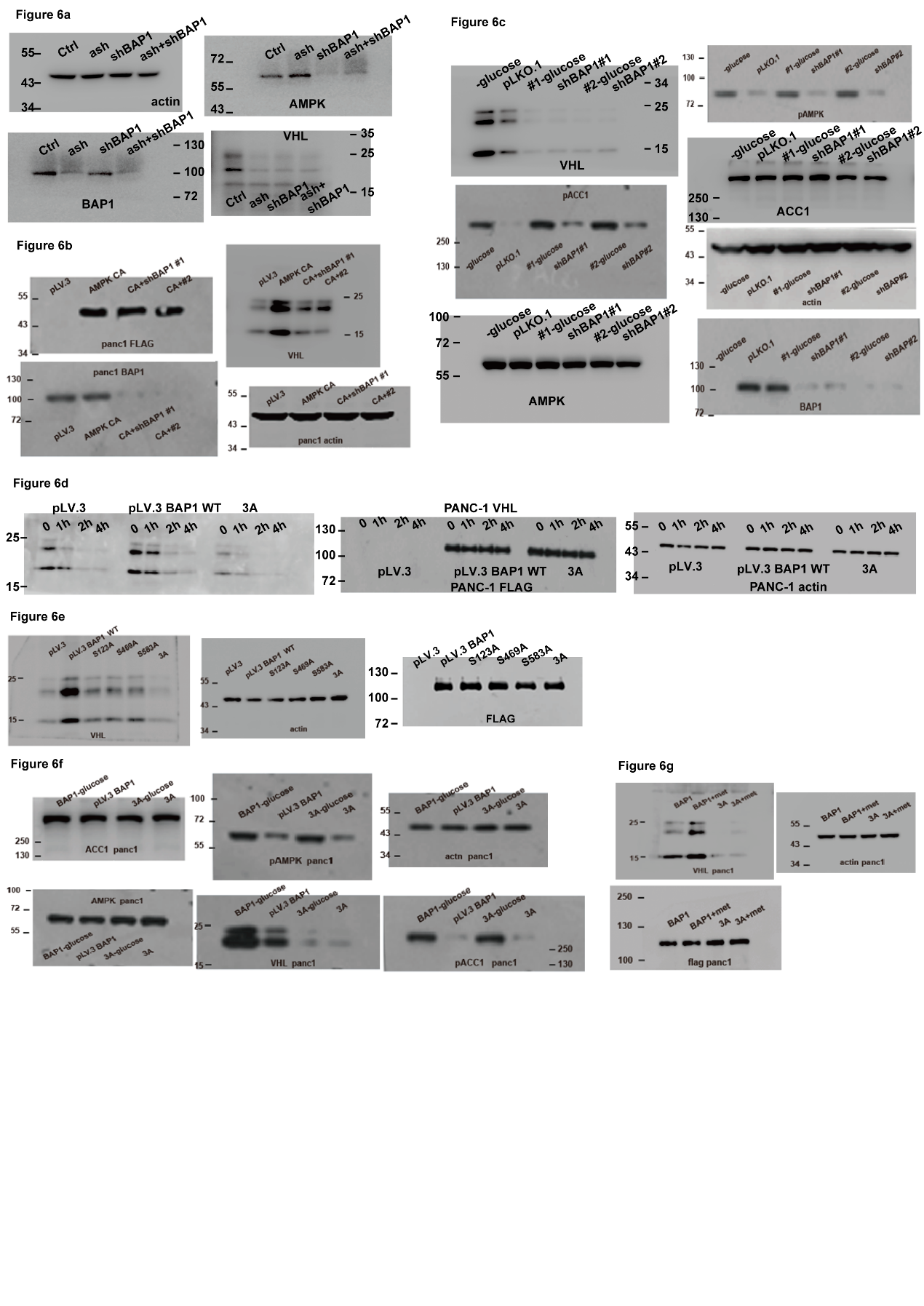


Supplementary Figure 16: Original scan of the blots presented in the main text. Related to Fig. 6.

**Supplementary Figure 17**


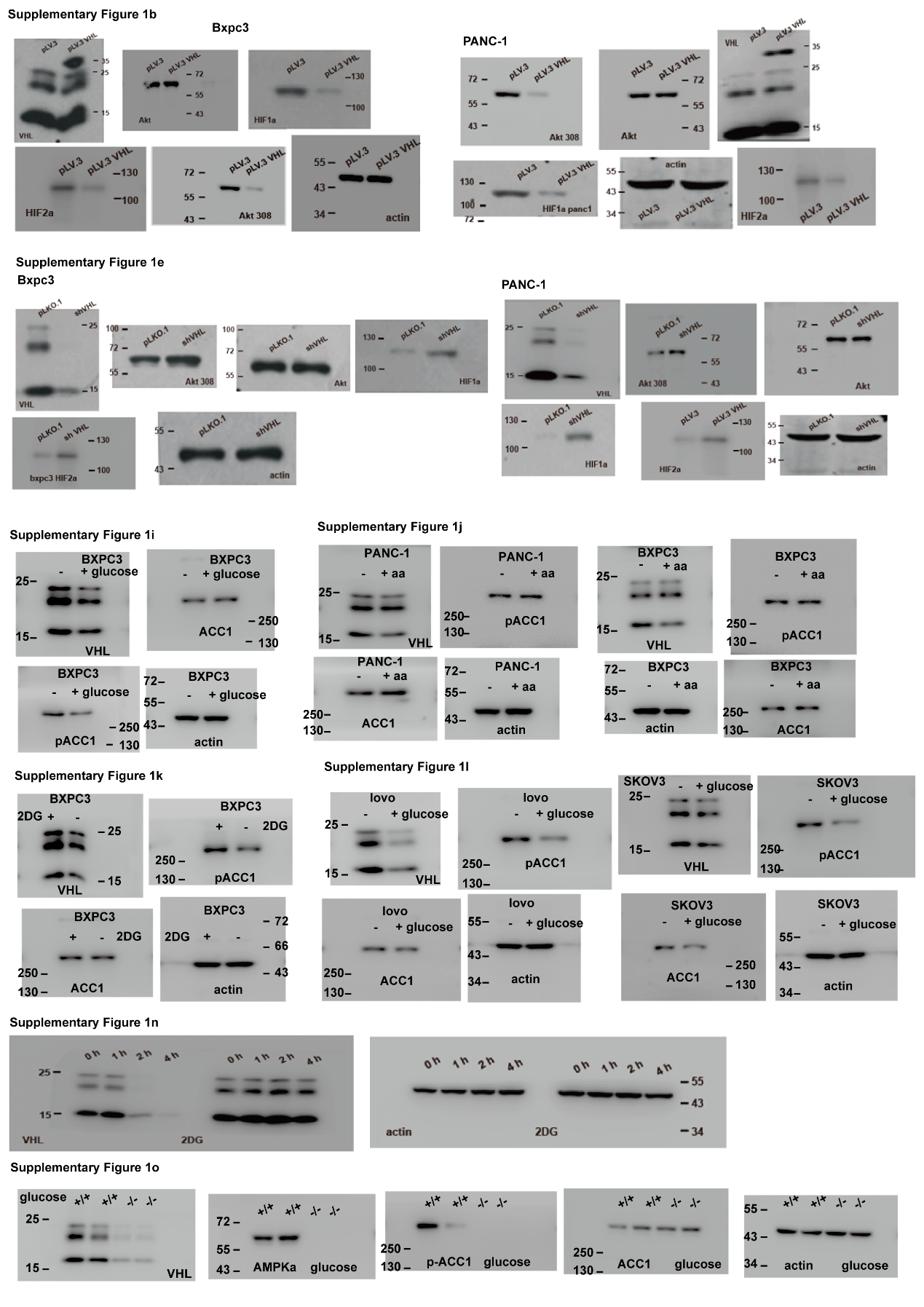


Supplementary Fig. 17: Original scan of the blots presented in the Supplementary Text 1. Related to Supplementary Fig. 1.

**Supplementary Figure 18**


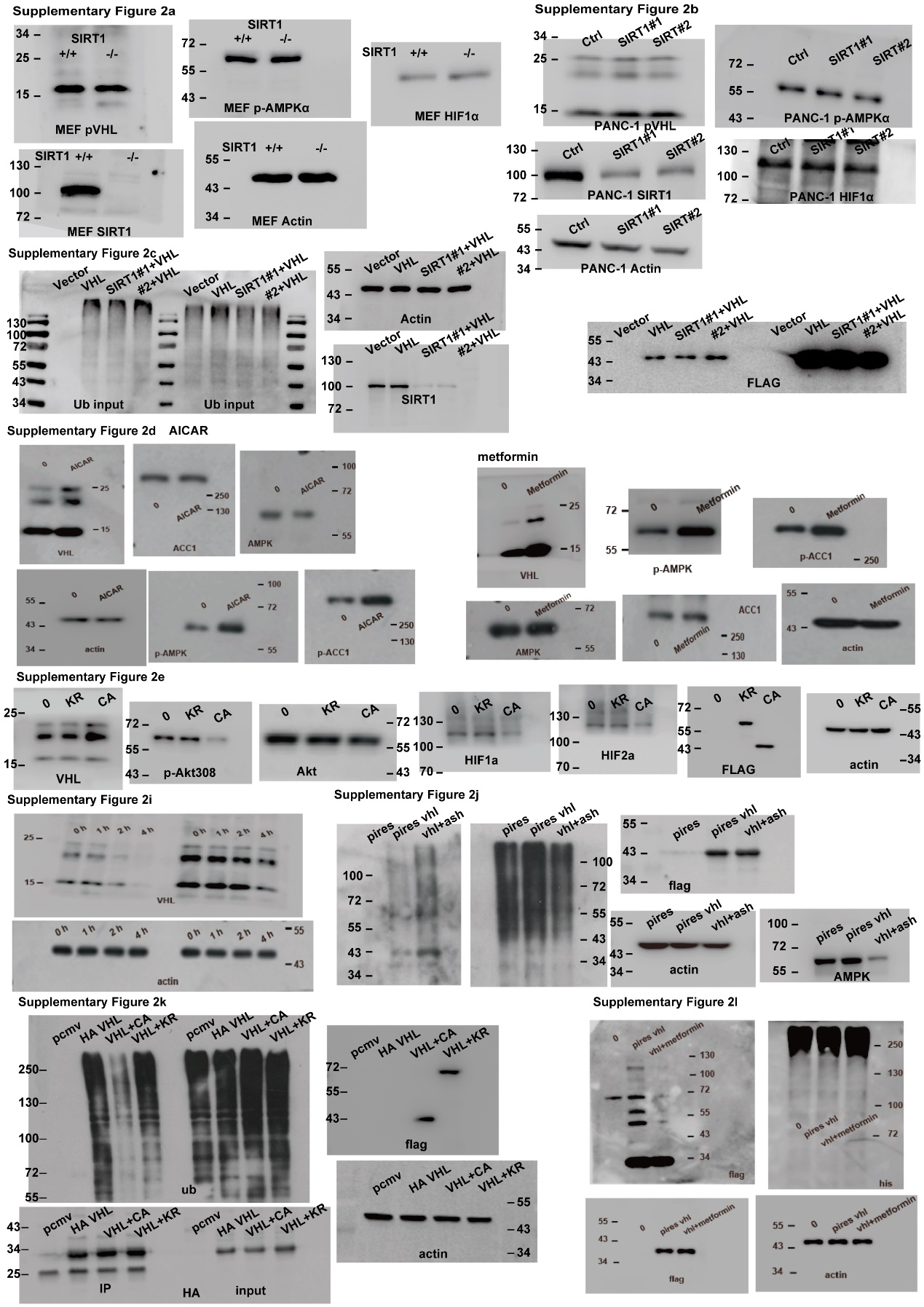


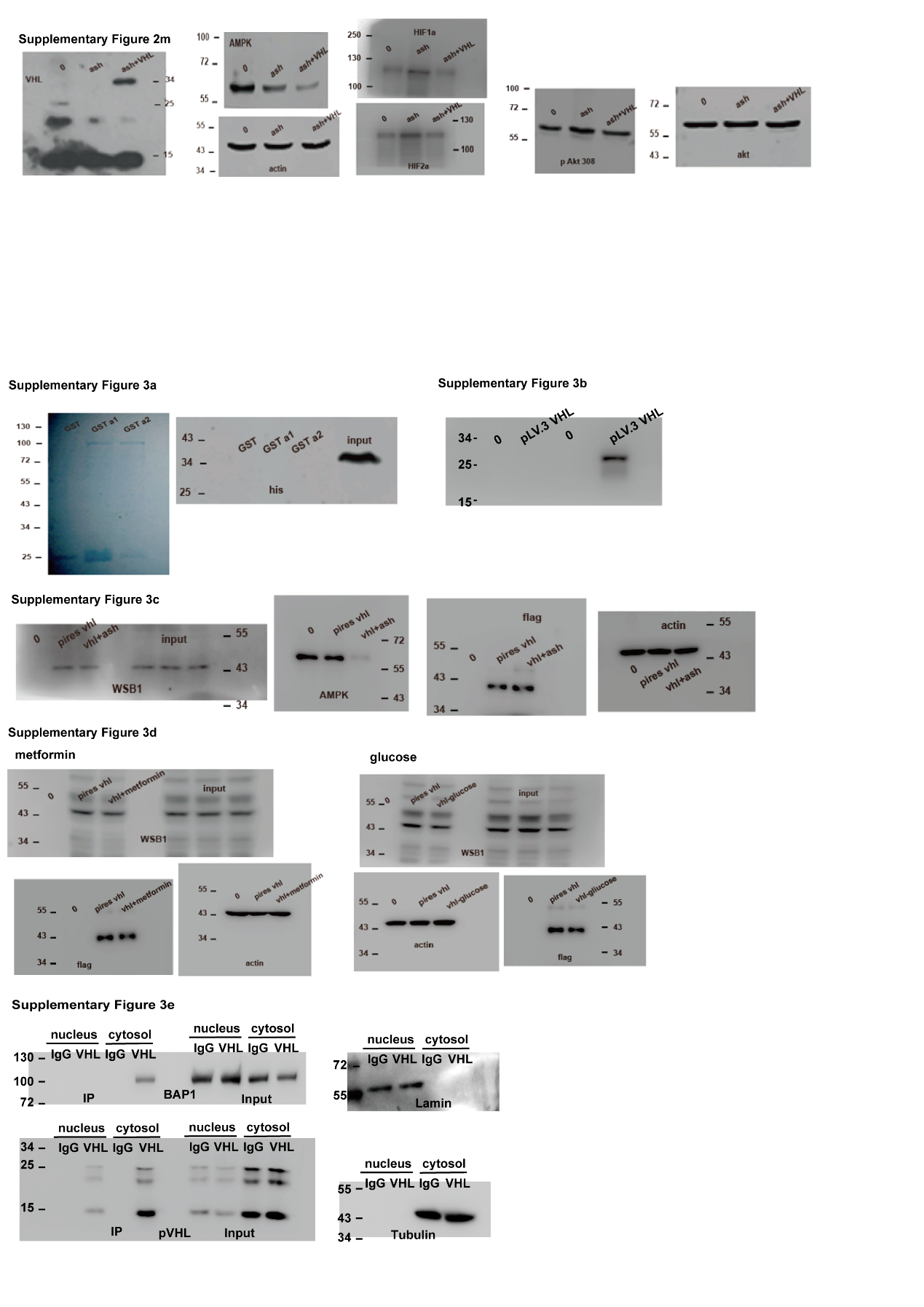


Supplementary Fig. 18: Original scan of the blots presented in the Supplementary Text 1. Related to Supplementary Fig. 2.

**Supplementary Figure 19**


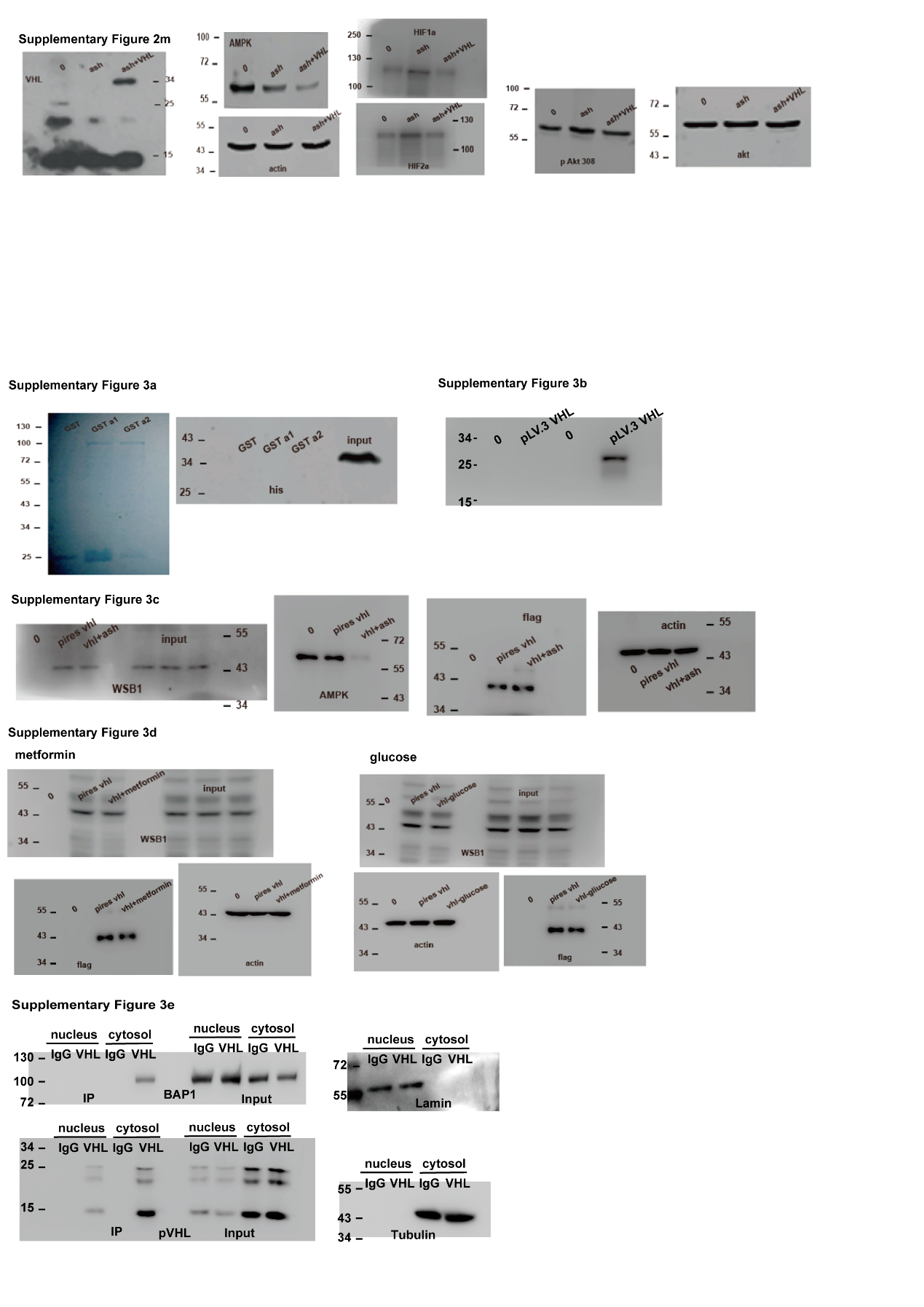


Supplementary Fig. 19: Original scan of the blots presented in the Supplementary Text 1. Related to Supplementary Fig. 3.

**Supplementary Figure 20**


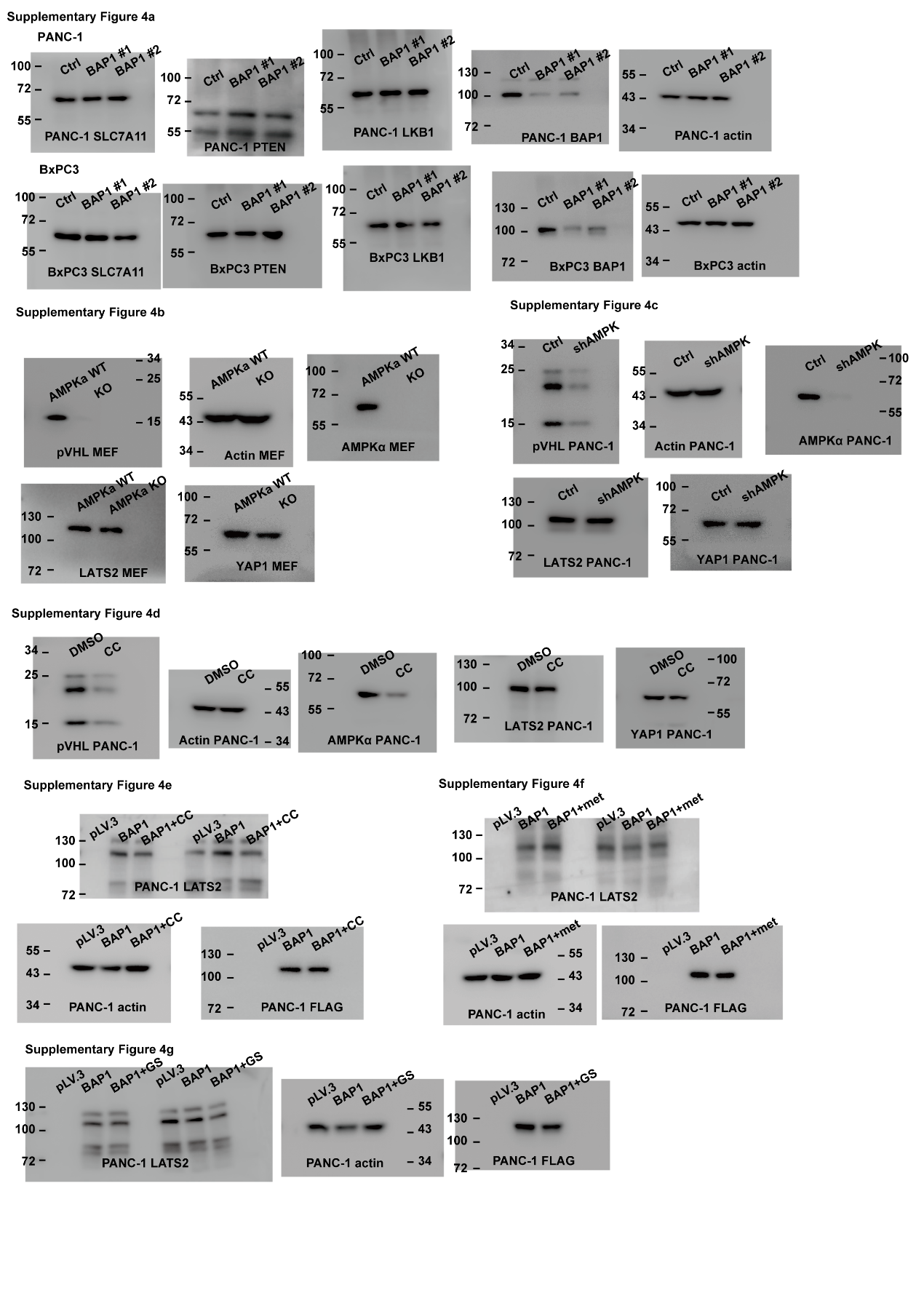


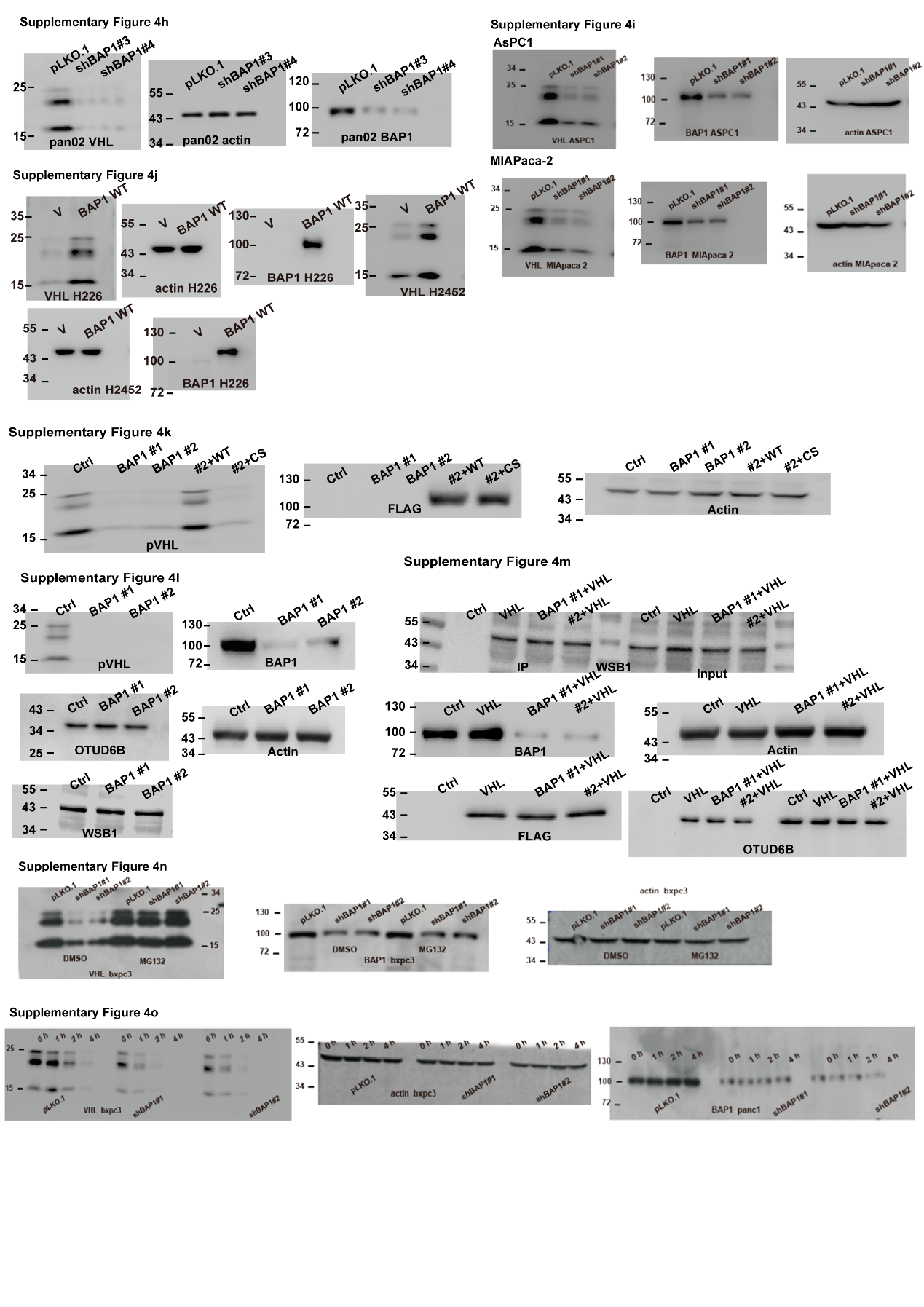


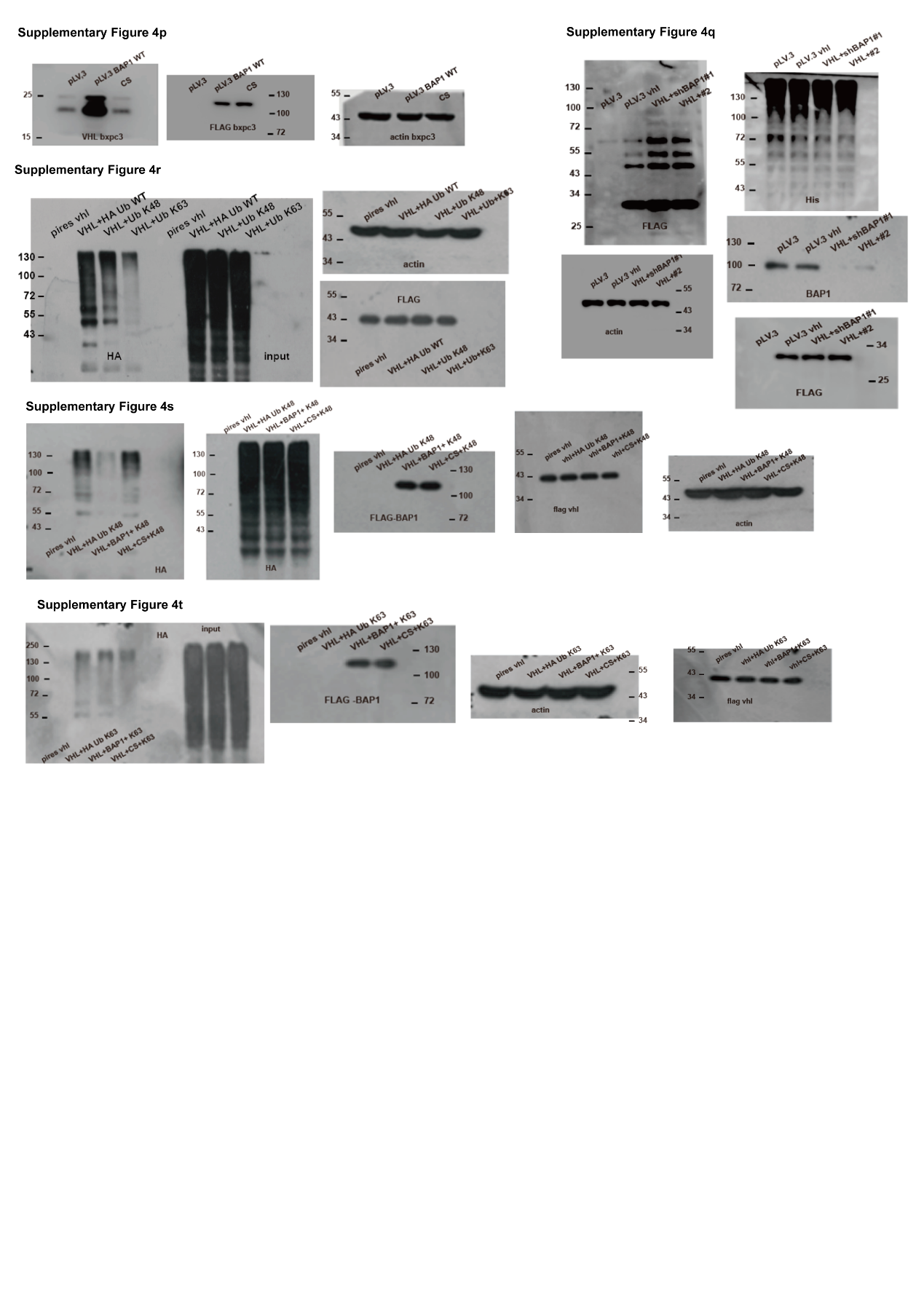


Supplementary Fig. 20: Original scan of the blots presented in the Supplementary Text 1. Related to Supplementary Fig. 4.

**Supplementary Figure 21**


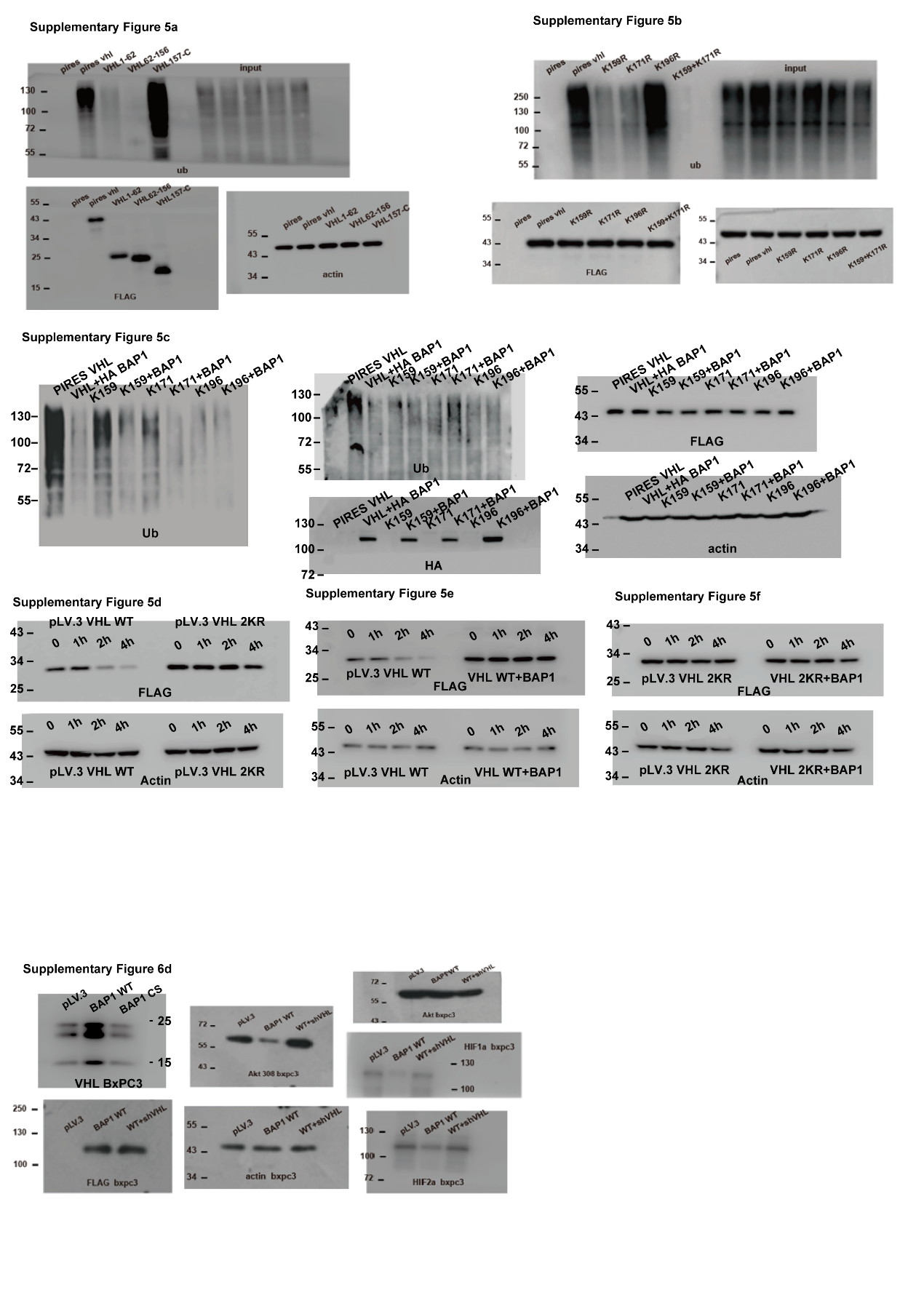


Supplementary Fig. 21: Original scan of the blots presented in the Supplementary Text 1. Related to Supplementary Fig. 5.

**Supplementary Figure 22**


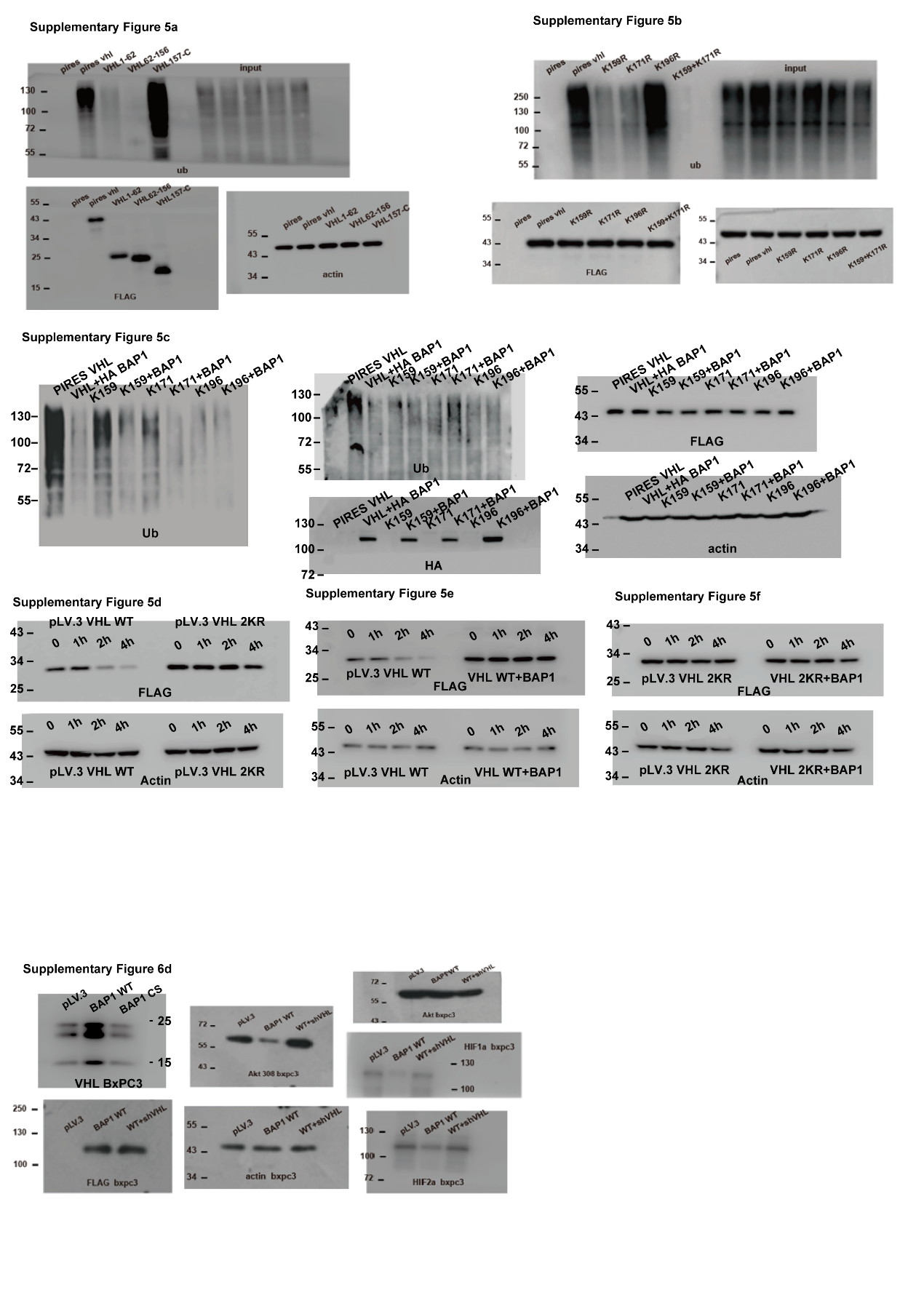


Supplementary Fig. 22: Original scan of the blots presented in the Supplementary Text 1. Related to Supplementary Fig. 6.

**Supplementary Figure 23**


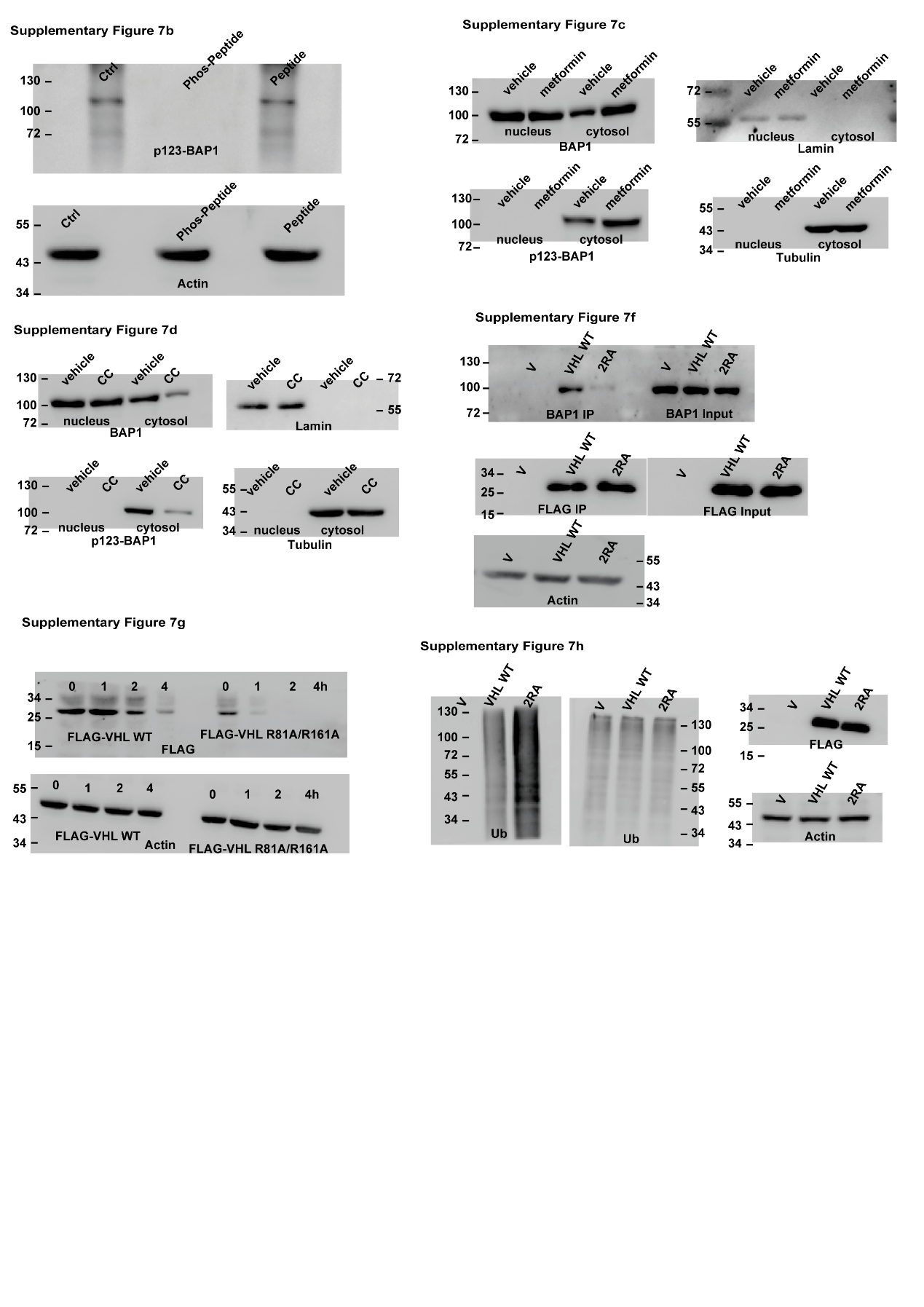


Supplementary Fig. 23: Original scan of the blots presented in the Supplementary Text 1. Related to Supplementary Fig. 7.

**Supplementary Figure 24**


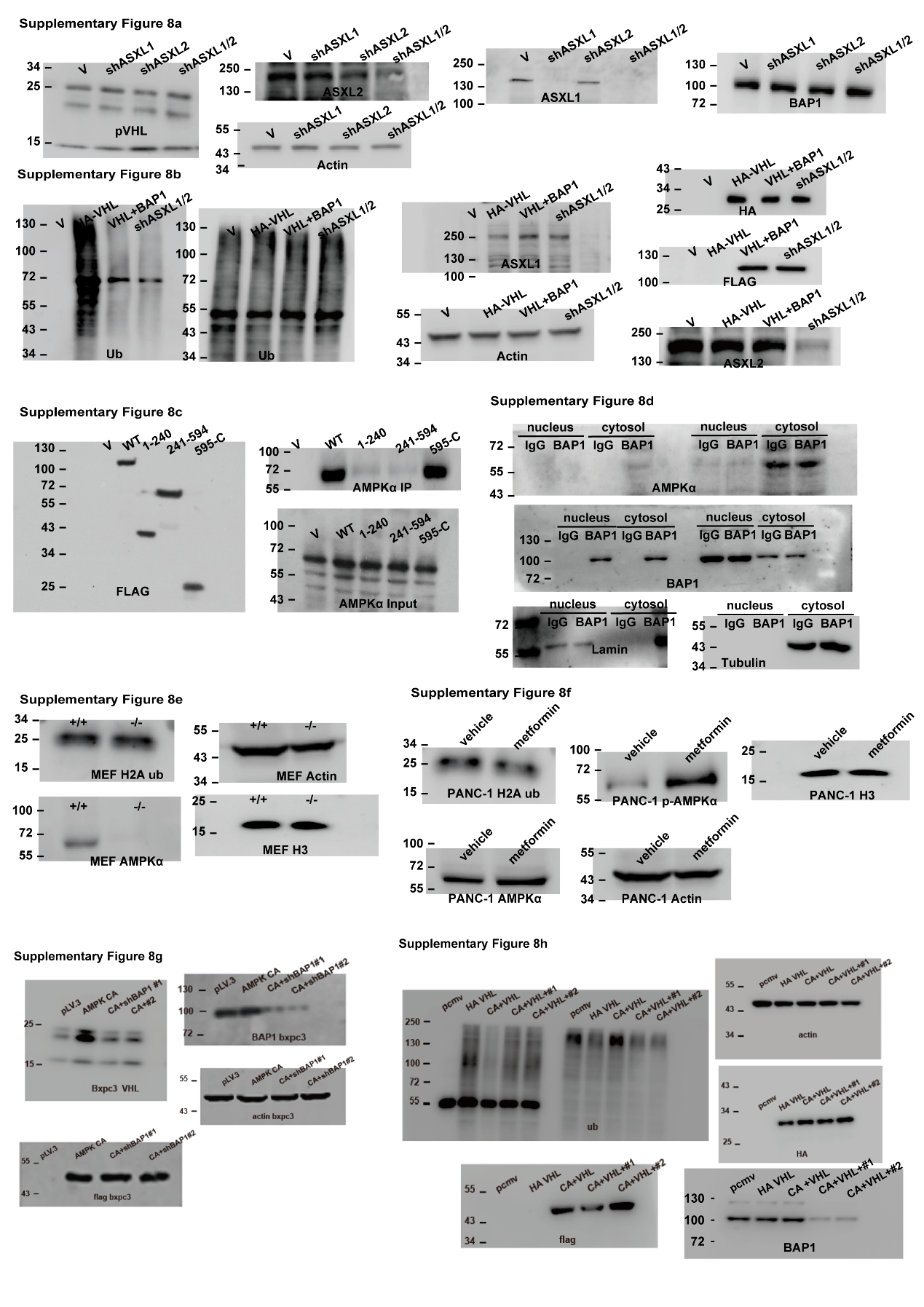


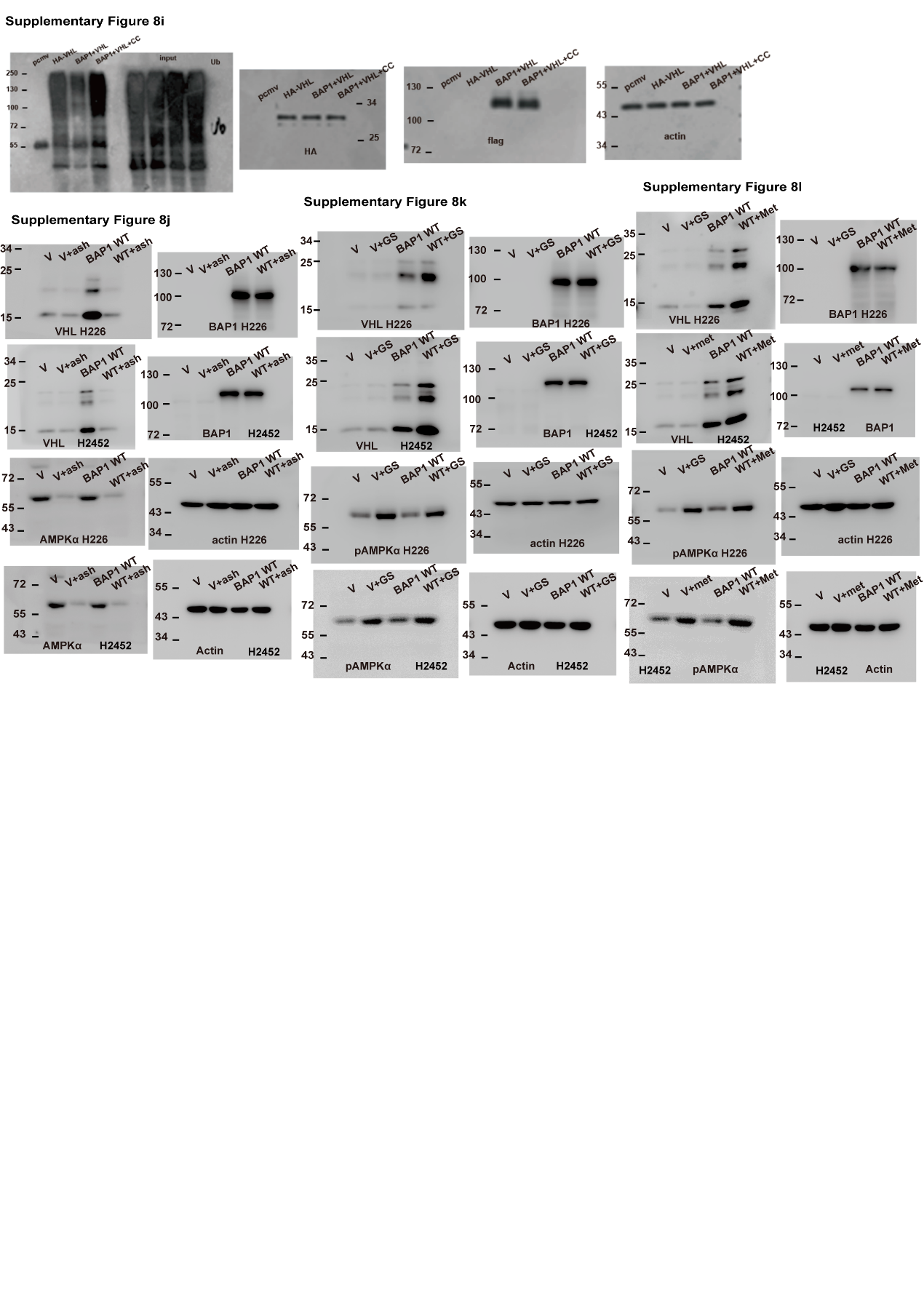


Supplementary Fig. 24: Original scan of the blots presented in the Supplementary Text 1. Related to Supplementary Fig. 8.

**Supplementary Figure 25**


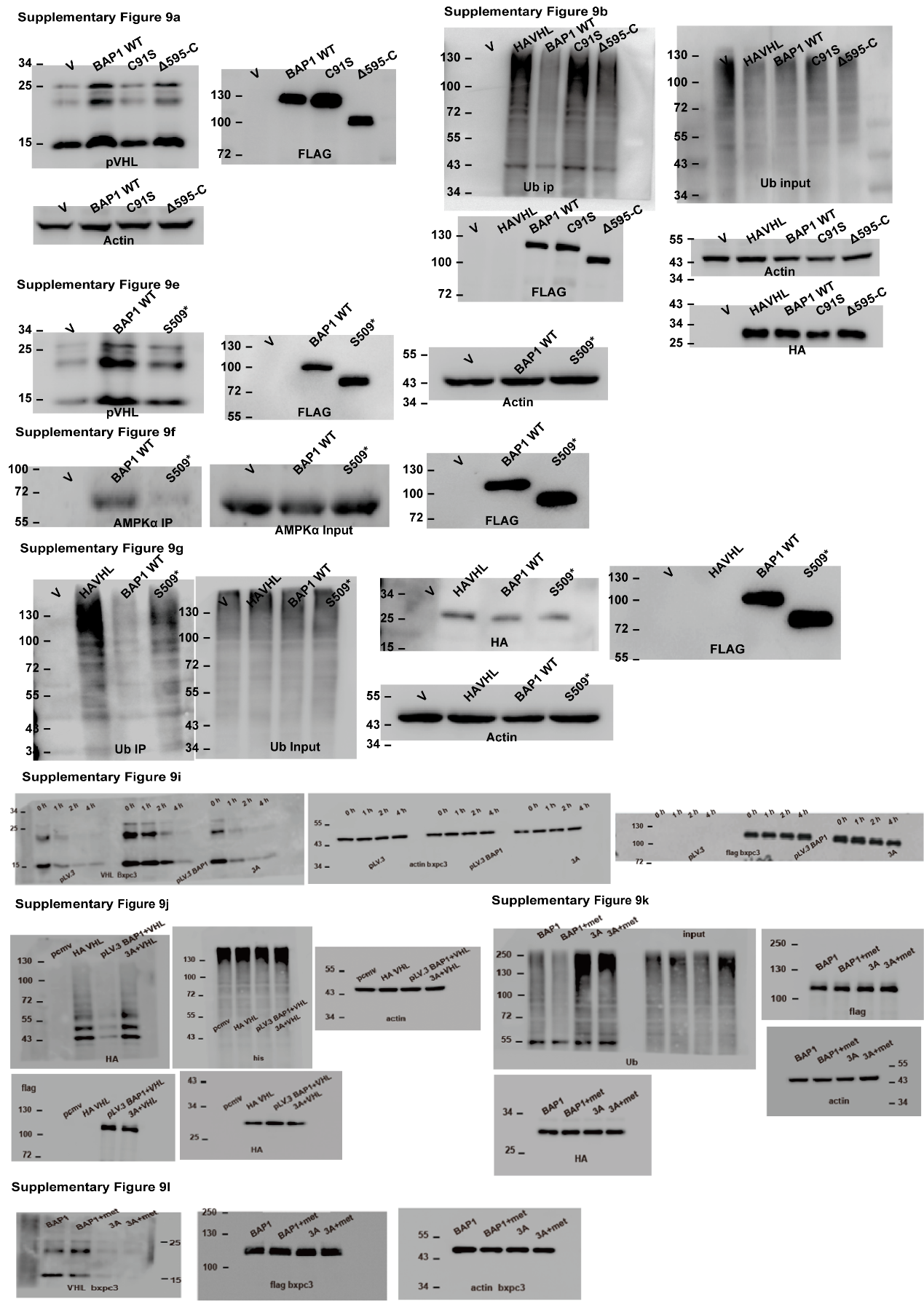


Supplementary Fig. 25: Original scan of the blots presented in the Supplementary Text 1. Related to Supplementary Fig. 9.
